# Global inequities in organ transplantation, 2008–2023: trends, unmet need, and policy implications

**DOI:** 10.1016/j.eclinm.2026.103788

**Published:** 2026-02-12

**Authors:** Peng Hao, Qing He, Haifeng Li, Xiaohong Qiu, Zhonghua Klaus Chen

**Affiliations:** a Surgical Intensive Care Unit (SICU), Sun Yat-Sen Memorial Hospital, Sun Yat-Sen University, Guangzhou, China; b Department of Pediatrics, The Third Affiliated Hospital of Sun Yat-Sen University, Guangzhou, China; c Organ Donation and Transplant Management Center of National Key Clinical Specialty Construction Project, Guangzhou Key Laboratory of Organ Transplantation, Office of Organ Donation and Transplantation, Sun Yat-Sen Memorial Hospital, Sun Yat-Sen University, Guangzhou, China; d Institute of Organ Transplantation, Tongji Hospital, Tongji Medical College, Huazhong University of Science and Technology, The Key Laboratory of Organ Transplantation, Ministry of Education, The NHC Key Laboratory of Organ Transplantation, Chinese Academy of Medical Sciences, The OPO of Tongji Hospital, Wuhan, China

**Keywords:** Organ transplantation, Organ donation, Health equity, Global health, Health policy, Time factors

## Abstract

**Background:**

Solid organ transplantation is the definitive treatment for end-stage organ failure, yet access is highly inequitable worldwide. Comparable long-term evidence across organs, regions, and development settings remains limited.

**Methods:**

Data from the WHO Global Observatory on Donation and Transplantation (GODT) for six solid organs (2008–2023) were analyzed. Per-million population (PMP) rates and the estimated annual percentage change (EAPC) were calculated; disparities by Human Development Index (HDI), WHO regions, and Global Burden of Disease 2021 (GBD 2021) regions were quantified using the slope index of inequality (SII) and concentration index (CI); and transplant capacity gap was estimated by comparing observed volumes with PMP benchmarks from very-high-HDI countries.

**Findings:**

Global transplants rose 76% (101,990–179,091); PMP increased 15.1 → 23.1 (EAPC 2.5%). Kidney transplantation accounted for 65% of all solid organ transplants in 2023, representing the largest share of global activity; lung transplantation showed the fastest relative growth. Two procedures declined globally—pancreas-only transplantation and small-bowel transplantation. Absolute volumes were highest in the USA, China, and India, but PMP ranged from >120 (Spain, USA) to <5 in most low-HDI countries. Growth accrued mainly in very-high-HDI settings, with minimal contribution from low-HDI regions. Japan showed persistently low rates despite very-high-HDI status, whereas Mongolia achieved the world's highest EAPC despite low HDI. Inequality widened by SII (55.9 → 73.9), while CI fell modestly (0.61 → 0.53). Benchmarking indicated the largest transplant capacity gap for kidney (>200,000 procedures), then liver (>80,000) and heart (>30,000); coverage remained <10% in most low-HDI countries.

**Interpretation:**

Global activity increased substantially but gains concentrated in very-high-HDI countries, and inequities persist. Outlier trajectories highlight sociocultural and policy factors beyond economic development. Large benchmark-based gaps—especially for kidney, liver, and heart—remain across low- and middle-HDI settings. Strategic investment in policy, infrastructure, and integration of transplantation within universal health coverage is essential to advance equitable access.

**Funding:**

The Integrated Fund4150102990-58803-0/Prof. Dr. Z.K. Chen; The Fund of Guangzhou Key Laboratory of Organ Transplantation2025A03J4036.

**Research in context**

**Evidence before this study**

We searched Ovid MEDLINE, PubMed, and Embase to identify global evidence on solid organ transplantation and to contextualize existing research. Prior studies have largely focused on single organs, specific regions, or cross-sectional assessments, with limited use of standardized per-million population (PMP) metrics over time. No previous study has systematically quantified global transplantation inequalities using established inequality indices or estimated unmet transplantation need relative to a defined benchmark.

**Added value of this study**

This study provides a comprehensive global assessment of transplantation activity across six solid organs in 194 countries from 2008–2023. We quantified temporal trends using PMP and estimated annual percentage change, and measured inequalities using the slope index of inequality and concentration index across HDI and WHO/GBD21 regions. By benchmarking observed volumes against achievable PMP levels in very-high-HDI countries, we estimated organ-specific transplant capacity gaps and identified distinct national trajectories.

**Implications of all the available evidence**

Despite substantial global growth in transplantation activity, access remains highly unequal across regions and development levels. Large transplant capacity gaps persist, particularly for kidney, liver, and heart transplantation in low-HDI settings, while outlier country trajectories highlight the influence of sociocultural, policy, and health-system factors beyond economic development alone. Strengthening transplantation within universal health coverage frameworks and adopting standardized global monitoring metrics are essential to guide equitable policy and system-level investment.

## Introduction

Solid organ transplantation is a life-saving intervention for patients with end-stage organ failure, offering substantial improvements in survival and quality of life.^1^ Advances in surgery, immunosuppression, and donor management have improved transplant outcomes and access. However, the availability and accessibility of transplantation remain highly variable across countries, driven by disparities in health systems and socioeconomic development. As a result, many patients worldwide—particularly those in low- and middle-income settings—continue to lack access to this essential form of care.^2^^,^^3^

Global monitoring of transplant activity is critical to understanding where progress has occurred and where gaps persist. The World Health Organization (WHO) established the Global Observatory on Donation and Transplantation (GODT) in the early 2000s to support standardized data collection and international benchmarking. Since 2008, the GODT has provided annual country-level data on transplant activity across six major organs—kidney, liver, heart, lung, pancreas, and small bowel—enabling longitudinal analyses of global and regional trends. Despite this, global assessments remain fragmented, often limited to certain regions or organ types.^4^

Previous studies have highlighted inequalities in access to transplantation, particularly for complex procedures that depend on robust deceased donor programs and intensive care infrastructure.^5^ While kidney transplantation is the most widely implemented procedure globally—facilitated in part by the feasibility of living donation—*more resource-intensive* transplants such as lung, pancreas, and small bowel remain highly concentrated in high-income settings.^6^ Reports from Europe and North America have further demonstrated intra-regional variation, often reflecting differences in public policy, legal frameworks, infrastructure, and system organization.^4^ However, these analyses are often limited in geographic scope or time frame, leaving important questions about global progress and equity unanswered.^7^ For example, regional reports provide valuable insights into Europe and North America but do not indicate whether similar patterns occur globally or how disparities manifest in lower-HDI regions.

Health inequality in transplantation also remains poorly quantified. Metrics such as per-million population (PMP) rates and estimated annual percentage change (EAPC) are widely used to describe activity, but few studies have systematically examined socioeconomic gradients in transplant access or identified unmet transplant needs using standard indicators. The role of human development—encompassing education, income, and life expectancy—has received limited attention, despite its recognized influence on broader health outcomes. In this context, an integrated evaluation of transplant trends, disparities, and coverage gaps across countries with varying levels of development is urgently needed.

This study provides the first comprehensive global assessment of transplant activity from 2008–2023, drawing on data from the GODT. An examination of trends across six solid organ types was conducted, along with a quantification of geographic and socioeconomic disparities using Human Development Index (HDI) and Global Burden of Disease (GBD21) classifications, and an estimation of the magnitude of unmet transplant needs. Inequality measures—including the slope index of inequality (SII) and concentration index (CI)—are used to assess the distribution of transplant access over time. Finally, country-level trajectories and regional progress are analyzed to inform future investment and policy prioritization within both epidemiological and health system development frameworks.

The study is explicitly presented as a global observational analysis. Its objectives are: (1) to quantify long-term transplant trends, (2) to evaluate disparities across human development strata, and (3) to estimate organ-specific coverage gaps.

## Methods

Annual country-level transplant activity from 2008–2023 was analyzed using the GODT database, which reports numbers of kidney, liver, heart, lung, pancreas, and small-bowel transplants and official population estimates. The quality of the GODT database has been widely recognized in global transplantation surveillance. Through a long-standing collaboration between the WHO and the Spanish National Transplant Organization (ONT), GODT provides standardized, annually updated national transplant statistics. All data undergo structured validation at the national level before submission, followed by additional consistency checks by GODT. The year 2008 was selected because it marks the beginning of standardized annual reporting across all six solid organs in the GODT; 2023 represents the most recent year available at the time of analysis. Countries were grouped by WHO regions as reported in GODT and by the 21 regions of GBD21. HDI groupings were derived from the 2024 Human Development Report (values available through 2022); because year-to-year variation in HDI is minimal, 2022 values were used to represent 2023 groupings.

Transplant activity was expressed as procedures PMP. Long-term trends were summarized with the EAPC; percent change (PC) over prespecified intervals (e.g., 2008–2023) was also reported as a descriptive contrast. Inequality in transplant distribution across countries was assessed using the SII and the CI. The SII quantifies the absolute gradient in transplant activity across the HDI ranking using a weighted regression, whereas the CI measures relative inequality as twice the covariance between each country's share of global transplant activity and its fractional HDI rank. Isolated gaps in country time series for settings with transplant activity were addressed using linear interpolation with manual review, consistent with GATHER^8^; the completed checklist is in Appendix 1 Table S1. The extent of missing data for key transplant variables is summarized in Appendix 1 Figure S1 and Table S2. Coverage gaps were quantified by comparing observed transplant volumes with expected volumes derived from a prespecified benchmark PMP based on high-performing very-high-HDI countries.^9^ Coverage was defined as the ratio of observed to expected transplant numbers (capped at 1 to indicate complete coverage). The transplant capacity gap (absolute shortfall) for each organ and country was calculated as the difference between expected and observed procedures. Full methodological details and organ-specific benchmark PMP values are provided in Appendix 1 Section 2.6. For global, HDI, and WHO regional trends, APC and EAPC estimates were additionally verified using the NCI Joinpoint Regression Program (version 5.4.0) with a 0-Joinpoint log-linear model; corresponding Joinpoint-derived trend plots are shown in Appendix 1 Figures S2–S4.

All analyses and visualizations were performed in R and Python; robustness was assessed by repeating analyses on the original (non-imputed) data (Appendix 2).

### Ethics approval and consent

This study analyzed publicly available, aggregated, and de-identified country-level data obtained from the GODT. The dataset does not contain any individual-level or personally identifiable information. In accordance with international ethical guidelines for research using publicly available secondary data, formal ethics committee approval and individual informed consent were not required.

### Role of the funding source

The funding sources had no role in the study design, data collection, data analysis, data interpretation, or writing of the report. The corresponding author had full access to all the data in the study and had final responsibility for the decision to submit the manuscript for publication.

## Results

### Global and regional trends in organ transplantation, 2008–2023

From 2008–2023, the global number of solid organ transplants increased by 75.61%, from 101,982–179,091 cases. The population-adjusted transplant rate rose from 15.1–23.1 PMP over the same period, with an EAPC of 2.54% (95% CI: 1.91–3.17) (Table 1). Kidney transplantation remained the most common procedure globally, with PMP rising from 10.4–14.9, accounting for 64.61% of all transplants in 2023 (EAPC 1.92%, 95% CI: 1.21–2.64). Liver, heart, and lung transplants also increased in absolute number and rate, with lung showing the greatest relative increase in PMP (PC +135.96%), followed by liver (PC +108.82%). By contrast, pancreas-only (pancreas transplant alone [PTA] + plus pancreas-after-kidney [PAK]) and small-bowel transplantation remained uncommon and showed overall declines (PC −53.56% and −28.07%, respectively). These patterns highlight pronounced heterogeneity in both the scale and direction of organ-specific transplant activity (Appendix 1 Tables S3–S11).

**Table 1:** Trends in the number and rate of solid organ transplants by global region, 2008–2023, including percent change (PC) and estimated annual percentage change (EAPC) with 95% confidence intervals.

Region classification	2008	PMP	2023	PMP	2008–2023	
	Total transplants		Total transplants		PC (%)	EAPC (%, 95% CI)
**Global**	10,1982	15.1	179,091	23.1	75.61	2.54 (1.91–3.18)
**Human development index**						
Very high	65,819	58.5	112,191	68.2	70.45	−1.69 (−3.03 to −0.32)
High	17,123	16.2	44,666	15.8	160.85	−1.23 (−3.14 to 0.71)
Medium	17,967	5.4	19,451	9.1	8.26	−0.67 (−4.23 to 3.02)
Low	1073	0.9	2783	2.5	159.37	5.33 (−2.00 to 13.21)
**World health organization region**						
Africa	563	0.7	806	0.8	43.16	−0.19 (−7.36 to 7.54)
America	41,542	45.4	68,598	66.2	65.13	−2.83 (−5.82 to 0.26)
Eastern Mediterranean	5702	10.0	9575	12.9	67.92	1.43 (−2.08 to 5.07)
Europe	32,492	36.5	46,019	49.8	41.63	1.20 (−0.78 to 3.22)
South-East Asia	7132	4.0	20,150	9.8	182.53	14.04 (6.23–22.42)
Western Pacific	14,551	8.2	33,943	17.6	133.27	5.40 (0.89–10.12)
**Global burden of disease region**						
High-income Asia Pacific	3697	20.5	6707	37.0	81.42	1.65 (−2.35 to 5.82)
High-income North America	30,075	87.9	50,946	134.5	69.40	2.68 (1.08–4.30)
Western Europe	25,454	63.0	32,036	75.5	25.86	0.80 (−0.50 to 2.13)
Australasia	1376	54.6	1988	62.9	44.48	2.00 (0.74–3.28)
Andean Latin America	1157	11.8	1947	16.6	68.28	1.19 (−3.47 to 6.07)
Tropical Latin America	5118	26.4	8725	40.3	70.48	1.63 (0.41–2.87)
Central Latin America	2692	17.3	3777	20.2	40.30	−5.07 (−9.66 to −0.25)
Southern Latin America	1918	31.9	3005	43.7	56.67	1.85 (−0.32 to 4.07)
Caribbean	582	8.9	198	2.9	−65.98	−6.76 (−11.03 to −2.27)
Central Europe	3256	25.0	4601	36.4	41.31	−1.31 (−4.28 to 1.75)
Eastern Europe	900	4.6	3043	15.8	238.11	7.26 (0.69–14.25)
Central Asia	272	3.6	409	4.8	50.37	7.32 (3.26–11.54)
North Africa and Middle East	7652	16.1	13.289	23.0	73.67	1.84 (−1.70 to 5.51)
South Asia	6979	4.4	21,281	11.0	204.93	7.77 (1.34–14.62)
Southeast Asia	2147	3.7	2380	3.7	10.85	11.46 (5.20–18.09)
East Asia	8259	6.1	240,46	16.4	191.15	14.46 (10.00–19.11)
Oceania	0	0.0	0	0.0	–	–
Western Sub-Saharan Africa	15	0.1	253	0.7	1586.67	21.10 (15.90–26.54)
Eastern Sub-Saharan Africa	128	0.4	69	0.2	−46.09	−11.82 (−20.42 to −2.29)
Central Sub-Saharan Africa	0	0.0	0	0.0	–	–
Southern Sub-Saharan Africa	305	5.5	391	6.0	28.20	0.21 (−0.55 to 0.97)

PMP = per-million population; PC = percent change; EAPC = estimated annual percentage change; CI = confidence interval.

PC (%) indicates the percentage change in PMP from 2008 to 2023. EAPC and corresponding 95% CIs were calculated by fitting a linear regression model to the natural logarithm of PMP across years (log(PMP) ∼ Year).

A positive EAPC with a 95% CI not crossing zero indicates a statistically significant increasing trend; a negative EAPC with a 95% CI not crossing zero indicates a decreasing trend.

**Fig. 1:**
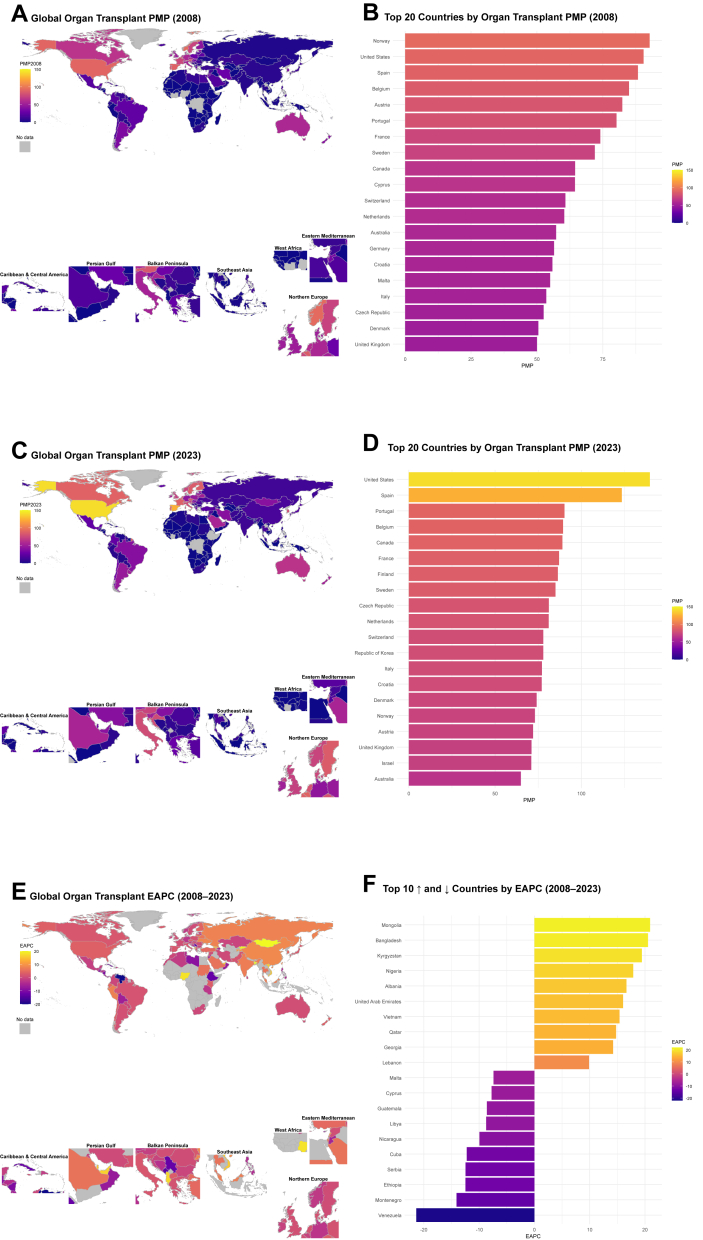
**Global patterns and trends in solid organ transplantation, 2008–2023**. (A) Global distribution of solid organ transplant rates (per-million population [PMP]) in 2008. (B) Top 20 countries by PMP in 2008. (C) Global distribution of transplant rates (PMP) in 2023. (D) Top 20 countries by PMP in 2023. (E) Estimated annual percentage change (EAPC) in transplant rates (PMP) between 2008 and 2023. (F) Ten countries with the largest increases and decreases in EAPC during 2008–2023. Grey shading indicates countries without data reported to the Global Observatory on Donation and Transplantation. PMP was calculated as the total number of solid organ transplants divided by the population.

Across the study period (2008–2023), the United States, China, and India reported among the highest transplant volumes worldwide. In 2008, these countries reported 27,934, 8,255, and 5855 transplants, respectively, rising to 47,492, 23,905, and 18,378 by 2023. Notably, they also recorded the largest absolute increases in transplant volume over the study period, with gains of 19,558 in the United States, 15,650 in China, and 12,523 in India. However, given their large population sizes, population-adjusted transplant rates in China and India remained well below the global average. In 2023, high PMP transplant rates were reported in the United States (139.7 PMP), Spain (123.4 PMP), and Portugal (90.2 PMP). This represents a shift from 2008, when Norway (92.8 PMP), the United States (90.5 PMP), and Spain (88.3 PMP) had the highest transplant rates globally. Substantial heterogeneity in temporal trends was observed across countries. The most rapid increases in transplant rates were observed in Mongolia (EAPC 20.92%), Bangladesh (20.54%), and Kyrgyzstan (19.44%). Conversely, Venezuela (−21.36%), Montenegro (−14.08%), and Ethiopia (−12.47%) experienced the steepest declines during the same period (Appendix 1 Table S12). These cross-country differences and temporal patterns are illustrated in Fig. 1.

Between 2008 and 2023, a total of 114 countries reported performing at least one type of solid organ transplant (Appendix 1 Table S12). Among these, kidney transplantation was the most widely implemented, performed in all 114 countries. Liver transplants were reported in 94 countries, heart in 71, lung in 60, pancreas in 59, and small bowel in 35, over the study period. While many countries provided only one or two types of transplant procedures, typically with low annual volumes, global capacity for comprehensive transplant services increased substantially over the study period. The number of countries capable of performing all six major solid organ transplants rose from 16 during 2008–2010 to 27 by 2021–2023 (Appendix 1 Tables S13–S16). The United States consistently demonstrated global leadership in transplantation activity, both in absolute terms and when adjusted for population size. In 2023, it recorded the highest absolute number of procedures across all six major solid organ types. Furthermore, when expressed as PMP, the United States also exhibited the highest levels for kidney (82.8), liver (31.4), and heart (13.5) transplants. However, other countries led in PMP for specific procedures: Austria for lung (13.8 PMP), Finland for pancreas (5.1 PMP), and the United Kingdom for small bowel transplants (0.3 PMP), highlighting diverse national strengths in organ-specific transplant capacity (Appendix 1 Tables S17–S22). Beyond volumes and rates, donor-source composition also varied across countries; for example, in Iran, kidney transplantation was predominantly from living donors in 2008 (80.0%), declining to 49.0% by 2023 (Appendix 1 Figure S5).

### Geographic and regional variations in transplant trends

Persistent disparities in transplant activity were observed across HDI and WHO regions throughout the study period. From 2008–2023, countries with very high HDI consistently maintained the highest transplant rates, fluctuating between 55 and 65 PMP, while high HDI countries ranged between 10 and 20 PMP. Medium HDI countries remained between 5 and 10 PMP, and low HDI countries consistently fell below 5 PMP (Table 1; Appendix 1 Figure S6A). Within the very-high-HDI group, Japan was a notable outlier, with transplant rates substantially lower than those of HDI-comparable countries and, for most years from 2008–2023, also below the global average (Appendix 1 Figure S7). In China (classified as high-HDI from 2011), overall transplant activity showed stepwise increases from 2015 onwards, driven particularly by kidney, liver, heart, and lung transplantation (Appendix 1 Figure S8). A similar pattern was evident across WHO regions. The Americas and Europe maintained leading positions in transplant activity, reaching 66.2 PMP and 49.8 PMP, respectively, in 2023. In contrast, transplant rates in the South-East Asia and the African Region remained markedly lower, all below 10 PMP. Notably, the African Region reported consistently minimal transplant activity throughout the study period, with PMP values remaining close to zero, highlighting profound and persistent inequities in transplant access across global regions (Table 1; Appendix 1 Figure S6B). Importantly, all regions experienced a visible decline in transplant rates in 2020, likely attributable to the widespread disruptions caused by the COVID-19 pandemic. Despite recovery in subsequent years, the transient contraction in global transplant activity underscores the vulnerability of transplant systems to public health emergencies.

Building on these observed disparities, contributions to global transplant growth also varied significantly across HDI groups. Between 2008 and 2023, very high HDI countries accounted for 62.6% of the global increase in transplant activity, followed by high HDI countries (24.9%). In contrast, medium and low HDI countries contributed only 10.9% and 1.6%, respectively (Appendix 1 Figure S9), suggesting that the expansion of transplant services over the past 15 years was largely driven by countries with pre-existing infrastructure and resources.

To further illustrate regional disparities, organ-specific heatmaps revealed striking differences in transplant practices across the globe. In 2023, kidney transplant rates ranged from 79.8 PMP in high-income North America to below 5 PMP in much of sub-Saharan Africa. Liver and heart transplant services remained largely concentrated in high-income regions, while lung transplantation largely concentrated in Australasia, Europe, and North America. Pancreas and small bowel transplants were exceptionally rare across all regions, regardless of income (Appendix 1 Figure S10; Tables S4–S11).

### National trajectories and inequity in transplant access

**Fig. 2:**
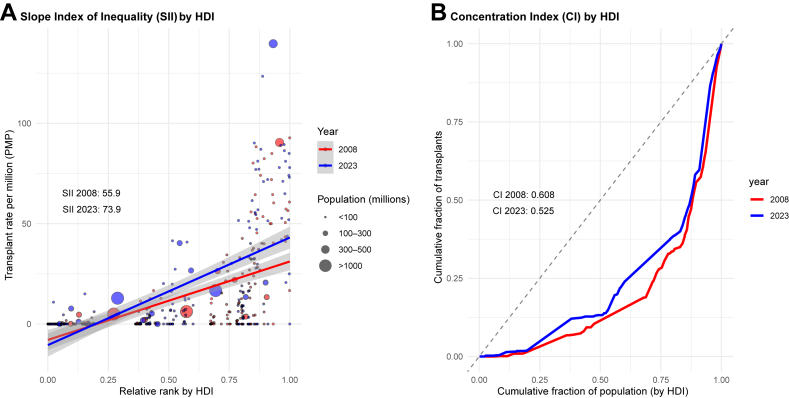
**Global inequality in organ transplantation by HDI, 2008 and 2023**. (A) Slope Index of Inequality (SII) in transplant rate per-million population (PMP) by Human Development Index (HDI) rank, comparing 2008 and 2023. Each circle represents a country, weighted by population size. Regression lines and 95% CIs are shown, with SII values indicated for 2008 and 2023. (B) Concentration Index (CI) of transplant distribution by HDI rank in 2008 and 2023. The 45-degree line indicates perfect equality. CI values are shown for both years.

Substantial inequalities in transplant access persisted and, in some cases, worsened across countries during the study period. The SII by HDI increased from 55.9 in 2008 to 73.9 in 2023, indicating a widening disparity between the most and least developed countries (Fig. 2A). By contrast, the CI declined from 0.608–0.525, reflecting a modest shift in the distribution of transplant activity toward lower-HDI countries (Fig. 2B), although overall inequity remained substantial.

These global patterns were mirrored in national trajectories (Appendix 1 Figure S11A–B). Countries with very high HDI generally exhibited consistent growth in transplant rates from 2008–2023, often surpassing 50 PMP by 2023. In contrast, most countries with HDI below 0.75 remained under 10 PMP, with those below 0.5—predominantly in sub-Saharan Africa—tightly clustered near zero, reflecting a pronounced “poverty concentration” effect. This underperformance was especially marked in high-population countries such as Bangladesh, Nigeria, and Ethiopia. Growth trends further highlighted inequities: Mongolia achieved notable progress, with transplant rates exceeding 40 PMP in 2023 and an EAPC exceeding 20%, whereas many low-HDI countries, including Ghana, United Republic of Tanzania, and Ethiopia, continued to show stagnant trends and negligible transplant activity. These findings underscore the urgent need for policies to address entrenched structural inequities in transplant access worldwide.

### Organ-specific coverage and global transplant capacity gap

**Fig. 3:**
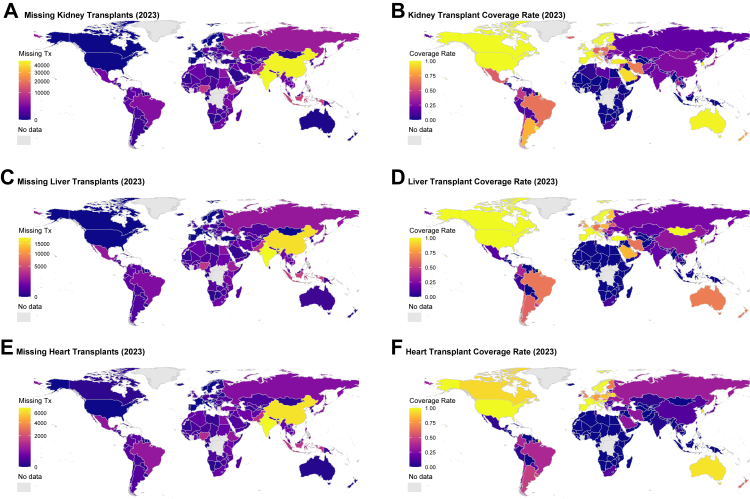
**Global distribution of missing kidney, liver, and heart transplants and corresponding coverage rates in 2023**. (A) Missing kidney transplants. (B) Kidney transplant coverage rate. (C) Missing liver transplants. (D) Liver transplant coverage rate. (E) Missing heart transplants. (F) Heart transplant coverage rate. Missing transplant numbers were calculated as the difference between the expected number of transplants and the observed number. Coverage rate was defined as observed transplants divided by expected transplants, capped at 1.0. Grey shading indicates countries without data.

In 2023, marked disparities in organ-specific transplant coverage were observed across countries and regions. Among all solid organs, kidney transplantation showed the highest global burden of transplant capacity gap, with an estimated gap of more than 200,000 transplants when compared with ideal PMP benchmarks. This shortfall was predominantly concentrated in low- and middle-income countries, particularly in Asia, sub-Saharan Africa, and Latin America. India alone accounted for nearly 50,000 missing kidney transplants. By contrast, high-income countries such as the United States and Spain achieved near-complete coverage relative to the ideal PMP standard (Fig. 3A and B; Appendix 1 Table S23). Liver and heart transplant needs were also significantly unmet, with over 80,000 and nearly 30,000 transplant capacity gap globally, respectively (Fig. 3C and E; Appendix 1 Tables S24–S25). Liver transplant coverage showed moderate performance in high-HDI countries (e.g., Brazil [66%], China [30%]), while most countries in Africa, Southeast Asia, and the Middle East remained below 10% coverage (Fig. 3D). For heart transplantation, coverage exceeded 75% only in a few countries (e.g., Republic of Korea, the USA, and selected EU nations such as Spain and France), with most countries entirely lacking transplant programs (Appendix 1 Table S25). These disparities highlight persistent inequities in organ-specific transplant access, particularly for more complex procedures such as liver and heart transplantation.

**Fig. 4:**
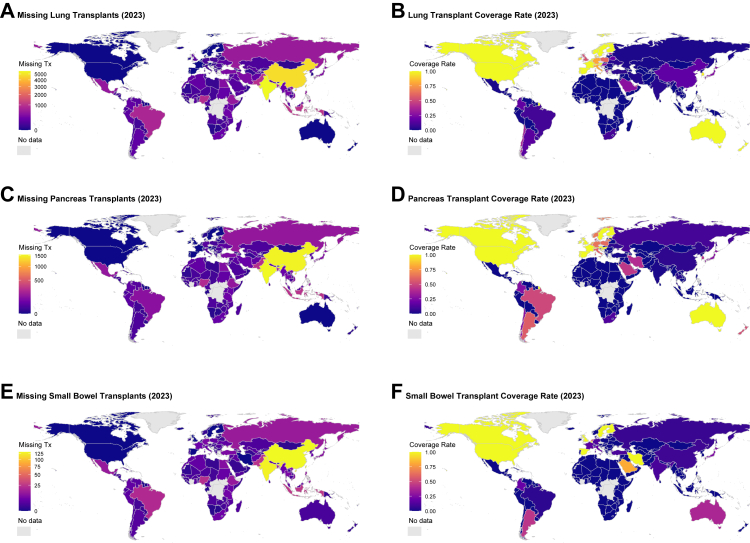
**Global distribution of missing lung, pancreas, and small bowel transplants and corresponding coverage rates in 2023.** (A) Missing lung transplants. (B) Lung transplant coverage rate. (C) Missing pancreas transplants. (D) Pancreas transplant coverage rate. (E) Missing small bowel transplants. (F) Small bowel transplant coverage rate. Missing transplant numbers were calculated as the difference between the expected number of transplants and the observed number. Coverage rate was defined as observed transplants divided by expected transplants, capped at 1.0. Grey shading indicates countries without data.

The situation was more severe for lung, pancreas, and small bowel transplantation (Fig. 4; Appendix 1 Tables S26–S28). These organ types showed limited availability even in high-HDI settings, with nearly all low-HDI countries reporting zero transplants in 2023. Global coverage for lung transplantation was highly polarized: a few countries in Western Europe and North America reported full coverage, while the majority of countries, especially in Africa and Asia, reported no activity (Fig. 4A and B). Pancreas and small bowel transplants, being highly specialized procedures, exhibited lower global diffusion.

A comparative analysis of the actual versus ideal transplant volume by HDI strata highlighted the magnitude of the global transplant gap. For all six organs, countries classified as high, medium, or low HDI collectively accounted for the majority of the transplant capacity gap despite their large populations. For example, high-HDI countries performed less than one-third of the kidney transplants needed to match the ideal benchmark, and medium-to low-HDI countries achieved only a fraction of that level. Similar disparities were evident across liver, heart, lung, pancreas, and small bowel transplantation types (Appendix 1 Figure S12), underscoring systemic limitations in service availability, donor infrastructure, and technical capacity.

Sensitivity analyses using original (non-imputed) data were consistent with the primary findings (Appendix 2)

## Discussion

This study provides a comprehensive and up-to-date global assessment of solid organ transplantation trends and disparities across 194 countries and territories from 2008–2023. Data from the GODT demonstrate that global transplant activity increased substantially over the past 15 years, with marked growth in kidney, liver, heart, and lung procedures. However, this expansion was unevenly distributed, with very high-HDI countries contributing the vast majority of growth while lower-HDI countries experienced stagnation or decline. Profound inequalities in transplant access persisted or worsened, particularly in low-resource settings such as sub-Saharan Africa and South Asia. Our findings highlight persistent gaps in transplant coverage across all six major organs, with kidney transplantation showing the largest global shortfall. Many countries with large populations and low transplant rates—such as India, Nigeria, and Bangladesh—continue to disproportionately drive global inequity. Taken together, our results underscore the urgent need for coordinated global action to strengthen transplant capacity and reduce structural inequalities in access to life-saving organ replacement therapies. Because EAPC and CI capture different dimensions of transplantation patterns, with EAPC reflecting overall temporal growth and CI describing cross-country distribution, global increases in transplant activity may coexist with modest reductions in inequality, consistent with our findings.

Our findings build upon and contextualize earlier regional and thematic investigations of transplantation inequity. While previous reports have described trends in single organ types within selected regions, few studies have systematically disaggregated long-term global patterns by organ type, region, and socioeconomic status.^2^, ^3^, ^4^ A 2023 ERA–GODT analysis of 40 European countries revealed a clear east–west gradient in kidney transplant activity, with Western Europe achieving higher deceased donor transplant rates than Eastern counterparts, despite operating within broadly similar European regulatory and oversight frameworks.^4^ Our global results confirm that similar gradients persist at a broader scale, with low- and middle-HDI regions consistently underperforming relative to very high- and high-HDI peers.^2^^,^^3^ Notably, even among very high-HDI countries, significant exceptions exist. Japan has maintained persistently low deceased organ donation rates despite its high level of human development. This trajectory originated with the 1968 Wada case, which led to enduring public distrust and legal uncertainty regarding brain death.^10^ Consequently, deceased donation remained minimal for decades, with transplantation practice relying largely on living donors. Although the 1997 Organ Transplant Law formally recognized brain death, strict consent requirements continued to limit donation activity, and a 2009 amendment allowing pediatric donation resulted in only modest increases. Beyond legal and sociocultural factors, the widespread availability of long-term dialysis within Japan's advanced kidney replacement therapy system has reduced the clinical demand for kidney transplantation. Collectively, these factors account for Japan's distinctive position among high-HDI countries with respect to deceased organ donation.^11^ Beyond Japan, other national contexts further illustrate how sociocultural and institutional mechanisms shape organ donation trajectories. Iran's state-regulated compensated living-unrelated donor program long facilitated one of the world's highest rates of living kidney donations.^12^ More recently, religious endorsement of brain death certification and supportive legislation have promoted a gradual shift toward deceased donation. Similarly, in China, the post-2015 transition to a voluntary citizen-based deceased organ donation system, aligned with national allocation standards, has been associated with steady growth in transplant activity.^13^

A range of factors likely contributes to these disparities. Unlike kidney transplantation—which is often technically simpler and can be supported by both living and deceased donation—liver and heart transplants rely more heavily on robust intensive care systems and coordinated deceased donor programs, which remain limited in resource-constrained settings. Such systems typically require adequate ICU capacity, national donor registries, well-trained coordination and retrieval teams, and access to modern perfusion and preservation technologies. For example, in the United States, longstanding deceased donor frameworks such as the OPTN/SRTR system have played a critical role in sustaining transplant volume and expanding donor eligibility criteria over time.^7^ Similarly, efforts to incorporate circulatory death donors (DCD) into heart and lung transplantation have helped partially alleviate supply constraints in select high-income countries.^14^ In contrast, many medium- and low-HDI countries face compounding challenges: low public awareness of organ donation, limited national transplant registries, and under-resourced ICU infrastructure. In many countries, organ donation laws remain underdeveloped or inconsistently enforced, shaped by cultural barriers, limitations in the legal recognition and implementation of brain-death determination, and infrastructural constraints as highlighted in recent ethical and legal analyses.^15^, ^16^, ^17^, ^18^ The COVID-19 pandemic further disrupted transplant systems worldwide, particularly in countries with already fragile health infrastructure.^19^, ^20^, ^21^, ^22^ Our analysis shows that complex procedures such as lung, pancreas, and small bowel transplantation remain restricted to very high-HDI countries,^6^^,^^23^^,^^24^ reinforcing concerns about global surgical capacity gaps raised in prior Lancet Commission reports.^25^ Notably, two organ types deviated from the prevailing upward trend: PTA and small-bowel transplantation. Both remained relatively uncommon and exhibited overall declines during the study period. The decline in solitary pancreas transplantation likely reflects its increasingly unfavorable risk–benefit profile under lifelong immunosuppression, alongside advances in diabetes management—such as continuous glucose monitoring, hybrid closed-loop insulin delivery systems, GLP-1 receptor agonists, and SGLT2 inhibitors—which have reduced the clinical necessity for PTA.^26^ In contrast, simultaneous pancreas–kidney (SPK) transplantation has remained broadly stable.^27^ Similarly, for small-bowel transplantation, improvements in the management of intestinal failure—including safer home parenteral nutrition protocols (with enhanced catheter care and lipid emulsions) and the adoption of GLP-2 analogues such as teduglutide—have diminished the indication for transplantation for many patients.^28^^,^^29^

At the system level, the role of national policies and targeted investment emerges as a key modifier. China and Brazil, although not classified as very-high-HDI countries, demonstrated substantial absolute volumes in liver and heart transplantation, suggesting that targeted system-level investment and coordinated national policies can help reduce structural barriers.^30^ Similarly, Mongolia, a country with relatively low HDI, achieved the world's highest EAPC in transplant activity during the study period. This rapid growth has been facilitated by sustained financial support from international foundations and technical collaboration with overseas academic centers—providing clinical training, workforce development, and capacity-building in organ procurement and brain-death determination. These strategic international partnerships demonstrate that targeted external investment can yield transformative progress even in resource-constrained contexts.^31^ Such experiences provide replicable models for other low- and middle-HDI countries. These models share several core features—including sustained governmental commitment, dedicated financing mechanisms, coordinated national donation and retrieval systems, and structured training or capacity-building partnerships—which appear to be the most influential components enabling expansion of transplantation services in resource-constrained settings. In contrast, some countries exhibited declines or persistent stagnation in transplant activity during the study period. In Montenegro, transplant volumes remained low and showed little sustained progress, likely reflecting the limited maturity of national transplant systems, small case volumes, and constrained health-system capacity.^32^ Similarly, in Ethiopia, persistently low transplantation rates align with longstanding shortages in intensive care resources, surgical workforce, and critical care infrastructure documented across sub-Saharan Africa, which restrict the feasibility of complex procedures such as organ transplantation.^33^ However, as shown in recent regional studies, national averages may obscure within-country inequities, and further efforts are needed to ensure equitable access across population groups.^2^^,^^3^^,^^34^ Moreover, because HDI does not capture within-country wealth inequality, transplant access may vary widely across socioeconomic groups; future assessments incorporating measures such as the GINI coefficient could help further characterize these gradients.

These findings have direct implications for global health equity and policy. Given the life-saving potential of transplantation and persistent under-provision in many low- and middle-HDI settings, international investment in infrastructure, workforce training, and public awareness is urgently needed. Strengthening national registries, developing transnational data systems to enable comparable benchmarking, improving donor identification, and harmonized legal frameworks could narrow disparities, while integration within universal health coverage and essential surgical care would support equitable access. In line with the ESOT–Lancet Commission, the aim is to move transplantation from high-end care to a universally accessible part of the health system.^9^^,^^35^

Our study has several limitations. First, data on transplantation volumes were obtained from national reports submitted to the GODT, which may vary in completeness and quality across countries and over time. In addition, the GODT dataset does not include graft- or patient-level outcomes, which limits the assessment of transplant service quality. Furthermore, transplant procedures associated with ethical concerns, such as transplant tourism, may be under-reported and therefore not fully represented in the dataset. Second, the ecological study design limits causal inference and does not capture subnational disparities or individual-level factors. Although our analysis focused on between-country differences, substantial within-country inequity—across socioeconomic or geographic groups—may also influence access to transplantation. Measures such as the GINI coefficient could provide further insight, but global reporting is inconsistent, and contemporaneous data are particularly sparse in many low- and middle-HDI settings. Third, estimated gaps in transplant rates were calculated relative to benchmark PMP values derived from very high-HDI countries. These benchmarks reflect normative system capacity rather than true clinical demand. Because the benchmark PMP does not incorporate country-specific epidemiology (e.g., hepatitis-related liver failure in Mongolia) or waiting-list pressure, the estimated transplant capacity gap likely represents a conservative approximation of the true deficit. In low-HDI settings, true demand may be substantially higher than benchmark-based estimates because limited access to preventative care, delayed diagnosis, and untreated chronic diseases increase the incidence of end-stage organ failure. More broadly, substantial shortfalls may persist even in countries that exceed benchmark thresholds, including the United States, and several high-income countries in Western Europe, where waiting-list mortality remains a concern.

Future research should focus on improving the quality and granularity of transplant data, including subnational analyses and linkages to clinical outcomes. Qualitative studies are also needed to better understand sociocultural and structural barriers —including sex and age-related disparities, racial inequities, health literacy, and geographic accessibility—that influence deceased organ donation across diverse settings. Moreover, because HDI does not capture within-country wealth inequality, incorporating measures such as the GINI coefficient could help characterize socioeconomic inequity in transplant access. Integrating transplant services within broader health system strengthening initiatives represents an essential direction for future policy and practice.

## Contributors

Z.K.C. conceived and designed the study, oversaw its administration, coordinated data collection and analysis, critically revised the manuscript, integrated contributions from co-authors, and led the scientific discussion. Z.K.C. takes responsibility for the decision to submit the manuscript for publication.

Q.X.H. conceived and designed the study, assisted with study administration, coordinated with collaborating investigators, contributed to scientific discussions, revised the manuscript, accessed and verified the underlying data.

H.P. contributed to the study design, data collection, data analysis, and interpretation of the results, prepared the manuscript, accessed and verified the underlying data.

H.Q. contributed to data collection, analysis, interpretation of the results, assisted in preparing the manuscript, and contributed to scientific discussion.

L.H.F. contributed to the study design, data collection, analysis, interpretation of the results, and manuscript preparation.

## Data sharing statement

The raw data are publicly available from the WHO Global Observatory on Donation and Transplantation (https://www.transplant-observatory.org). The harmonized and imputed dataset generated for this study has been deposited in Zenodo (https://doi.org/10.5281/zenodo.17215656). This record contains the standardized/imputed country–year data and the R code required to reproduce all figures and tables in the main text. The files are under embargo and will be made openly accessible upon publication. The full code for Appendix 1 and Appendix 2 (including extended and sensitivity analyses) is available from the corresponding author upon reasonable request for academic, non-commercial use.

## Editor note

The Lancet Group takes a neutral position with respect to territorial claims in published maps and institutional affiliations.

## Declaration of interests

We declare no competing interests.
