# Supplementary material for: Global inequities in organ transplantation, 2008–2023: trends, unmet need, and policy implications
Source: eClinicalMedicine. 2026 Feb 12;92:103788. doi: 10.1016/j.eclinm.2026.103788 (PMC12925128; doi:10.1016/j.eclinm.2026.103788)
Supplement: Supplementary Appendix 1 [file mmc1.pdf]

Supplementary Appendix 1

Global inequities in organ transplantation, 2008–2023: trends, unmet need, and policy implications

Table of contents

Section 1. Supplementary Background ... 6

1.1 GODT: data source and caveats ... 6

1.2 Human Development Index (HDI): definition and application ... 6

1.3 Japan: the 'Wada' case and subsequent legislation ... 6

1.4 Iran's state-regulated compensated living-unrelated kidney donation ... 6

1.5 China's 2015 policy shift on organ sources and national allocation ... 6

Section 2. Additional Methods ... 7

2.1 Data processing and definitions ... 7

2.2 Indicators and measures ... 7

2.3 Estimation of time trends (APC/AAPC) ... 8

2.4 Measurement of inequality (SII and CI) ... 8

2.5 Criteria for EAPC estimation ... 8

2.6 Coverage gap framework ... 9

2.7 Visualization and software ... 9

Section 3. Tables ... 10

Appendix 1 Table S1: GATHER checklist ... 10

Appendix 1 Table S2: Annual missingness (%) of organ-specific transplant data in the GODT database, 2008–2023 ... 11

Appendix 1 Table S3: Estimated population and proportional share of the global population by region and development level, 2008 and 2023 ... 12

Appendix 1 Table S4: Trends in the number and rate of kidney transplants by global region, 2008–2023, including percent change (PC) and estimated annual percent change (EAPC) with 95% confidence intervals ... 13

Appendix 1 Table S5: Trends in the number and rate of liver transplants by global region, 2008–2023, including percent change (PC) and estimated annual percent change (EAPC) with 95% confidence intervals ... 15

Appendix 1 Table S6: Trends in the number and rate of heart transplants by global region, 2008–2023, including percent change (PC) and estimated annual percent change (EAPC) with 95% confidence intervals ... 16

Appendix 1 Table S7: Trends in the number and rate of lung transplants by global region, 2008–2023, including percent change (PC) and estimated annual percent change (EAPC) with 95% confidence intervals 18

Appendix 1 Table S8: Trends in the number and rate of all pancreas transplants (SPK+PAK+PTA) by global region, 2008–2023, with percent change (PC) and estimated annual percent change (EAPC) with 95% CIs. 19

Appendix 1 Table S9: Trends in the number and rate of simultaneous pancreas–kidney (SPK) transplants by global region, 2008–2023, with percent change (PC) and estimated annual percent change (EAPC) with 95% CIs. 21

Appendix 1 Table S10: Trends in the number and rate of pancreas-only transplants (PTA+PAK) by global region, 2008–2023, with PC and EAPC (95% CIs). 22

Appendix 1 Table S11: Trends in the number and rate of small bowel transplants by global region, 2008–2023, including percent change (PC) and estimated annual percent change (EAPC) with 95% confidence intervals 24

Appendix 1 Table S12: Trends in the number and rate of total solid organ transplants by country, 2008–2023, including percent change (PC) and estimated annual percent change (EAPC) with 95% confidence intervals 25

Appendix 1 Table S13: Estimated national population and global population share by country, 2008 and 2023 29

Appendix 1 Table S14: Global Distribution of Solid Organ Transplantation Capacity and Corresponding Population Proportion, 2008–2010 36

Appendix 1 Table S15: Global Distribution of Solid Organ Transplantation Capacity and Corresponding Population Proportion, 2021–2023 41

Appendix 1 Table S16: Change in national transplant capacity for solid organs between 2008–2010 and 2021–2023, by organ type 48

Appendix 1 Table S17: Trends in kidney transplant number and rate by country, 2008–2023, and percent change (PC) and estimated annual percent change (EAPC, 95% CI) 54

Appendix 1 Table S18: Trends in the number and rate of liver transplants by country, 2008–2023, including percent change (PC) and estimated annual percent change (EAPC) with 95% confidence intervals 58

Appendix 1 Table S19: Trends in the number and rate of heart transplants by country, 2008–2023, including percent change (PC) and estimated annual percent change (EAPC) with 95% confidence intervals 61

Appendix 1 Table S20: Trends in the number and rate of lung transplants by country, 2008–2023, including percent change (PC) and estimated annual percent change (EAPC) with 95% confidence intervals 64

Appendix 1 Table S21: Trends in the number and rate of Pancreas transplants by country, 2008–2023, including percent change (PC) and estimated annual percent change (EAPC) with 95% confidence intervals 66

Appendix 1 Table S22: Trends in the number and rate of small bowel transplants by country, 2008–2023, including percent change (PC) and estimated annual percent change (EAPC) with 95% confidence intervals 68

Appendix 1 Table S23: Trends in Missing Kidney Transplants and Coverage Rates by Country, 2008–2023 69

Appendix 1 Table S24: Trends in Missing Liver Transplants and Coverage Rates by Country, 2008–2023 75

Appendix 1 Table S25: Trends in Missing Heart Transplants and Coverage Rates by Country, 2008–2023 81

Appendix 1 Table S26: Trends in Missing Lung Transplants and Coverage Rates by Country, 2008–2023 87

Appendix 1 Table S27: Trends in Missing Pancreas Transplants and Coverage Rates by Country, 2008–2023 92

Appendix 1 Table S28: Trends in Missing Small Bowel Transplants and Coverage Rates by Country, 2008–2023 98

Section 4. Figures 104

Appendix 1 Figure S1: Missingness of key transplant variables in the GODT dataset, 2008–2023 104

Appendix 1 Figure S2: Joinpoint regression analysis of global transplant rates, 2008–2023 105

Appendix 1 Figure S3: Joinpoint regression analysis of global transplant rates by HDI group, 2008–2023 106

Appendix 1 Figure S4: Joinpoint regression analysis of global transplant rates by WHO region, 2008–2023 107

Appendix 1 Figure S5: Kidney transplantation in Iran, 2008–2023—counts and donor-type composition 108

Appendix 1 Figure S6: Trends in organ transplant rate per million population (PMP) by HDI group and WHO region, 2008–2023 109

Appendix 1 Figure S7: Trends in solid organ transplantation PMP: Japan vs global and selected high-HDI countries, 2008–2023 110

Appendix 1 Figure S8: Organ-specific trends in transplantation in China, 2008–2023 111

Appendix 1 Figure S9: Contribution of HDI regions to global organ transplants, 2023 112

Appendix 1 Figure S10: Organ transplant PMP across GBD21 regions in 2008 and 2023 113

Appendix 1 Figure S11: National transplant trajectories and trends in relation to HDI, 2008–2023 114

Appendix 1 Figure S12: Distribution of solid organ transplants by HDI group, 2023 115

Section 5. Supplementary References 116

Section 1. Supplementary Background

1.1 GODT: data source and caveats

Country-level annual transplant counts were sourced from the GODT, a collaborative initiative of the World Health Organization (WHO) and the Spanish National Transplant Organization (Organización Nacional de Trasplantes, ONT). Peer-reviewed literature characterizes GODT as the most comprehensive global repository of donation and transplantation activity, comprising data derived from official national reports.[1,2] National statistics are submitted by competent authorities and compiled by ONT; figures are aggregated country reports rather than hospital-level audits. Coverage and definitions may vary by country, organ, and year, and datasets are periodically revised. All cross-country comparisons and trends should therefore be interpreted as reflecting reported activity rather than underlying clinical demand.[3] The analytic snapshot used in this study was downloaded on Apr 29, 2025, from the Global Observatory on Donation and Transplantation data portal (https://www.transplant-observatory.org/).

1.2 Human Development Index (HDI): definition and application

The Human Development Index (HDI) is a composite measure of national socioeconomic development introduced by the United Nations Development Program in 1990. It is calculated as the geometric mean of three key dimensions: life expectancy at birth, educational attainment (mean years of schooling and expected years of schooling), and gross national income per capita (purchasing power parity, constant international dollars). HDI values range from 0 to 1 and are used to classify countries into four development groups (low, medium, high, and very high).[4] Annual country-level HDI estimates from 2008–2022 were obtained from the UNDP Human Development Reports data portal (https://hdr.undp.org/data-center/human-development-index).

1.3 Japan: the 'Wada' case and subsequent legislation

Japan's first heart transplant in 1968 (the "Wada" case) provoked immediate controversy over brain-death determination and consent, triggered a police investigation without prosecution, and—at a time when brain death had no statutory definition—seeded public mistrust and legal uncertainty.5 Cadaveric donation effectively stalled for decades and practice shifted toward living donation.6 The Organ Transplant Law of 1997 cautiously recognized brain death for the purpose of donation but required the donor's premortem written consent plus family assent and excluded children, keeping early numbers very low. A 2009 amendment (effective 2010) allowed family consent in the absence of a recorded refusal and permitted paediatric donation, yielding only modest increases.7 Despite these reforms, deceased-donor activity has remained comparatively low, leaving Japan an outlier among very-high-HDI countries.

1.4 Iran's state-regulated compensated living-unrelated kidney donation

Iran introduced a state-regulated compensated living-unrelated donor (LURD) program for kidney transplantation in the late 1980s, under Ministry of Health oversight. The model formalized fixed, regulated compensation and prohibited brokerage, with transplants performed in accredited public/university centres;[8] over time additional safeguards were added to curb transplant tourism (e.g., requiring identical donor–recipient nationality and restricting foreign recipients). Proponents argue the program expanded access when deceased donation was nascent; critics highlight persistent concerns around equity, inducement, and long-term donor outcomes. A parallel policy track strengthened deceased donation: in 1989 a national religious fatwa recognized organ retrieval after brain death; in 2000 the Brain Death and Organ Transplantation Act legalized donation after brain death and created formal identification and procurement pathways. Since then, deceased-donor activity has risen markedly (e.g., national deceased-donor donation increased from very low baseline in the early 2000s to substantially higher levels by the late 2010s), although year-to-year volatility and COVID-19 disruptions have been reported. In recent years, multiple reports describe a shifting donor mix with a higher share of deceased-donor kidneys alongside the continuing LURD programme.[9]

1.5 China's 2015 policy shift on organ sources and national allocation

China's progress in organ transplantation is built upon a candid acknowledgment of its historical context and over two decades of challenging reform. This journey began with seven years of independent research and regional pilot initiatives (2003–2010),[10] leading to the formal launch of an internationally standardized deceased organ donation pilot program by the Ministry of Health and the Red Cross Society of China in 2010. Through five years of sustained effort, voluntary deceased citizen donation grew steadily to a level that could fully replace the traditional source. Consequently, national

authorities determined that conditions had matured to cease the utilization of organs from the prior source, and officially declared that, from January 1, 2015, deceased citizen donation would become the sole legal source for transplantation. This transition initiated a new, nationally coordinated phase in China's donation and transplantation system, guided by health administrative bodies and the Red Cross Society of China with the participation of all transplant hospitals under an internationally aligned framework. These substantive reforms, comprising both the government-led transition and the established voluntary donation system, are collectively recognized as the "China Model."[11-13]

Section 2. Additional Methods

2.1 Data processing and definitions

HDI is a composite indicator of life expectancy, education, and income, and countries were grouped into four development levels (very high, high, medium, and low) according to the UNDP Human Development Report.[4] WHO regions followed the official World Health Organization classification of six geographical regions. GBD21 regions were based on the 21-region framework of the Global Burden of Disease Study 2021, allowing consistent global comparisons.[14,15]

Country and territory names were harmonized to ensure consistency across data sources, with alignment to the GODT database for WHO region groupings and to the Global Burden of Disease 2021 (GBD21) regional classification. HDI categories were mapped according to the 2024 Human Development Report, with 2022 values applied to represent HDI groupings for 2023.

For transplant activity, missing values were handled using structured rules. The GODT reporting system underwent a structural transition in 2015. Before 2015, all WHO Member States were listed in the registry regardless of transplant activity; therefore, NA values in 2008–2014 largely reflected structural zeroes (countries without transplant activity) rather than missing data. From 2015 onward, GODT included only countries performing at least one transplant, and NA values represent true missingness.

To differentiate structural zeroes from true missingness, organ-specific transplant capability for 2008–2014 was defined as reporting ≥ 2 non-zero transplant observations for that organ during this period. Countries not meeting this criterion were considered not capable of performing that transplant type prior to 2015; NA values for these countries were therefore classified as structural zeroes rather than missing data.

Isolated missing values in countries with documented transplant activity were imputed by linear interpolation across adjacent years, whereas countries without evidence of activity or with sustained missing data were treated as zero or excluded, as appropriate. All interpolated values were manually reviewed prior to inclusion, in line with the Guidelines for Accurate and Transparent Health Estimates Reporting (GATHER), with documentation in the checklist (appendix 1 table S1).[16]

True missingness was defined as NA values occurring in countries with established transplant capability. Missingness rates for all seven transplant types (kidney, liver, heart, lung, pancreas, kidney–pancreas, and small bowel) were calculated annually after excluding structural zeroes. Summary statistics and heatmap visualizations of missingness are provided in Appendix Figure S1 and Appendix Table S2.

Population denominators were obtained from the GODT dataset. For countries with missing annual population counts, linear interpolation was applied between the nearest available years (e.g., if values were available for 2008 and 2021, interpolated estimates were used for 2009–2020). For years beyond the most recent reported population data (2022–2023), the last available estimate (typically 2021) was carried forward. This procedure ensured complete population coverage for all 194 countries from 2008–2023, while preserving consistency in denominators used to calculate PMP rates and coverage estimates.

2.2 Indicators and measures

We calculated transplant activity using the per-million population (PMP) rate for each organ, country, and year, defined as:

$$PMP = \left(\frac{Number\ of\ transplants}{Population}\right) \times 1000000$$

To assess relative change in transplant activity over time, we first calculated the percent change (PC) between 2008 and 2023 for both transplant counts and PMP rates using the formula:

$$PC = \left(\frac{Value_{2023} - Value_{2008}}{Value_{2008}}\right) \times 100\%$$

To evaluate long-term trends, we estimated the Estimated Annual Percent Change (EAPC) and its 95% confidence interval (CI) by fitting a linear regression model to the natural logarithm of the PMP values over time (t):

$$log(PMP_t) = \beta_0 + \beta_1 t$$

$$EAPC = (\exp(\beta_1) - 1) \times 100\%$$

The 95% CI for EAPC was calculated using the standard error of the slope coefficient β_1, assuming a normal distribution. A positive EAPC with a lower CI bound greater than zero was interpreted as a statistically significant increasing trend, and vice versa. To assess changes in transplant capacity, countries reporting at least one transplant for an organ in 2008–2010 were considered to have early capacity. Those with at least one transplant in 2021–2023 were considered to have maintained capacity; those with none in 2021–2023 despite prior activity were considered to have lost capacity.

2.3 Estimation of time trends (APC/AAPC)

For each solid organ, annual rates per million population (PMP) were modeled using an ordinary least-squares log-transformed linear regression of log(PMP + ε) on calendar year, where $\varepsilon = 1 \times 10^{-6}$ was added only when PMP equaled zero. This approach is mathematically equivalent to the 0-joinpoint log(PMP) model implemented in the NCI Joinpoint software, and allows estimation of a constant log-linear trend over time.

Segment-specific annual percent changes (APC) were derived as 100 × [exp(β)−1], with 95% confidence intervals obtained from the regression standard errors. The overall trend from 2008−2023 was summarized using the average annual percent change (AAPC), calculated as the duration-weighted mean of segment slopes following the Clegg method, with variance $\sum w_i^2 SE(\beta_i)^2$ and results back-transformed to percentages.

Segmented analyses with prespecified breakpoints at 2015 (major policy reform) and 2020 (COVID-19) were applied only to Appendix Figure S4; all other analyses used the 0-joinpoint formulation.

Breakpoints were prespecified a priori and not estimated from the data.

2.4 Measurement of inequality (SII and CI)

The SII quantifies absolute inequality in transplant activity across the HDI distribution. Countries were ranked from lowest to highest HDI, and a weighted linear regression of transplant activity (PMP) on the relative rank was fitted using population weights. The SII represents the absolute difference in transplant activity between the highest and lowest ends of the HDI spectrum. Positive values indicate higher activity in high-HDI countries.

The CI measures relative inequality and reflects how transplant activity is distributed across the HDI ranking. For each year (2008 and 2023), countries were ordered by HDI, and we computed (1) cumulative population proportions and (2) cumulative proportions of global transplant activity. The concentration curve was constructed and the CI derived using the standard covariance formula:

$$CI = \frac{2}{\mu} \mathrm{Cov}(y_i, R_i)$$

where y_i is the share of global transplant activity, R_i the fractional HDI rank, and μ the mean of y_i.

CI ranges from −1 to +1, with positive values indicating concentration of transplant activity in higher-HDI countries.

2.5 Criteria for EAPC estimation

2.5.1 Global, HDI, WHO region, and GBD21 super-region estimates

For global and regional analyses—including Human Development Index (HDI) groups, WHO regions, and GBD21 super-regions—the estimated annual percent change (EAPC) was derived using the NCI Joinpoint Regression Program (version 5.4.0). These aggregated series contain complete or near-complete annual observations with missingness, allowing stable log-linear modelling without

further data-adequacy restrictions. A 0-joinpoint model was applied to ensure comparability across all aggregated strata and to align with standard epidemiological practice for long-term global trend assessment. Joinpoint output plots for all aggregated analyses are presented in Appendix Figure S2-4.

2.5.2 Country-level EAPC estimation

Country-level transplant activity varies substantially in completeness and temporal continuity. To ensure that estimated trends reflected sustained programme activity rather than sporadic or unstable observations, EAPC estimation at the country–organ level followed strict inclusion criteria. A country–organ series was included only if all of the following conditions were satisfied:

(1) Evidence of maintained transplant capacity (2021–2023)

At least one non-zero transplant count during 2021–2023 was required to confirm ongoing transplant capability. Series with zero or missing values for all three years were excluded, as they likely reflected discontinued or intermittent programmes.

(2) Minimum number of informative observations

The series was required to have ≥4 non-zero annual observations between 2008–2023 to avoid unstable estimates driven by isolated activity.

(3) Adequate temporal span

The time interval between the earliest and latest non-zero observations had to be ≥6 years, ensuring sufficient longitudinal depth for log-linear estimation. Series failing to meet these requirements were excluded from country-level EAPC estimation and are indicated as "–" in the corresponding tables.

This capacity-informed framework mitigates bias from sporadic reporting and yields trend estimates that more reliably represent sustained transplant system performance.

2.6 Coverage gap framework

Transplant shortfalls (i.e., missing transplants) and coverage levels were quantified by comparing observed transplant volumes against expected volumes derived from a pre-specified transplants PMP. For each organ, the benchmark PMP was defined a priori as the weighted mean PMP of very-high-HDI countries, reflecting attainable rather than theoretical capacity. Expected transplant numbers for each country and year were calculated as the product of the benchmark PMP and the national population.

Coverage was defined as the ratio of observed to expected transplant numbers, with values greater than 1 truncated at 1 to indicate complete coverage. The absolute shortfall, or missing transplants, was defined as the difference between expected and observed numbers. Both coverage ratios and shortfalls were summarized globally and across HDI regions, and were displayed in organ-specific maps and bar charts. Benchmark PMP values for each organ are listed in appendix tables, and full results are presented in coverage gap maps and sensitivity analyses.

2.7 Visualization and software

Spatial and temporal patterns of transplant activity were displayed using multiple complementary approaches. Global choropleth maps were produced for PMP in 2008 and 2023, and for EAPC over the study period, with countries shaded according to standardized color scales. Countries without data were shown in grey and labelled as "no data." Regional bar charts were used to compare observed and expected transplant numbers by HDI group, WHO region, and GBD21 region. Heatmaps summarized organ-specific PMP values across the 21 GBD21 regions, and line plots illustrated PMP trajectories by HDI level. Frontier and trajectory plots were used to visualize country-level progress in transplant activity relative to socioeconomic development. To ensure comparability, all maps used a consistent projection and legend design, and color scales were harmonized across organs and time periods.

All figures and statistical analyses were generated using R (version 4.4.2; R Foundation for Statistical Computing, Vienna, Austria) and Python (version 3.9; Python Software Foundation). Key R packages included tidyverse for data processing, ggplot2 and cowplot for visualization, and segmented for trend estimation. Python was primarily used for supplementary data handling and figure formatting.

Section 3. Tables

Appendix 1 Table S1: GATHER checklist

Item #	Checklist item	Reporting location
**Objectives and funding**		
1	Define the indicator(s), populations (including age, sex, and geographic entities), and time period(s) for which estimates were made.	Main text (Methods); Appendix 1 (sections 2.1-2.5). (Note: GODT data do not provide age- or sex-specific disaggregation; all estimates refer to total populations.)
2	List the funding sources for the work.	Summary (funding)
**Data Inputs**		
For all data inputs from multiple sources that are synthesized as part of the study:		
3	Describe how the data were identified and how the data were accessed.	Main text (Methods); Appendix 1 (section 2.1)
4	Specify the inclusion and exclusion criteria. Identify all ad-hoc exclusions.	Appendix 1 (section 2.5)
5	Provide information on all included data sources and their main characteristics. For each data source used, report reference information or contact name/institution, population represented, data collection method, year(s) of data collection, sex and age range, diagnostic criteria or measurement method, and sample size, as relevant.	Main text (Methods); Appendix 1 (sections 1.1-1.2, section 2.1)
6	Identify and describe any categories of input data that have potentially important biases (e.g., based on characteristics listed in item 5).	Main text (Methods); Appendix 1 (section 2.1); Appendix 2 (sections 1-3)
For data inputs that contribute to the analysis but were not synthesized as part of the study:		
7	Describe and give sources for any other data inputs.	Online data citation tool: (https://ghdx.healthdata.org/gbd-2021)
For all data inputs:		
8	Provide all data inputs in a file format from which data can be efficiently extracted (e.g., a spreadsheet rather than a PDF), including all relevant meta-data listed in item 5. For any data inputs that cannot be shared because of ethical or legal reasons, such as third-party ownership, provide a contact name or the name of the institution that retains the right to the data.	Online data citation tool: (https://www.transplant-observatory.org/); Main text (Data sharing); Restricted data: none,
**Data analysis**		
9	Provide a conceptual overview of the data analysis method. A diagram may be helpful.	Main text (Methods); Appendix 1 (sections 2.1-2.7)

10	Provide a detailed description of all steps of the analysis, including mathematical formulae. This description should cover, as relevant, data cleaning, data pre-processing, data adjustments and weighting of data sources, and mathematical or statistical model(s).	Main text (Methods); Appendix 1 (sections 2.1-2.7)
11	Describe how candidate models were evaluated and how the final model(s) were selected.	Main text (Methods); Appendix 1 (section 2.1)
12	Provide the results of an evaluation of model performance, if done, as well as the results of any relevant sensitivity analysis.	Appendix 2 (sections 2-3)
13	Describe methods for calculating uncertainty of the estimates. State which sources of uncertainty were, and were not, accounted for in the uncertainty analysis.	Main text (Methods); Appendix 1 (sections 2.1-2.7)
14	State how analytic or statistical source code used to generate estimates can be accessed.	Main text (Data sharing)
**Results and Discussion**		
15	Provide published estimates in a file format from which data can be efficiently extracted.	Main text (Table 1); Appendix 1 (Tables S1–S28); Supplementary datasets (CSV/Excel)
16	Report a quantitative measure of the uncertainty of the estimates (e.g. uncertainty intervals).	Main text (Methods); Appendix 1 (sections 2.1-2.6)
17	Interpret results in light of existing evidence. If updating a previous set of estimates, describe the reasons for changes in estimates.	Main text (Discussion, Comparison with prior evidence)
18	Discuss limitations of the estimates. Include a discussion of any modelling assumptions or data limitations that affect interpretation of the estimates.	Main text (Discussion, Limitations)

Appendix 1 Table S2: Annual missingness (%) of organ-specific transplant data in the GODT database, 2008–2023

Year	Kidney	Liver	Heart	Lung	Pancreas-only	Kidney–pancreas	Small bowel
2008	18·9	20·3	21·3	20·5	23·9	22·7	39·1
2009	13·2	10·8	9·8	13·6	97·8	13·6	30·4
2010	10·4	10·8	8·2	0·0	100·0	13·6	26·1
2011	6·6	6·8	4·9	4·5	2·2	2·3	17·4
2012	6·6	4·1	3·3	2·3	2·2	2·3	17·4
2013	0·9	0·0	0·0	0·0	0·0	4·5	8·7
2014	5·7	5·4	0·0	4·5	2·2	2·3	4·3
2015	1·8	3·6	7·1	9·8	10·7	10·7	11·6

2016	0·0	1·2	1·2	1·2	2·4	3·7	3·7
2017	0·0	1·2	1·2	1·2	2·4	6·0	6·0
2018	1·1	2·2	2·2	4·4	6·6	28·6	9·9
2019	2·3	3·5	4·7	8·1	9·3	11·6	14·0
2020	1·1	2·1	4·3	5·3	6·4	8·5	10·6
2021	1·1	4·4	6·6	7·7	7·7	7·7	18·7
2022	0·0	2·2	5·4	5·4	4·3	5·4	15·2
2023	0·0	1·1	2·2	2·2	3·2	4·3	8·6

Missingness was calculated for countries with established transplant activity; structural zeroes were excluded.

Appendix 1 Table S3: Estimated population and proportional share of the global population by region and development level, 2008 and 2023

	**2008**		**2023**	
**Region classification**	**Population(millions)**	**Population share(%)**	**Population(millions)**	**Population share(%)**
**Global**	6741·4	100·00	7739·5	100·00
**Human Development Index**				
Very High	1125·2	16·69	1644·5	21·25
High	1055·6	15·66	2836·3	36·65
Medium	3313·3	49·15	2148·9	27·77
Low	1247·3	18·50	1109·8	14·34
**World Health Organization Region**				
Africa	811·3	12·03	1047·6	13·54
America	915·7	13·58	1036·7	13·39
Eastern Mediterranean	572·7	8·50	744·9	9·62
Europe	891·1	13·22	923·8	11·94
South-East Asia	1769·6	26·25	2057·7	26·59
Western Pacific	1781·1	26·42	1928·9	24·92
**Global Burden of Disease Region**				
High-income Asia Pacific	180·8	2·68	181·1	2·34
High-income North America	342·0	5·07	378·8	4·89
Western Europe	404·2	6·00	424·2	5·48
Australasia	25·2	0·37	31·6	0·41
Andean Latin America	98·1	1·46	117·1	1·51
Tropical Latin America	194·2	2·88	216·4	2·80

Central Latin America	155·8	2·31	186·8	2·41
Southern Latin America	60·1	0·89	68·8	0·89
Caribbean	65·5	0·97	68·8	0·89
Central Europe	130·4	1·93	126·5	1·63
Eastern Europe	197·3	2·93	192·1	2·48
Central Asia	76·4	1·13	86·0	1·11
North Africa and Middle East	475·0	7·05	578·6	7·48
South Asia	1591·9	23·61	1935·9	25·01
Southeast Asia	575·4	8·54	647·9	8·37
East Asia	1362·9	20·22	1462·3	18·89
Oceania	9·7	0·14	10·5	0·14
Western Sub-Saharan Africa	293·3	4·35	390·9	5·05
Eastern Sub-Saharan Africa	325·1	4·82	430·4	5·56
Central Sub-Saharan Africa	122·3	1·81	139·8	1·81
Southern Sub-Saharan Africa	55·9	0·83	65·1	0·84

Note: Population values are presented in millions (M). Share indicates the proportion of global total in each respective year.

Appendix 1 Table S4: Trends in the number and rate of kidney transplants by global region, 2008–2023, including percent change (PC) and estimated annual percent change (EAPC) with 95% confidence intervals

	**2008**		**2023**		**2008-2023**	
**Region classification**	**Kidney transplants**	**PMP**	**Kidney transplants**	**PMP**	**PC(%)**	**EAPC (%, 95% CI)**
**Global**	70213	10·4	115702	14·9	64·79	1·92 (1·21 to 2·64)
**Human Development Index**						
Very High	39756	35·3	68696	41·8	72·79	0·84 (0·31 to 1·37)
High	13986	13·2	30250	10·7	116·29	-2·66 (-4·35 to -0·94)
Medium	15399	4·6	14499	6·7	-5·84	2·34 (0·21 to 4·51)
Low	1072	0·9	2257	2.0	110·54	4·36 (-0·11 to 9·02)
**World Health Organization Region**						
Africa	505	0·6	643	0·6	27·33	1·48 (-0·01 to 2·99)
America	26726	29·2	43097	41·6	61·25	1·88 (1·35 to 2·41)
Eastern Mediterranean	5226	9·1	7171	9·6	37·22	-2·10 (-4·40 to 0·26)
Europe	20461	23·0	28431	30·8	38·95	1·07 (0·25 to 1·90)

South-East Asia	6819	3·9	14982	7·3	119·71	4·30 (2·42 to 6·20)
Western Pacific	10476	5·9	21378	11·1	104·07	5·17 (3·99 to 6·36)
**Global Burden of Disease Region**						
High-income Asia Pacific	2279	12·6	3950	21·8	73·32	3·46 (2·51 to 4·42)
High-income North America	17721	51·8	30222	79·8	70·54	3·10 (2·61 to 3·60)
Western Europe	15226	37·7	19598	46·2	28·71	0·64 (-0·06 to 1·36)
Australasia	897	35·6	1261	39·9	40·58	0·71 (-0·31 to 1·73)
Andean Latin America	842	8·6	1380	11·8	63·90	-0·70 (-3·04 to 1·69)
Tropical Latin America	3780	19·5	5811	26·9	53·73	0·66 (-0·61 to 1·95)
Central Latin America	2559	16·4	3389	18·1	32·43	-2·03 (-5·12 to 1·16)
Southern Latin America	1287	21·4	2101	30·5	63·25	1·19 (-0·35 to 2·76)
Caribbean	537	8·2	194	2·8	-63·87	-10·94 (-14·46 to -7·27)
Central Europe	2387	18·3	2747	21·7	15·08	-0·37 (-1·59 to 0·87)
Eastern Europe	821	4·2	1875	9·8	128·38	4·80 (3·31 to 6·31)
Central Asia	223	2·9	308	3·6	38·12	0·34 (-1·76 to 2·49)
North Africa and Middle East	6367	13·4	9382	16·2	47·35	-0·54 (-2·07 to 1·01)
South Asia	6724	4·2	15780	8·2	134·68	4·48 (2·25 to 6·76)
Southeast Asia	2066	3·6	2144	3·3	3·78	-0·90 (-2.92 to 1·17)
East Asia	6104	4·5	15008	10·3	145·87	6·95 (5·09 to 8·84)
Oceania	0	0·0	0	0·0	–	–
Western Sub-Saharan Africa	15	0·1	253	0·6	1586·67	18·04 (13·90 to 22·32)
Eastern Sub-Saharan Africa	128	0·4	69	0·2	-46·09	3·48 (-2·12 to 9·40)
Central Sub-Saharan Africa	0	0·0	0	0·0	–	–
Southern Sub-Saharan Africa	250	4·5	230	3·5	-8·00	-2·30 (-3·30 to -1·29)

Abbreviations: PMP = per million population; PC = percent change; EAPC = estimated annual percent change; CI = confidence interval

Notes: 1.EAPC and 95% CIs were calculated by fitting a linear regression model to the natural logarithm of transplant rates (log[PMP]) across years (log[PMP] ~ Year). 2.A positive EAPC with a 95% CI not crossing zero indicates a statistically significant increasing trend; a negative EAPC with a 95% CI not crossing zero indicates a decreasing trend. 3."–" indicates that PC could not be estimated due to a zero baseline (i.e., 2008 = 0) or no transplants in both 2008 and 2023.

Appendix 1 Table S5: Trends in the number and rate of liver transplants by global region, 2008–2023, including percent change (PC) and estimated annual percent change (EAPC) with 95% confidence intervals

	2008		2023		2008-2023	
**Region classification**	**Liver transplants**	**PMP**	**Liver transplants**	**PMP**	**PC(%)**	**EAPC (%, 95% CI)**
**Global**	20440	3·0	42682	5·5	108·82	3.90 (3·38 to 4·42)
**Human Development Index**						
Very High	15574	13·8	26501	16·1	70·16	0·95 (0·41 to 1·49)
High	2516	2·4	11164	3·9	343·72	3·07 (1·82 to 4·34)
Medium	2349	0·7	4491	2·1	91·19	7·08 (4.73 to 9·49)
Low	1	<0·1	526	0·5	52500·00	64·48 (42·12 to 90·34)
**World Health Organization Region**						
Africa	23	<0·1	90	0·1	291·30	6·86 (4·86 to 8·91)
America	8638	9·4	14985	14·5	73·48	2·81 (2·41 to 3·21)
Eastern Mediterranean	389	0·7	2060	2·8	429·56	7·63 (3·96 to 11·43)
Europe	7564	8·4	11503	12·5	52·08	2·10 (1·50 to 2·71)
South-East Asia	303	0·2	4668	2·3	1440·59	16·29 (13·30 to 19·37)
Western Pacific	3523	2·0	9376	4·9	166·14	7·02 (5·65 to 8·39)
**Global Burden of Disease Region**						
High-income Asia Pacific	1303	7·2	1997	11·0	53·26	2·26 (1·64 to 2·88)
High-income North America	6863	20·1	11329	29·9	65·07	2·98 (2·45 to 3·50)
Western Europe	6250	15·5	7633	18·0	22·13	0·61 (0·13 to 1·08)
Australasia	219	8·7	342	10·8	56·16	1·57 (0·49 to 2·65)
Andean Latin America	212	2·2	385	3·3	81·60	1·13 (-0·29 to 2·56)
Tropical Latin America	1053	5·4	2284	10·6	116·90	3·58 (2·48 to 4·70)
Central Latin America	112	0·7	327	1·8	191·96	4·96 (1·55 to 8·48)
Southern Latin America	357	5·9	656	9·5	83·75	2·77 (1·59 to 3·96)
Caribbean	41	0·6	4	0·1	-90·24	-16·14 (-20·22 to -11·86)
Central Europe	554	4·2	1136	9·0	105·05	3·59 (1·99 to 5·22)
Eastern Europe	56	0·3	768	4·0	1271·43	16·18 (11·19 to 21·40)

Central Asia	46	0·6	95	1·1	106·52	4·01 (1·27 to 6·81)
North Africa and Middle East	1050	2·2	3407	5·9	224·48	5·74 (3·37 to 8·15)
South Asia	250	0·2	5038	2·6	1915·20	17·95 (14·36 to 21·67)
Southeast Asia	64	0·1	196	0·3	206·25	5·47 (4·04 to 6·91)
East Asia	1990	1·5	6997	4·8	251·61	10·09 (7·85 to 12·36)
Oceania	0	0·0	0	0·0	–	–
Western Sub-Saharan Africa	0	0·0	0	0·0	–	–
Eastern Sub-Saharan Africa	0	0·0	0	0·0	–	–
Central Sub-Saharan Africa	0	0·0	0	0·0	–	–
Southern Sub-Saharan Africa	20	0·4	88	1·4	340·00	8·37 (6·25 to 10·54)

Abbreviations: PMP = per million population; PC = percent change; EAPC = estimated annual percent change; CI = confidence interval.
Notes:1.EAPC and 95% CIs were calculated by fitting a linear regression model to the natural logarithm of transplant rates (log[PMP]) across years (log[PMP] ~ Year). 2.A positive EAPC with a 95% CI not crossing zero indicates a statistically significant increasing trend; a negative EAPC with a 95% CI not crossing zero indicates a decreasing trend. 3.“–” indicates that PC could not be estimated due to a zero baseline (i.e., 2008 = 0) or no transplants in both 2008 and 2023.

Appendix 1 Table S6: Trends in the number and rate of heart transplants by global region, 2008–2023, including percent change (PC) and estimated annual percent change (EAPC) with 95% confidence intervals

	**2008**		**2023**		**2008-2023**	
**Region classification**	**Heart transplants**	**PMP**	**Heart transplants**	**PMP**	**PC(%)**	**EAPC (%, 95% CI)**
**Global**	5359	0·8	10548	1·4	96·83	3·56 (3·08 to 4·05)
**Human Development Index**						
Very High	4726	4·2	8479	5·2	79·41	1·30 (0·72 to 1·89)
High	472	0·4	1848	0·7	291·53	2·33 (0·08 to 4·62)
Medium	161	<0·1	221	0·1	37·27	9·43 (2·52 to 16·80)
Low	0	0·0	0	0·0	–	–
**World Health Organization Region**						
Africa	25	<0·1	42	<0·1	68·00	2·81 (0·17 to 5·53)
America	2766	3·0	5544	5·3	100·43	3·85 (3·50 to 4·21)
Eastern Mediterranean	71	0·1	201	0·3	183·10	5·26 (3.10 to 7·45)

Europe	2198	2·5	3002	3·2	36·58	1·47 (0·97 to 1·97)
South-East Asia	10	<0·1	253	0·1	2430·00	24·88 (17·96 to 32·19)
Western Pacific	289	0·2	1506	0·8	421·11	11·67 (10·28 to 13·08)
**Global Burden of Disease Region**						
High-income Asia Pacific	64	0·4	365	2·0	470·31	10·28 (8·18 to 12·42)
High-income North America	2331	6·8	4783	12·6	105·19	4·28 (3·92 to 4·65)
Western Europe	1892	4·7	2125	5·0	12·32	0·15 (-0·32 to 0·63)
Australasia	95	3·8	145	4·6	52·63	3·32 (1·91 to 4·74)
Andean Latin America	84	0·9	129	1·1	53·57	0·33 (-1·91 to 2·62)
Tropical Latin America	200	1·0	429	2·0	114·5	4·95 (3·10 to 6·84)
Central Latin America	18	0·1	49	0·3	172·22	2·70 (-1·44 to 7·00)
Southern Latin America	130	2·2	154	2·2	18·46	0·91 (-0·43 to 2·28)
Caribbean	3	0·0	0	0·0	-100·00	-39.80 (-49·62 to -28·07)
Central Europe	212	1·6	441	3·5	108·02	4·40 (3·61 to 5·19)
Eastern Europe	21	0·1	358	1·9	1604·76	17·30 (10·91 to 24·05)
Central Asia	2	<0·1	5	0·1	150·00	5·59 (-3·59 to 15·64)
North Africa and Middle East	142	0·3	274	0·5	92·96	2·15 (0·00 to 4·35)
South Asia	5	<0·1	222	0·1	4340·00	29·89 (21·00 to 39·43)
Southeast Asia	5	<0·1	33	0·1	560·00	11·91 (8·25 to 15·70)
East Asia	130	0·1	994	0·7	664·62	15·61 (12·92 to 18·36)
Oceania	0	0·0	0	0·0	–	–
Western Sub-Saharan Africa	0	0·0	0	0·0	–	–
Eastern Sub-Saharan Africa	0	0·0	0	0·0	–	–
Central Sub-Saharan Africa	0	0·0	0	0·0	–	–
Southern Sub-Saharan Africa	25	0·4	42	0·6	68·00	3·50 (0·88 to 6·18)

Abbreviations: PMP = per million population; PC = percent change; EAPC = estimated annual percent change; CI = confidence interval.
Notes: 1.EAPC and 95% CIs were calculated by fitting a linear regression model to the natural logarithm of transplant rates (log[PMP]) across years (log[PMP] ~ Year). 2.A positive EAPC with a 95% CI not crossing zero indicates a statistically significant increasing trend; a negative EAPC with a 95% CI not crossing zero indicates a decreasing trend. 3."–" indicates that PC could not be estimated due to a zero baseline (i.e., 2008 = 0) or no transplants in both 2008 and 2023.

Appendix 1 Table S7: Trends in the number and rate of lung transplants by global region, 2008–2023, including percent change (PC) and estimated annual percent change (EAPC) with 95% confidence intervals

	2008		2023		2008-2023	
**Region classification**	**Lung transplants**	**PMP**	**Lung transplants**	**PMP**	**PC(%)**	**EAPC (%, 95% CI)**
**Global**	3329	0·5	7855	1·0	135·96	4·29 (3·64 to 4·95)
**Human Development Index**						
Very High	3215	2·9	6536	4·0	103·30	1·48 (0·94 to 2·03)
High	74	0·1	1122	0·4	1416·22	14·89 (11·94 to 17·92)
Medium	40	<0·1	197	0·1	392·50	22·07 (12·51 to 32·45)
Low	0	0·0	0	0·0	–	–
**World Health Organization Region**						
Africa	5	<0·1	23	<0·1	360·00	11·48 (8·02 to 15·05)
America	1726	1·9	3717	3·6	115·35	3·85 (3·24 to 4·47)
Eastern Mediterranean	1	<0·1	70	0·1	6900·00	13.10 (4·05 to 22·94)
Europe	1411	1·6	2366	2·6	67·68	2·17 (1·18 to 3·17)
South-East Asia	0	0·0	201	0·1	–	69·06 (46·42 to 95·19)
Western Pacific	186	0·1	1478	0·8	694·62	15·52 (14·30 to 16·76)
**Global Burden of Disease Region**						
High-income Asia Pacific	22	0·1	330	1·8	1400·00	19·49 (15·38 to 23·75)
High-income North America	1613	4·7	3530	9·3	118·85	4·15 (3·52 to 4·78)
Western Europe	1326	3·3	2070	4·9	56·11	1·47 (0·46 to 2·49)
Australasia	129	5·1	189	6.0	46·51	1·03 (-0·70 to 2·78)
Andean Latin America	14	0·1	37	0·3	164·29	8·43 (4·60 to 12·40)
Tropical Latin America	53	0·3	81	0·4	52·83	4·15 (0·52 to 7·92)
Central Latin America	1	<0·1	12	0·1	1100·00	35·56 (17·14 to 56·87)
Southern Latin America	45	0·7	57	0·8	26·67	0·94 (-1·25 to 3·17)
Caribbean	0	0·0	0	0·0	–	–

Central Europe	33	0·3	197	1·6	496·97	14·99 (13·08 to 16·92)
Eastern Europe	0	0·0	23	0·1	–	45·80 (22·08 to 74·14)
Central Asia	0	0·0	1	<0·1	–	46·21 (13·25 to 88·78)
North Africa and Middle East	53	0·1	145	0·3	173·58	3·52 (0·94 to 6·16)
South Asia	0	0·0	198	0·1	–	74·01 (49·24 to 102·88)
Southeast Asia	0	0·0	3	<0·1	–	5·40 (-13·18 to 27·95)
East Asia	35	<0·1	959	0·7	2640·00	31·11 (24·82 to 37·73)
Oceania	0	0·0	0	0·0	–	–
Western Sub-Saharan Africa	0	0·0	0	0·0	–	–
Eastern Sub-Saharan Africa	0	0·0	0	0·0	–	–
Central Sub-Saharan Africa	0	0·0	0	0·0	–	–
Southern Sub-Saharan Africa	5	0·1	23	0·4	360·00	12·22 (8·79 to 15·76)

Abbreviations: PMP = per million population; PC = percent change; EAPC = estimated annual percent change; CI = confidence interval.
Notes:1.EAPC and 95% CIs were calculated by fitting a linear regression model to the natural logarithm of transplant rates (log[PMP]) across years (log[PMP] ~ Year). 2.A positive EAPC with a 95% CI not crossing zero indicates a statistically significant increasing trend; a negative EAPC with a 95% CI not crossing zero indicates a decreasing trend. 3."–" indicates that PC could not be estimated due to a zero baseline (i.e., 2008 = 0) or no transplants in both 2008 and 2023.

Appendix 1 Table S8: Trends in the number and rate of all pancreas transplants (SPK+PAK+PTA) by global region, 2008–2023, with percent change (PC) and estimated annual percent change (EAPC) with 95% CIs.

	**2008**		**2023**		**2008-2023**	
**Region classification**	**All pancreas transplants**	**PMP**	**All pancreas transplants**	**PMP**	**PC(%)**	**EAPC (%, 95% CI)**
**Global**	2381	0·4	2117	0·3	-11·09	12·01 (-2·24 to 28·34)
**Human Development Index**						
Very High	2304	2·0	1831	1·1	-20·53	18·95 (-7.60 to 53·13)
High	72	0·1	259	0·1	259·72	5·98 (-0·10 to 12·44)
Medium	5	<0·1	27	<0·1	440·00	28·84 (15·13 to 44·18)
Low	0	0·0	0	0·0	–	–
**World Health Organization Region**						

Africa	5	<0·1	8	<0·1	60.00	15.37 (-0·45 to 33·69)
America	1488	1·6	1149	1·1	-22·78	37·40 (0·25 to 88·30)
Eastern Mediterranean	15	<0·1	61	0·1	306·67	29·66 (6·64 to 57·66)
Europe	809	0·9	680	0·7	-15·95	7·30 (-3·56 to 19·39)
South-East Asia	0	0·0	30	<0·1	–	42·52 (30·22 to 55·99)
Western Pacific	64	<0·1	189	0·1	195·31	29·73 (5·62 to 59·36)
**Global Burden of Disease Region**						
High-income Asia Pacific	28	0·2	62	0·3	121·43	36·46 (4·32 to 78·51)
High-income North America	1358	4·0	980	2·6	-27·84	41·45 (0·45 to 99·17)
Western Europe	715	1·8	575	1·4	-19·58	17·87 (-8·57 to 51·96)
Australasia	36	1·4	50	1·6	38·89	42·24 (4·05 to 94·43)
Andean Latin America	5	0·1	15	0·1	200·00	32·24 (7·56 to 62·59)
Tropical Latin America	32	0·2	119	0·5	271·88	39·50 (5·27 to 84·88)
Central Latin America	1	<0·1	0	0·0	-100·00	1·31 (-21.36 to 30·51)
Southern Latin America	91	1·5	35	0·5	-61·54	32·11 (-3·74 to 81·30)
Caribbean	1	<0·1	0	0·0	-100·00	-2·28 (-21·64 to 21·86)
Central Europe	70	0·5	80	0·6	14·29	34·83 (0·77 to 80·40)
Eastern Europe	2	<0·1	18	0·1	800·00	12·69 (4·56 to 21·44)
Central Asia	1	<0·1	0	0·0	-100·00	-32·03 (-42·86 to -19·16)
North Africa and Middle East	36	0·1	68	0·1	88·89	5·09 (0·13 to 10·29)
South Asia	0	0·0	27	<0·1	–	50·37 (35·29 to 67·12)
Southeast Asia	0	0·0	4	<0·1	–	30·15 (14·33 to 48·16)
East Asia	0	0·0	76	0·1	–	28·04 (6·50 to 53·93)
Oceania	0	0·0	0	0·0	–	–
Western Sub-Saharan Africa	0	0·0	0	0·0	–	–
Eastern Sub-Saharan Africa	0	0·0	0	0·0	–	–
Central Sub-Saharan Africa	0	0·0	0	0·0	–	–
Southern Sub-Saharan Africa	5	0·1	8	0·1	60·00	27·60 (1·10 to 61·06)

Abbreviations: PMP = per million population; PC = percent change; EAPC = estimated annual percent change; CI = confidence interval; SPK = simultaneous pancreas–kidney; PAK = pancreas after kidney; PTA = pancreas transplant alone.
Notes:1.EAPC and 95% CIs were calculated by fitting a linear regression model to the natural logarithm of transplant rates (log[PMP]) across years (log[PMP] ~ Year). 2.A positive EAPC with a 95% CI not crossing zero indicates a statistically significant increasing trend; a negative EAPC with a 95% CI not crossing zero indicates a decreasing trend. 3."–" indicates that PC could not be estimated due to a zero baseline (i.e., 2008 = 0) or no transplants in both 2008 and 2023.

Appendix 1 Table S9: Trends in the number and rate of simultaneous pancreas–kidney (SPK) transplants by global region, 2008–2023, with percent change (PC) and estimated annual percent change (EAPC) with 95% CIs.

	**2008**		**2023**		**2008-2023**	
**Region classification**	**Simultaneous pancreas–kidney transplants**	**PMP**	**Simultaneous pancreas–kidney transplants**	**PMP**	**PC(%)**	**EAPC (%, 95% CI)**
**Global**	1764	0·3	1830	0·2	3·74	-1·00 (-1·48 to -0·52)
**Human Development Index**						
Very High	1707	1·5	1587	1·0	-7·03	-3·07 (-3·62 to -2·50)
High	52	<0·1	220	<0·1	323·08	1·20 (-3·11 to 5·69)
Medium	5	<0·1	23	<0·1	360·00	13·85 (1·04 to 28·28)
Low	0	0·0	0	0·0	–	–
**World Health Organization Region**						
Africa	5	<0·1	8	<0·1	60·00	-2·61 (-10·40 to 5·85)
America	1024	1·1	999	1·0	-2·44	-0·64 (-1·23 to -0·04)
Eastern Mediterranean	2	<0·1	47	<0·1	2250·00	13·70 (3·12 to 25·36)
Europe	682	0·8	601	0·7	-11·88	-2·09 (-2·92 to -1·26)
South-East Asia	0	0·0	26	<0·1	–	42·56 (26·76 to 60·32)
Western Pacific	51	<0·1	149	<0·1	192·16	4·32 (2·18 to 6·50)
**Global Burden of Disease Region**						
High-income Asia Pacific	15	<0·1	36	0·2	140·00	3·54 (-0·53 to 7·78)
High-income North America	901	2·6	859	2·3	-4·66	-0·50 (-1·24 to 0·24)
Western Europe	599	1·5	506	1·2	-15·53	-2·32 (-3·08 to -1·56)
Australasia	36	1·4	44	1·4	22·22	0·63 (-0·94 to 2·22)
Andean Latin America	6	<0·1	15	0·1	150·00	3·38 (-1·63 to 8·66)

Tropical Latin America	32	0·2	93	0·4	190·62	20·03 (-3·24 to 48·90)
Central Latin America	1	<0·1	0	0·0	-100·00	-28·12 (-38·93 to -15·41)
Southern Latin America	83	1·4	32	0·5	-61·45	-5·17 (-7·31 to -2·98)
Caribbean	1	<0·1	0	0·0	-100·00	-4·39 (-20·68 to 15·25)
Central Europe	67	0·5	71	0·6	5·97	-0·69 (-2·75 to 1·41)
Eastern Europe	2	<0·1	18	<0·1	800·00	9·94 (0·29 to 20·52)
Central Asia	0	0·0	0	0·0	–	0·70 (-11·69 to 14·83)
North Africa and Middle East	16	<0·1	53	<0·1	231·25	4·63 (-1·88 to 11·58)
South Asia	0	0·0	23	<0·1	–	50·57 (35·95 to 66.76)
Southeast Asia	0	0·0	4	<0·1	–	28·28 (12·39 to 46·42)
East Asia	0	0·0	68	<0·1	–	27·73 (6·43 to 53·28)
Oceania	0	0·0	0	0·0	–	–
Western Sub-Saharan Africa	0	0·0	0	0·0	–	–
Eastern Sub-Saharan Africa	0	0·0	0	0·0	–	–
Central Sub-Saharan Africa	0	0·0	0	0·0	–	–
Southern Sub-Saharan Africa	5	<0·1	8	0·1	60·00	-1·96 (-9·78 to 6·53)

Abbreviations: PMP = per million population; PC = percent change; EAPC = estimated annual percent change; CI = confidence interval.
Notes:1.EAPC and 95% CIs were calculated by fitting a linear regression model to the natural logarithm of transplant rates (log[PMP]) across years (log[PMP] ~ Year). 2.A positive EAPC with a 95% CI not crossing zero indicates a statistically significant increasing trend; a negative EAPC with a 95% CI not crossing zero indicates a decreasing trend. 3."–" indicates that PC could not be estimated due to a zero baseline (i.e., 2008 = 0) or no transplants in both 2008 and 2023.

Appendix 1 Table S10: Trends in the number and rate of pancreas-only transplants (PTA+PAK) by global region, 2008–2023, with PC and EAPC (95% CIs).

	2008		2023		2008-2023	
**Region classification**	**Pancreas-only transplants**	**PMP**	**Pancreas-only transplants**	**PMP**	**PC(%)**	**EAPC (%, 95% CI)**
**Global**	618	<0·1	287	<0·1	-53·56	5·99 (-6·34 to 19·94)
**Human Development Index**						
Very High	597	0·5	244	0·1	-59·13	12·72 (-10·09 to 41·33)
High	21	<0·1	39	<0·1	85·71	-0·77 (-4.94 to 3.58)
Medium	0	0·0	4	<0·1	–	21·66 (6·56 to 38·90)

Low	0	0·0	0	0·0	–	–
**World Health Organization Region**						
Africa	0	0·0	0	0·0	–	-7·26 (-21·86 to 10·07)
America	465	0·5	150	0·1	-67·74	23·90 (-6·11 to 63·50)
Eastern Mediterranean	13	<0·1	14	<0·1	7·69	20·07 (-0·54 to 44·96)
Europe	127	0·1	79	<0·1	-37·80	1·73 (-6·82 to 11·06)
South-East Asia	0	0·0	4	<0·1	–	27·90 (12·43 to 45·49)
Western Pacific	13	<0·1	40	<0·1	207·69	24·38 (5·03 to 47·28)
**Global Burden of Disease Region**						
High-income Asia Pacific	13	<0·1	26	0·1	100·00	33·06 (4·18 to 69·96)
High-income North America	457	1·3	121	0·3	-73·52	26·50 (-6.96 to 71·99)
Western Europe	116	0·3	69	0·2	-40·52	12·52 (-9·60 to 40·04)
Australasia	0	0·0	6	0·2	–	87·43 (53·85 to 128·34)
Andean Latin America	0	0·0	0	0·0	–	6·32 (-17·96 to 37·78)
Tropical Latin America	0	0·0	26	0·1	–	50·49 (17·15 to 93·32)
Central Latin America	0	0·0	0	0·0	–	16·18 (-7·82 to 46·43)
Southern Latin America	8	0·1	3	<0·1	-62·50	20·49 (-5·10 to 52.98)
Caribbean	0	0·0	0	0·0	–	2·20 (-10·73 to 17·01)
Central Europe	3	<0·1	9	<0·1	200·00	28·10 (2·76 to 59·69)
Eastern Europe	0	0·0	0	0·0	–	-7·07 (-30·44 to 24·14)
Central Asia	1	<0·1	0	0·0	-100·00	-32·51 (-42·02 to -21·44)
North Africa and Middle East	20	<0·1	15	<0·1	-25·00	-2·10 (-7·54 to 3·67)
South Asia	0	0·0	4	<0·1	–	26·65 (10·30 to 45·41)
Southeast Asia	0	0·0	0	0·0	–	9·74 (-1·91 to 22·78)
East Asia	0	0·0	8	<0·1	–	9·23 (-1·16 to 20·70)
Oceania	0	0·0	0	0·0	–	–
Western Sub-Saharan Africa	0	0·0	0	0·0	–	–
Eastern Sub-Saharan Africa	0	0·0	0	0·0	–	–
Central Sub-Saharan Africa	0	0·0	0	0·0	–	–
Southern Sub-Saharan Africa	0	0·0	0	0·0	–	-12·28 (-36·11 to 20·44)

Abbreviations: PMP = per million population; PC = percent change; EAPC = estimated annual percent change; CI = confidence interval; PAK = pancreas after kidney; PTA = pancreas transplant alone. Notes:1.EAPC and 95% CIs were calculated by fitting a linear regression model to the natural logarithm of transplant rates (log[PMP]) across years (log[PMP] ~ Year). 2.A positive EAPC with a 95% CI not crossing zero indicates a statistically significant increasing trend; a negative EAPC with a 95% CI not crossing zero indicates a decreasing trend. 3."–" indicates that PC could not be estimated due to a zero baseline (i.e., 2008 = 0) or no transplants in both 2008 and 2023.

Appendix 1 Table S11: Trends in the number and rate of small bowel transplants by global region, 2008–2023, including percent change (PC) and estimated annual percent change (EAPC) with 95% confidence intervals

	2008		2023		2008-2023	
**Region classification**	**Small bowel transplants**	**PMP**	**Small bowel transplants**	**PMP**	**PC(%)**	**EAPC (%, 95% CI)**
**Global**	260	<0·1	187	<0·1	-28·07	-3·59 (-4·88 to -2·28)
**Human Development Index**						
Very High	244	0·2	148	0·1	-39·34	-5·61 (-7·11 to -4·08)
High	3	<0·1	23	<0·1	666·67	1·82 (-6·71 to 11·13)
Medium	13	<0·1	16	<0·1	23·08	-1·95 (-14·35 to 12·25)
Low	0	0·0	0	0·0	–	–
**World Health Organization Region**						
Africa	0	0·0	0	0·0	–	–
America	198	0·2	106	0·1	-46·46	-4·99 (-6·38 to -3·57)
Eastern Mediterranean	0	0·0	12	<0·1	–	23·45 (6·32 to 43·35)
Europe	49	0·1	37	<0·1	-24·49	-2·26 (-3·35 to -1·16)
South-East Asia	0	0·0	16	<0·1	–	26·07 (13·36 to 40·20)
Western Pacific	13	<0·1	16	<0·1	23·08	-2·83 (-12·28 to 7·63)
**Global Burden of Disease Region**						
High-income Asia Pacific	1	<0·1	3	<0·1	200·00	7·24 (-11·38 to 29·78)
High-income North America	189	0·6	102	0·3	-46·03	-4·59 (-6·12 to -3·04)
Western Europe	45	0·1	35	0·1	-22·22	-2·63 (-4·01 to -1·23)

Australasia	0	0·0	1	<0·1	–	37·40 (5·16 to 79·53)
Andean Latin America	0	0·0	1	<0·1	–	-8·75 (-32·53 to 23·42)
Tropical Latin America	0	0·0	1	<0·1	–	32·08 (7·75 to 61·90)
Central Latin America	1	<0·1	0	0·0	-100·00	-11·58 (-26·85 to 6·87)
Southern Latin America	8	0·1	2	<0·1	-75·00	-11·89 (-16·76 to -6·74)
Caribbean	0	0·0	0	0·0	–	–
Central Europe	0	0·0	0	0·0	–	37·64 (11·12 to 70·49)
Eastern Europe	0	0·0	1	<0·1	–	26·08 (5·41 to 50·80)
Central Asia	0	0·0	0	0·0	–	–
North Africa and Middle East	4	<0·1	13	<0·1	225·00	6·69 (-0·73 to 14·66)
South Asia	0	0·0	16	<0·1	–	26·09 (13·06 to 40·63)
Southeast Asia	12	<0·1	0	0·0	-100·00	-30·10 (-41·24 to -16·84)
East Asia	0	0·0	12	<0·1	–	20·38 (4·75 to 38·33)
Oceania	0	0·0	0	0·0	–	–
Western Sub-Saharan Africa	0	0·0	0	0·0	–	–
Eastern Sub-Saharan Africa	0	0·0	0	0·0	–	–
Central Sub-Saharan Africa	0	0·0	0	0·0	–	–
Southern Sub-Saharan Africa	0	0·0	0	0·0	–	–

Abbreviations: PMP = per million population; PC = percent change; EAPC = estimated annual percent change; CI = confidence interval.
Notes: 1.EAPC and 95% CIs were calculated by fitting a linear regression model to the natural logarithm of transplant rates (log[PMP]) across years (log[PMP] ~ Year). 2.A positive EAPC with a 95% CI not crossing zero indicates a statistically significant increasing trend; a negative EAPC with a 95% CI not crossing zero indicates a decreasing trend. 3.“–” indicates that PC could not be estimated due to a zero baseline (i.e., 2008 = 0) or no transplants in both 2008 and 2023.

Appendix 1 Table S12: Trends in the number and rate of total solid organ transplants by country, 2008–2023, including percent change (PC) and estimated annual percent change (EAPC) with 95% confidence intervals

	2008		2023		2008-2023	
**Country**	**Total transplants**	**PMP**	**Total transplants**	**PMP**	**PC(%)**	**EAPC (%, 95% CI)**
Afghanistan	0	0·0	459	10·9	-	-
Albania	1	0·3	26	9·3	2500·00	16·65 (9·87 to 23·86)
Algeria	115	3·3	93	2·1	-19·13	-1·66 (-5·75 to 2·61)

Argentina	1465	36·7	2196	48·0	49·90	1·03 (-0·46 to 2·53)
Armenia	8	2·7	26	9·3	225·00	8·48 (5·09 to 11·98)
Australia	1203	57·3	1714	64·9	42·48	0·85 (-0·21 to 1·92)
Austria	692	82·4	648	72·0	-6·36	-1·23 (-1·99 to -0·47)
Azerbaijan	83	9·8	41	4·0	-50·60	-5·18 (-7·57 to -2·73)
Bangladesh	28	0·2	319	1·8	1039·29	20·54 (17·20 to 23·97)
Barbados	1	3·3	1	3·3	0·00	0·00 (0·00 to 0·00)
Belarus	241	25·1	490	51·6	103·32	6·10 (4·25 to 7·98)
Belgium	892	85·0	1046	89·4	17·26	-0·68 (-1·71 to 0·36)
Bolivia (Plurinational State of)	79	8·1	62	5·0	-21·52	-6·27 (-11·38 to -0·87)
Bosnia and Herzegovina	15	3·9	14	4·4	-6·67	-3·47 (-7·96 to 1·24)
Brazil	5118	26·4	8725	40·3	70·48	1·63 (0·41 to 2·87)
Bulgaria	31	4·1	42	6·3	35·48	-0·41 (-5·72 to 5·20)
Canada	2141	64·5	3454	89·0	61·33	2·07 (1·32 to 2·81)
Chile	308	18·3	635	32·4	106·17	3·15 (1·21 to 5·13)
China	8255	6·2	23905	16·7	189·58	8·35 (6·35 to 10·39)
Colombia	1005	21·5	1423	27·3	41·59	-0·27 (-1·83 to 1·31)
Costa Rica	149	33·1	99	19·0	-33·56	-5·81 (-7·82 to -3·76)
Croatia	257	55·9	308	77·0	19·84	-0·59 (-2·64 to 1·50)
Cuba	176	15·6	13	1·2	-92·61	-12·23 (-19·37 to -4·46)
Cyprus	58	64·4	35	26·9	-39·66	-7·74 (-10·63 to -4·75)
Czech Republic	536	52·6	852	81·1	58·96	3·12 (2·13 to 4·12)
Denmark	278	50·6	437	74·1	57·19	1·31 (0·35 to 2·28)
Dominican Republic	103	10·4	81	7·2	-21·36	-1·73 (-4·96 to 1·61)
Ecuador	60	4·4	239	13·1	298·33	4·48 (0·21 to 8·94)
Egypt	1280	16·7	0	0·0	-100·00	-
El Salvador	29	4·1	47	7·3	62·07	-1·85 (-5·79 to 2·26)
Estonia	59	45·4	74	56·9	25·42	0·76 (-0·98 to 2·54)
Ethiopia	0	0·0	8	<0·1	-	-12·47 (-27·55 to 5·75)
Finland	230	43·4	475	86·4	106·52	3·99 (2·97 to 5·02)
France	4584	74·1	5642	87·1	23·08	0·86 (-0·08 to 1·81)
Georgia	7	1·6	59	16·0	742.86	13·90 (9·29 to 18·70)

Germany	4661	56·5	3646	43·8	-21·78	-2·54 (-3·21 to -1·85)
Ghana	1	<0·1	0	0·0	-100·00	-
Greece	316	28·2	307	29·8	-2·85	1·35 (-1·36 to 4·12)
Guatemala	85	6·2	111	6·1	30·59	-8·59 (-15·46 to -1·16)
Guyana	0	0·0	8	10·0	–	–
Honduras	4	0·6	4	0·4	0·00	-3·72 (-9·53 to 2·46)
Hungary	322	32·2	409	40·1	27·02	0·92 (-1·13 to 3·02)
Iceland	5	16·7	10	25·0	100·00	2·57 (0·11 to 5·09)
India	5855	4·9	18378	12·9	213·89	6·92 (4·85 to 9·02)
Indonesia	496	2·1	0	0·0	-100·00	-
Iran	2176	30·1	3510	40·8	61·31	0·34 (-1·46 to 2·18)
Ireland	220	50·0	265	52·0	20·45	-0·85 (-2·34 to 0·66)
Israel	281	40·1	653	71·0	132·38	5·28 (3·73 to 6·86)
Italy	3153	53·5	4543	77·1	44·08	2·09 (1·35 to 2·84)
Jamaica	2	0·7	2	0·7	0·00	1·13 (-1·18 to 3·50)
Japan	1713	13·4	2545	20·6	48·57	2·31 (1·56 to 3·06)
Jordan	210	34·4	224	19·8	6·67	-5·36 (-7·31 to -3·38)
Kazakhstan	168	10·8	236	12·0	40·48	-0·46 (-3·29 to 2·45)
Kenya	120	3·1	53	1·0	-55·83	3·00 (-5·29 to 12·02)
Kuwait	78	26·9	143	33·3	83·33	-0·76 (-2·87 to 1·39)
Kyrgyzstan	3	0·6	22	3·7	633·33	19·44 (13·02 to 26·22)
Latvia	55	23·9	50	27·8	-9·09	-2·20 (-4·37 to 0·01)
Lebanon	108	26·3	123	20·2	13·89	9·88 (-3·49 to 25·11)
Libyan	57	9·1	45	6·5	-21·05	-8·72 (-20·68 to 5·04)
Lithuania	68	20·0	147	54·4	116·18	4·71 (2·90 to 6·56)
Luxembourg	3	6·0	0	0·0	-100·00	-
Malaysia	52	1·9	308	9·0	492·31	7·00 (3·32 to 10·81)
Malta	22	55·0	5	10·0	-77·27	-7·39 (-12·58 to -1·90)
Mauritius	0	0·0	0	0·0	–	–
Mexico	2372	22·0	3426	26·7	44·44	-1·18 (-4·29 to 2·03)
Mongolia	4	1·5	141	41·5	3425·00	20·92 (12·85 to 29·58)
Montenegro	10	16·7	2	3·3	-80·00	-14·08 (-17·79 to -10·21)

Morocco	10	0·3	12	0·3	20·00	-0·04 (-7·84 to 8·41)
Myanmar	39	0·8	0	0·0	-100·00	-
Nepal	21	0·7	0	0·0	-100·00	-
Netherlands	996	60·4	1427	81·1	43·27	1·33 (0·59 to 2·07)
New Zealand	173	41·2	274	52·7	58·38	3·16 (1·79 to 4·56)
Nicaragua	0	0·0	3	0·5	-	-9·94 (-14·88 to -4·73)
Nigeria	14	<0·1	253	1·1	1707·14	17·87 (13·50 to 22·42)
Norway	436	92·8	402	73·1	-7·80	-2·04 (-2·76 to -1·32)
Oman	15	5·6	30	6·5	100·00	-7·22 (-16·51 to 3·11)
Pakistan	775	4·6	1850	7·7	138·71	1·64 (-4·61 to 8·29)
Panama	24	7·1	41	9·1	70·83	-5·38 (-10·97 to 0·56)
Paraguay	29	4·7	46	6·7	58·62	-0·08 (-5·61 to 5·77)
Peru	13	0·5	223	6·5	1615·38	7·34 (-2·61 to 18·31)
Philippines	1147	12·8	613	5·2	-46·56	-4·26 (-8·97 to 0·70)
Poland	1147	30·2	1932	47·1	68·44	1·34 (-0·01 to 2·71)
Portugal	858	80·2	920	90·2	7·23	0·20 (-0·97 to 1·40)
Qatar	4	4·4	88	32·6	2100·00	14·78 (9·00 to 20·88)
Republic of Korea	1802	37·2	4038	78·0	124·08	4·43 (3·11 to 5·76)
Republic of Moldova	0	0·0	4	1·2	-	2·16 (-11·77 to 18·28)
Republic of North Macedonia	12	6·0	25	11·9	108·33	2·84 (-2·90 to 8·93)
Romania	276	13·0	325	16·3	17·75	0·65 (-1·65 to 3·01)
Russian Federation	527	3·7	1962	13·5	272·30	7·66 (4·63 to 10·77)
Saudi Arabia	512	20·2	2051	55·6	300·59	5·74 (3·94 to 7·58)
Serbia	125	12·6	60	8·5	-52·00	-12·45 (-18·37 to -6·11)
Singapore	182	40·4	124	20·7	-31·87	-1·68 (-4·45 to 1·18)
Slovakia	204	37·8	188	32·4	-7·84	-0·31 (-2·00 to 1·41)
Slovenia	80	40·0	108	51·4	35·00	1·02 (-0·27 to 2·32)
South Africa	305	6·3	391	6·8	28·20	0·21 (-0·55 to 0·97)
Spain	3939	88·3	5863	123·4	48·84	2·25 (1·46 to 3·05)
Sri Lanka	300	15·5	275	12·6	-8·33	-0·99 (-1·57 to -0·40)
Sudan	68	1·7	205	4·6	201·47	5·59 (2·08 to 9·22)
Sweden	662	72·0	901	85·0	36·10	0·60 (-0·07 to 1·27)

Switzerland	456	60·8	686	78·0	50·44	1·00 (0·36 to 1·65)
Syrian Arab Republic	259	12·7	348	15·0	34·36	0·96 (-1·86 to 3·86)
Tajikistan	3	0·4	25	2·9	733·33	9·70 (1·18 to 18·93)
Thailand	393	6·1	1178	16·4	199·75	5·77 (3·75 to 7·82)
Trinidad and Tobago	12	9·2	4	2·7	-66·67	-6·33 (-9·88 to -2·66)
Tunisia	131	12·6	88	7·0	-32·82	-3·66 (-4·31 to -3·01)
Türkiye	2329	30·7	5277	61·5	126·58	3·11 (1·35 to 4·91)
Ukraine	132	2·9	591	16·1	347·73	8·02 (3·47 to 12·77)
United Arab Emirates	19	4·2	399	42·0	2000·00	16·04 (10·36 to 22·01)
United Kingdom	3051	50·0	4813	71·1	57·75	1·01 (-0·40 to 2·45)
United Republic of Tanzania	8	0·2	8	0·1	0·00	-2·51 (-2·76 to -2·26)
United States of America	27934	90·5	47492	139·7	70·02	3·30 (2·85 to 3·75)
Uruguay	145	42·7	174	51·2	20·00	1·39 (-0·36 to 3·16)
Venezuela	288	10·3	89	3·1	-69·10	-21·36 (-29·49 to -12·29)
Viet Nam	20	0·2	281	3·0	1305·00	15·40 (8·24 to 23·03)

Abbreviations: PMP = per million population; PC = percent change; EAPC = estimated annual percent change; CI = confidence interval.

Note: 1.PC was calculated as: ((2023 total transplants − 2008 total transplants) / 2008 total transplants) × 100%. 2.EAPC and 95% CIs were derived from a linear regression model fitted to the natural logarithm of PMP across years (log[PMP] ~ year). 3.A positive EAPC with a 95% CI that does not include 0 indicates a significant increasing trend; a negative EAPC with CI not crossing 0 indicates a significant decreasing trend. 4. "–" indicates values not calculable due to: 1) PC: zero transplants in 2008 (denominator = 0); 2) EAPC: insufficient valid data, including any of the following: a) fewer than 4 non-zero PMP values; b) non-zero years span less than 6 years; c) no valid transplant data for all three most recent years (2021–2023).

Appendix 1 Table S13: Estimated national population and global population share by country, 2008 and 2023

	2008		2023	
Country	Population(millions)	Population share(%)	Population(millions)	Population share(%)
Global	6741·4	100·00	7739·5	100·00
Afghanistan	28·2	0·42	42·2	0·55
Albania	3·2	0·05	2·8	0·04
Algeria	34·4	0·51	43·9	0·57
Andorra	0·1	<0·01	0·1	<0·01
Angola	17·5	0·26	22·1	0·29
Antigua and Barbuda	0·1	<0·01	0·1	<0·01

Argentina	39·9	0·59	45·8	0·59
Armenia	3·0	0·04	2·8	0·04
Australia	21·0	0·31	26·4	0·34
Austria	8·4	0·12	9·0	0·12
Azerbaijan	8·5	0·13	10·3	0·13
Bahamas	0·3	<0·01	0·4	0·01
Bahrain	0·8	0·01	1·3	0·02
Bangladesh	161·3	2·39	173·0	2·24
Barbados	0·3	<0·01	0·3	<0·01
Belarus	9·6	0·14	9·5	0·12
Belgium	10·5	0·16	11·7	0·15
Belize	0·3	<0·01	0·3	<0·01
Benin	9·3	0·14	10·6	0·14
Bhutan	0·7	0·01	0·8	0·01
Bolivia (Plurinational State of)	9·7	0·14	12·4	0·16
Bosnia and Herzegovina	3·9	0·06	3·2	0·04
Botswana	1·9	0·03	2·0	0·03
Brazil	194·2	2·88	216·4	2·80
Brunei Darussalam	0·4	0·01	0·4	0·01
Bulgaria	7·6	0·11	6·7	0·09
Burkina Faso	15·2	0·23	17·4	0·22
Burundi	8·9	0·13	10·5	0·14
Cambodia	14·7	0·22	15·4	0·20
Cameroon	18·9	0·28	23·3	0·30
Canada	33·2	0·49	38·8	0·50
Cape Verde	0·5	0·01	0·5	0·01
Central African Republic	4·4	0·07	4·7	0·06
Chad	11·1	0·16	13·2	0·17
Chile	16·8	0·25	19·6	0·25
China	1336·3	19·82	1433·9	18·53
Colombia	46·7	0·69	52·1	0·67
Comoros	0·9	0·01	0·8	0·01

Congo	3·8	0·06	4·6	0·06
Cook Islands	<0·1	<0·01	<0·1	<0·01
Costa Rica	4·5	0·07	5·2	0·07
Croatia	4·6	0·07	4·0	0·05
Cuba	11·3	0·17	11·2	0·14
Cyprus	0·9	0·01	1·3	0·02
Czech Republic	10·2	0·15	10·5	0·14
Côte d’Ivoire	19·6	0·29	20·8	0·27
Democratic People's Republic of Korea	23·9	0·35	25·0	0·32
Democratic Republic of The Congo	64·7	0·96	69·4	0·90
Denmark	5·5	0·08	5·9	0·08
Djibouti	0·8	0·01	0·9	0·01
Dominica	0·1	<0·01	0·1	<0·01
Dominican Republic	9·9	0·15	11·3	0·15
Ecuador	13·5	0·20	18·2	0·24
Egypt	76·8	1·14	91·5	1·18
El Salvador	7·0	0·10	6·4	0·08
Equatorial Guinea	0·5	0·01	0·8	0·01
Eritrea	5·0	0·07	6·5	0·08
Estonia	1·3	0·02	1·3	0·02
Eswatini	1·1	0·02	1·3	0·02
Ethiopia	85·2	1·26	126·5	1·63
Fiji	0·8	0·01	0·9	0·01
Finland	5·3	0·08	5·5	0·07
France	61·9	0·92	64·8	0·84
Gabon	1·4	0·02	1·7	0·02
Gambia	1·8	0·03	1·9	0·02
Georgia	4·4	0·07	3·7	0·05
Germany	82·5	1·22	83·3	1·08
Ghana	23·9	0·35	28·0	0·36
Greece	11·2	0·17	10·3	0·13
Grenada	0·1	<0·01	0·1	<0·01

Guatemala	13·7	0·20	18·1	0·23
Guinea	9·6	0·14	12·0	0·16
Guinea-Bissau	1·7	0·03	1·7	0·02
Guyana	0·7	0·01	0·8	0·01
Haiti	9·8	0·15	10·5	0·14
Honduras	7·2	0·11	10·2	0·13
Hungary	<0·1	0·15	10·2	0·13
Iceland	0·3	<0·01	0·4	0·01
India	1186·2	17·6	1428·6	18·46
Indonesia	234·3	3·48	252·8	3·27
Iran (Islamic Republic of)	72·2	1·07	86·0	1·11
Iraq	29·5	0·44	34·8	0·45
Ireland	4·4	0·07	5·1	0·07
Israel	7·0	0·10	9·2	0·12
Italy	58·9	0·87	58·9	0·76
Jamaica	2·7	0·04	2·8	0·04
Japan	127·9	1·90	123·3	1·59
Jordan	6·1	0·09	11·3	0·15
Kazakhstan	15·5	0·23	19·6	0·25
Kenya	38·6	0·57	55·1	0·71
Kiribati	0·1	<0·01	0·1	<0·01
Kuwait	2·9	0·04	4·3	0·06
Kyrgyzstan	5·4	0·08	5·9	0·08
Lao People's Democratic Republic	6·1	0·09	7·6	0·10
Latvia	2·3	0·03	1·8	0·02
Lebanon	4·1	0·06	6·1	0·08
Lesotho	2·0	0·03	2·1	0·03
Liberia	3·9	0·06	4·4	0·06
Libyan Arab Jamahiriya	6·3	0·09	6·9	0·09
Lithuania	3·4	0·05	2·7	0·03
Luxembourg	0·5	0·01	0·7	0·01
Madagascar	20·2	0·30	23·6	0·30

Malawi	14·3	0·21	16·8	0·22
Malaysia	27·0	0·40	34·3	0·44
Maldives	0·3	<0·01	0·4	0·01
Mali	12·7	0·19	17·6	0·23
Malta	0·4	0·01	0·5	0·01
Marshall Islands	0·6	0·01	0·6	0·01
Mauritania	3·2	0·05	4·0	0·05
Mauritius	1·3	0·02	1·3	0·02
Mexico	107·8	1·60	128·5	1·66
Micronesia (Federated States of)	0·6	0·01	0·1	<0·01
Monaco	<0·1	<0·01	<0·1	<0·01
Mongolia	2·7	0·04	3·4	0·04
Montenegro	0·6	0·01	0·6	0·01
Morocco	31·6	0·47	37·3	0·48
Mozambique	21·8	0·32	26·5	0·34
Myanmar	49·2	0·73	53·7	0·69
Namibia	2·1	0·03	2·3	0·03
Nauru	<0·1	<0·01	<0·1	<0·01
Nepal	28·8	0·43	28·5	0·37
Netherlands	16·5	0·24	17·6	0·23
New Zealand	4·2	0·06	5·2	0·07
Nicaragua	5·7	0·08	6·7	0·09
Niger	14·7	0·22	18·5	0·24
Nigeria	151·5	2·25	223·8	2·89
Niue	<0·1	<0·01	<0·1	<0·01
Norway	4·7	0·07	5·5	0·07
Oman	2·7	0·04	4·6	0·06
Pakistan	167·0	2·48	240·5	3·11
Palau	<0·1	<0·01	<0·1	<0·01
Panama	3·4	0·05	4·5	0·06
Papua New Guinea	6·5	0·10	7·5	0·10
Paraguay	6·2	0·09	6·9	0·09

Peru	28·2	0·42	34·4	0·44
Philippines	89·7	1·33	117·3	1·52
Poland	38·0	0·56	41·0	0·53
Portugal	10·7	0·16	10·2	0·13
Qatar	0·9	0·01	2·7	0·03
Republic of Korea	48·4	0·72	51·8	0·67
Republic of Moldova	3·8	0·06	3·4	0·04
Republic of North Macedonia	2·0	0·03	2·1	0·03
Romania	21·3	0·32	19·9	0·26
Russian Federation	141·8	2·10	145·9	1·89
Rwanda	<0·1	0·15	12·1	0·16
Saint Kitts and Nevis	<0·1	<0·01	0·1	<0·01
Saint Lucia	0·2	<0·01	0·2	<0·01
Saint Vincent and the Grenadines	0·1	<0·01	0.1	<0·01
Samoa	0·2	<0·01	0.2	<0·01
San Marino	<0·1	<0·01	<0·1	<0·01
Sao Tome and Principe	0·2	<0·01	0·2	<0·01
Saudi Arabia	25·3	0·38	36·9	0·48
Senegal	12·7	0·19	16·3	0·21
Serbia	9·9	0·15	7·1	0·09
Seychelles	0·1	<0·01	0·1	<0·01
Sierra Leone	6·0	0·09	6·2	0·08
Singapore	4·5	0·07	6·0	0·08
Slovakia	5·4	0·08	5·8	0·07
Slovenia	2·0	0·03	2·1	0·03
Solomon Islands	0·5	0·01	0·6	0·01
Somalia	9·0	0·13	10·8	0·14
South Africa	48·8	0·72	57·4	0·74
South Sudan	10·7	0·16	11·7	0·15
Spain	44·6	0·66	47·5	0·61
Sri Lanka	19·4	0·29	21·9	0·28
Sudan	39·4	0·58	44·9	0·58

Suriname	0·5	0·01	0·5	0·01
Sweden	9·2	0·14	10·6	0·14
Switzerland	7·5	0·11	8·8	0·11
Syrian Arab Republic	20·4	0·30	23·2	0·30
Tajikistan	6·8	0·10	8·5	0·11
Thailand	64·3	0·95	71·8	0·93
Timor-Leste	1·2	0·02	1·2	0·02
Togo	6·8	0·10	7·0	0·09
Tonga	0·1	<0·01	0·1	<0·01
Trinidad and Tobago	1·3	0·02	1·5	0·02
Tunisia	10·4	0·15	12·5	0·16
Turkmenistan	5·0	0·07	5·3	0·07
Tuvalu	<0·1	<0·01	<0·1	<0·01
Türkiye	75·8	1·12	85·8	1·11
Uganda	31·9	0·47	38·8	0·50
Ukraine	45·9	0·68	36·7	0·47
United Arab Emirates	4·5	0·07	9·5	0·12
United Kingdom	61·0	0·90	67·7	0·87
United Republic of Tanzania	41·5	0·62	59·7	0·77
United States of America	308·8	4·58	340·1	4·39
Uruguay	3·4	0·05	3·4	0·04
Uzbekistan	27·8	0·41	29·9	0·39
Vanuatu	0·2	<0·01	0·3	<0·01
Venezuela (Bolivarian Republic of)	28·1	0·42	28·8	0·37
Viet Nam	88·5	1·31	93·4	1·21
Yemen	23·1	0·34	25·0	0·32
Zambia	12·2	0·18	15·0	0·19
Zimbabwe	13·5	0·20	14·6	0·19

Population values are in millions (M). Share (%) represents the proportion of the global population for each year. Population values <0·1 million are reported as "<0·1". Values <0·01% denote non-zero shares below two-decimal precision.

Appendix 1 Table S14: Global Distribution of Solid Organ Transplantation Capacity and Corresponding Population Proportion, 2008–2010

Country	Population(millions)	Global Pop· Share (%)	Kidney	Liver	Heart	Lung	Pancreas	Small Bowel
Afghanistan	28·2	0·42	No	No	No	No	No	No
Albania	3·2	0·05	Yes	No	No	No	No	No
Algeria	34·4	0·51	Yes	Yes	No	No	No	No
Andorra	0·1	<0·01	No	No	No	No	No	No
Angola	17·5	0·26	No	No	No	No	No	No
Antigua and Barbuda	0·1	<0·01	No	No	No	No	No	No
Argentina	39·9	0·59	Yes	Yes	Yes	Yes	Yes	Yes
Armenia	3.0	0·04	Yes	No	No	No	No	No
Australia	21.0	0·31	Yes	Yes	Yes	Yes	Yes	Yes
Austria	8·4	0·12	Yes	Yes	Yes	Yes	Yes	No
Azerbaijan	8·5	0·13	Yes	Yes	No	No	No	No
Bahamas	0·3	<0·01	No	No	No	No	No	No
Bahrain	0·8	0·01	No	No	No	No	No	No
Bangladesh	161·3	2·39	Yes	No	No	No	No	No
Barbados	0·3	<0·01	Yes	No	No	No	No	No
Belarus	9·6	0·14	Yes	Yes	Yes	No	Yes	No
Belgium	10·5	0·16	Yes	Yes	Yes	Yes	Yes	No
Belize	0·3	<0·01	No	No	No	No	No	No
Benin	9·3	0·14	No	No	No	No	No	No
Bhutan	0·7	0·01	No	No	No	No	No	No
Bolivia (Plurinational State of)	9·7	0·14	Yes	No	No	No	No	No
Bosnia and Herzegovina	3·9	0·06	Yes	No	No	No	No	No
Botswana	1·9	0·03	No	No	No	No	No	No
Brazil	194·2	2·88	Yes	Yes	Yes	Yes	Yes	No
Brunei Darussalam	0·4	<0·01	No	No	No	No	No	No
Bulgaria	7·6	0·11	Yes	Yes	Yes	No	No	No
Burkina Faso	15·2	0·23	No	No	No	No	No	No
Burundi	8·9	0·13	No	No	No	No	No	No
Cambodia	14·7	0·22	No	No	No	No	No	No
Cameroon	18·9	0·28	No	No	No	No	No	No
Canada	33·2	0·49	Yes	Yes	Yes	Yes	Yes	Yes
Cape Verde	0·5	<0·01	No	No	No	No	No	No

Central African Republic	4·4	0·07	No	No	No	No	No	No
Chad	11·1	0·16	No	No	No	No	No	No
Chile	16·8	0·25	Yes	Yes	Yes	Yes	No	No
China	1336·3	19·82	Yes	Yes	Yes	Yes	No	No
Colombia	46·7	0·69	Yes	Yes	Yes	Yes	Yes	Yes
Comoros	0·9	0·01	No	No	No	No	No	No
Congo	3·8	0·06	No	No	No	No	No	No
Cook Islands	<0·1	<0·01	No	No	No	No	No	No
Costa Rica	4·5	0·07	Yes	Yes	Yes	No	No	No
Croatia	4·6	0·07	Yes	Yes	Yes	No	Yes	No
Cuba	11·3	0·17	Yes	Yes	Yes	No	Yes	No
Cyprus	0·9	0·01	Yes	No	No	No	No	No
Czech Republic	10·2	0·15	Yes	Yes	Yes	Yes	Yes	No
Côte d'Ivoire	19·6	0·29	No	No	No	No	No	No
Democratic People's Republic of Korea	23·9	0·35	No	No	No	No	No	No
Democratic Republic of The Congo	64·7	0·96	No	No	No	No	No	No
Denmark	5·5	0·08	Yes	Yes	Yes	Yes	No	No
Djibouti	0·8	0·01	No	No	No	No	No	No
Dominica	0·1	<0·01	No	No	No	No	No	No
Dominican Republic	9·9	0·15	Yes	Yes	No	No	No	No
Ecuador	13·5	0·20	Yes	Yes	Yes	No	No	No
Egypt	76·8	1·14	Yes	Yes	No	No	No	No
El Salvador	7.0	0·10	Yes	No	No	No	No	No
Equatorial Guinea	0·5	<0·01	No	No	No	No	No	No
Eritrea	5.0	0·07	No	No	No	No	No	No
Estonia	1·3	0·02	Yes	Yes	No	Yes	No	No
Eswatini	1·1	0·02	No	No	No	No	No	No
Ethiopia	85·2	1·26	No	No	No	No	No	No
Fiji	0·8	0·01	No	No	No	No	No	No
Finland	5·3	0·08	Yes	Yes	Yes	Yes	No	Yes
France	61·9	0·92	Yes	Yes	Yes	Yes	Yes	Yes
Gabon	1·4	0·02	No	No	No	No	No	No
Gambia	1·8	0·03	No	No	No	No	No	No
Georgia	4·4	0·07	Yes	No	No	No	No	No

Germany	82·5	1·22	Yes	Yes	Yes	Yes	Yes	Yes
Ghana	23·9	0·35	Yes	No	No	No	No	No
Greece	11·2	0·17	Yes	Yes	Yes	Yes	Yes	No
Grenada	0·1	<0·01	No	No	No	No	No	No
Guatemala	13·7	0·20	Yes	No	No	No	No	No
Guinea	9·6	0·14	No	No	No	No	No	No
Guinea-Bissau	1·7	0·03	No	No	No	No	No	No
Guyana	0·7	0·01	No	No	No	No	No	No
Haiti	9·8	0·15	No	No	No	No	No	No
Honduras	7·2	0·11	Yes	No	No	No	No	No
Hungary	10·0	0·15	Yes	Yes	Yes	No	Yes	No
Iceland	0·3	<0·01	Yes	No	No	No	No	No
India	1186·2	17·6	Yes	Yes	Yes	No	No	Yes
Indonesia	234·3	3·48	Yes	Yes	No	No	No	No
Iran (Islamic Republic of)	72·2	1·07	Yes	Yes	Yes	Yes	Yes	Yes
Iraq	29·5	0·44	No	No	No	No	No	No
Ireland	4·4	0·07	Yes	Yes	Yes	Yes	Yes	No
Israel	7.0	0·10	Yes	Yes	Yes	Yes	Yes	Yes
Italy	58·9	0·87	Yes	Yes	Yes	Yes	Yes	Yes
Jamaica	2·7	0·04	No	Yes	No	No	No	No
Japan	127·9	1·90	Yes	Yes	Yes	Yes	Yes	Yes
Jordan	6·1	0·09	Yes	Yes	No	No	No	No
Kazakhstan	15·5	0·23	Yes	Yes	Yes	No	Yes	No
Kenya	38·6	0·57	Yes	No	No	No	No	No
Kiribati	0·1	<0·01	No	No	No	No	No	No
Kuwait	2·9	0·04	Yes	Yes	No	No	Yes	No
Kyrgyzstan	5·4	0·08	Yes	No	No	No	No	No
Lao People's Democratic Republic	6·1	0·09	No	No	No	No	No	No
Latvia	2·3	0·03	Yes	No	Yes	No	Yes	No
Lebanon	4·1	0·06	Yes	Yes	No	No	No	No
Lesotho	2·0	0·03	No	No	No	No	No	No
Liberia	3·9	0·06	No	No	No	No	No	No
Libyan Arab Jamahiriya	6·3	0·09	Yes	Yes	No	No	No	No
Lithuania	3·4	0·05	Yes	Yes	Yes	Yes	Yes	No

Luxembourg	0·5	<0·01	Yes	Yes	Yes	Yes	No	Yes
Madagascar	20·2	0·30	No	No	No	No	No	No
Malawi	14·3	0·21	No	No	No	No	No	No
Malaysia	27.0	0·40	Yes	Yes	Yes	Yes	No	No
Maldives	0·3	<0·01	No	No	No	No	No	No
Mali	12·7	0·19	No	No	No	No	No	No
Malta	0·4	<0·01	Yes	Yes	Yes	No	No	No
Marshall Islands	0·6	<0·01	No	No	No	No	No	No
Mauritania	3·2	0·05	No	No	No	No	No	No
Mauritius	1·3	0·02	Yes	No	No	No	No	No
Mexico	107·8	1·60	Yes	Yes	Yes	Yes	Yes	No
Micronesia (Federated States of)	0·6	<0·01	No	No	No	No	No	No
Monaco	<0·1	<0·01	No	No	No	No	No	No
Mongolia	2·7	0·04	Yes	No	No	No	No	No
Montenegro	0·6	<0·01	Yes	No	No	No	No	No
Morocco	31·6	0·47	Yes	No	No	No	No	No
Mozambique	21·8	0·32	No	No	No	No	No	No
Myanmar	49·2	0·73	Yes	Yes	No	No	No	No
Namibia	2·1	0·03	No	No	No	No	No	No
Nauru	<0·1	<0·01	No	No	No	No	No	No
Nepal	28·8	0·43	Yes	No	No	No	No	No
Netherlands	16·5	0·24	Yes	Yes	Yes	Yes	Yes	Yes
New Zealand	4·2	0·06	Yes	Yes	Yes	Yes	Yes	No
Nicaragua	5·7	0·08	Yes	No	No	No	No	No
Niger	14·7	0·22	No	No	No	No	No	No
Nigeria	151·5	2·25	Yes	No	No	No	No	No
Niue	0·0	<0·01	No	No	No	No	No	No
Norway	4·7	0·07	Yes	Yes	Yes	Yes	Yes	No
Oman	2·7	0·04	Yes	No	No	No	No	No
Pakistan	167.0	2·48	Yes	No	No	No	No	No
Palau	<0·1	<0·01	No	No	No	No	No	No
Panama	3·4	0·05	Yes	No	No	No	No	No
Papua New Guinea	6·5	0·10	No	No	No	No	No	No
Paraguay	6·2	0·09	Yes	No	Yes	No	No	Yes

Peru	28·2	0·42	Yes	Yes	Yes	Yes	No	No
Philippines	89·7	1·33	Yes	No	No	No	No	No
Poland	38.0	0·56	Yes	Yes	Yes	Yes	Yes	No
Portugal	10·7	0·16	Yes	Yes	Yes	Yes	Yes	No
Qatar	0·9	0·01	Yes	No	No	No	No	No
Republic of Korea	48·4	0·72	Yes	Yes	Yes	Yes	Yes	Yes
Republic of Moldova	3·8	0·06	No	No	No	No	No	No
Republic of North Macedonia	2·0	0·03	Yes	No	No	No	No	No
Romania	21·3	0·32	Yes	Yes	Yes	No	No	No
Russian Federation	141·8	2·10	Yes	Yes	Yes	Yes	No	No
Rwanda	10·0	0·15	No	No	No	No	No	No
Saint Kitts and Nevis	0·0	<0·01	No	No	No	No	No	No
Saint Lucia	0·2	<0·01	No	No	No	No	No	No
Saint Vincent and the Grenadines	0·1	<0·01	No	No	No	No	No	No
Samoa	0·2	<0·01	No	No	No	No	No	No
San Marino	<0·1	<0·01	No	No	No	No	No	No
Sao Tome and Principe	0·2	<0·01	No	No	No	No	No	No
Saudi Arabia	25·3	0·38	Yes	Yes	Yes	Yes	Yes	No
Senegal	12·7	0·19	No	No	No	No	No	No
Serbia	9·9	0·15	Yes	Yes	Yes	No	No	No
Seychelles	0·1	<0·01	No	No	No	No	No	No
Sierra Leone	6·0	0·09	No	No	No	No	No	No
Singapore	4·5	0·07	Yes	Yes	Yes	No	No	No
Slovakia	5·4	0·08	Yes	Yes	Yes	No	No	No
Slovenia	2·0	0·03	Yes	Yes	Yes	No	No	No
Solomon Islands	0·5	<0·01	No	No	No	No	No	No
Somalia	9·0	0·13	No	No	No	No	No	No
South Africa	48·8	0·72	Yes	Yes	Yes	Yes	Yes	No
South Sudan	10·7	0·16	No	No	No	No	No	No
Spain	44·6	0·66	Yes	Yes	Yes	Yes	Yes	Yes
Sri Lanka	19·4	0·29	Yes	No	No	No	No	No
Sudan	39·4	0·58	Yes	No	No	No	No	No
Suriname	0·5	<0·01	No	No	No	No	No	No
Sweden	9·2	0·14	Yes	Yes	Yes	Yes	No	No

Switzerland	7·5	0·11	Yes	Yes	Yes	Yes	Yes	Yes
Syrian Arab Republic	20·4	0·30	Yes	No	No	No	No	No
Tajikistan	6·8	0·10	Yes	No	No	No	No	No
Thailand	64·3	0·95	Yes	Yes	Yes	No	No	No
Timor-Leste	1·2	0·02	No	No	No	No	No	No
Togo	6·8	0·10	No	No	No	No	No	No
Tonga	0·1	<0·01	No	No	No	No	No	No
Trinidad and Tobago	1·3	0·02	Yes	No	No	No	No	No
Tunisia	10·4	0·15	Yes	Yes	No	No	No	No
Turkmenistan	5·0	0·07	No	No	No	No	No	No
Tuvalu	<0·1	<0·01	No	No	No	No	No	No
Türkiye	75·8	1·12	Yes	Yes	Yes	Yes	Yes	Yes
Uganda	31·9	0·47	No	No	No	No	No	No
Ukraine	45·9	0·68	Yes	Yes	No	No	No	No
United Arab Emirates	4·5	0·07	Yes	No	No	No	No	No
United Kingdom	61·0	0·90	Yes	Yes	Yes	Yes	Yes	Yes
United Republic of Tanzania	41·5	0·62	Yes	No	No	No	No	No
United States of America	308·8	4·58	Yes	Yes	Yes	Yes	Yes	Yes
Uruguay	3·4	0·05	Yes	Yes	Yes	Yes	Yes	No
Uzbekistan	27·8	0·41	No	No	No	No	No	No
Vanuatu	0·2	<0·01	No	No	No	No	No	No
Venezuela (Bolivarian Republic of)	28·1	0·42	Yes	Yes	No	No	No	No
Viet Nam	88·5	1·31	Yes	Yes	No	No	No	Yes
Yemen	23·1	0·34	No	No	No	No	No	No
Zambia	12·2	0·18	No	No	No	No	No	No
Zimbabwe	13·5	0·20	No	No	No	No	No	No

Note: A country is classified as "Yes" for a specific organ if it reported at least one transplant procedure between 2008 and 2010. "Population (millions)" is rounded to one decimal places; values below 0·1 million are shown as "<0·1". "Global Pop· Share (%)" represents the proportion of each country's population relative to the total population of all countries included; values below 0·01% are shown as "<0·01".

Appendix 1 Table S15: Global Distribution of Solid Organ Transplantation Capacity and Corresponding Population Proportion, 2021–2023

Country	Population(millions)	Global Pop· Share (%)	Kidney	Liver	Heart	Lung	Pancreas	Small Bowel
Afghanistan	39·6	0·52	Yes	No	No	No	No	No

Albania	2·9	0·04	Yes	No	No	No	No	No
Algeria	43·9	0·57	Yes	Yes	No	No	No	No
Andorra	0·1	<0·01	No	No	No	No	No	No
Angola	22·1	0·29	No	No	No	No	No	No
Antigua and Barbuda	0·1	<0·01	No	No	No	No	No	No
Argentina	45·6	0·60	Yes	Yes	Yes	Yes	Yes	Yes
Armenia	3·0	0·04	Yes	Yes	No	No	No	No
Australia	25·8	0·34	Yes	Yes	Yes	Yes	Yes	Yes
Austria	9·0	0·12	Yes	Yes	Yes	Yes	Yes	Yes
Azerbaijan	10·2	0·13	Yes	Yes	No	No	No	No
Bahamas	0·4	<0·01	No	No	No	No	No	No
Bahrain	1·3	0·02	No	No	No	No	No	No
Bangladesh	166·3	2·17	Yes	No	No	No	No	No
Barbados	0·3	<0·01	Yes	No	No	No	No	No
Belarus	9·4	0·12	Yes	Yes	Yes	Yes	Yes	No
Belgium	11·6	0·15	Yes	Yes	Yes	Yes	Yes	Yes
Belize	0·3	<0·01	No	No	No	No	No	No
Benin	10·6	0·14	No	No	No	No	No	No
Bhutan	0·8	0·01	No	No	No	No	No	No
Bolivia (Plurinational State of)	11·8	0·15	Yes	Yes	No	No	No	No
Bosnia and Herzegovina	3·3	0·04	Yes	Yes	No	No	No	No
Botswana	2·0	0·03	No	No	No	No	No	No
Brazil	214·0	2·80	Yes	Yes	Yes	Yes	Yes	Yes
Brunei Darussalam	0·4	<0·01	No	No	No	No	No	No
Bulgaria	6·9	0·09	Yes	Yes	Yes	No	No	No
Burkina Faso	17·4	0·23	No	No	No	No	No	No
Burundi	10·5	0·14	No	No	No	No	No	No
Cambodia	15·4	0·20	No	No	No	No	No	No
Cameroon	23·3	0·30	No	No	No	No	No	No
Canada	38·1	0·50	Yes	Yes	Yes	Yes	Yes	Yes
Cape Verde	0·5	<0·01	No	No	No	No	No	No
Central African Republic	4·7	0·06	No	No	No	No	No	No

Chad	13·2	0·17	No	No	No	No	No	No
Chile	19·2	0·25	Yes	Yes	Yes	Yes	Yes	No
China	1452·5	19·0	Yes	Yes	Yes	Yes	Yes	Yes
Colombia	51·3	0·67	Yes	Yes	Yes	Yes	Yes	Yes
Comoros	0·8	0·01	No	No	No	No	No	No
Congo	4·6	0·06	No	No	No	No	No	No
Cook Islands	<0·1	<0·01	No	No	No	No	No	No
Costa Rica	5·1	0·07	Yes	Yes	Yes	Yes	No	No
Croatia	4·1	0·05	Yes	Yes	Yes	Yes	Yes	Yes
Cuba	11·3	0·15	Yes	Yes	No	No	No	No
Cyprus	1·2	0·02	Yes	No	No	No	No	No
Czech Republic	10·7	0·14	Yes	Yes	Yes	Yes	Yes	Yes
Côte d’Ivoire	20·8	0·27	No	No	No	No	No	No
Democratic People's Republic of Korea	25·0	0·33	No	No	No	No	No	No
Democratic Republic of The Congo	69·4	0·91	No	No	No	No	No	No
Denmark	5·8	0·08	Yes	Yes	Yes	Yes	Yes	No
Djibouti	0·9	0·01	No	No	No	No	No	No
Dominica	0·1	<0·01	No	No	No	No	No	No
Dominican Republic	11·0	0·14	Yes	Yes	No	No	No	No
Ecuador	17·9	0·23	Yes	Yes	Yes	No	No	No
Egypt	91·5	1·20	No	No	No	No	No	No
El Salvador	6·5	0·08	Yes	No	No	No	No	No
Equatorial Guinea	0·8	0·01	No	No	No	No	No	No
Eritrea	6·5	0·08	No	No	No	No	No	No
Estonia	1·3	0·02	Yes	Yes	No	Yes	Yes	No
Eswatini	1·3	0·02	No	No	No	No	No	No
Ethiopia	117·9	1·54	Yes	No	No	No	No	No
Fiji	0·9	0·01	No	No	No	No	No	No
Finland	5·5	0·07	Yes	Yes	Yes	Yes	Yes	Yes
France	65·4	0·85	Yes	Yes	Yes	Yes	Yes	Yes
Gabon	1·7	0·02	No	No	No	No	No	No
Gambia	1·9	0·02	No	No	No	No	No	No

Georgia	4·0	0·05	Yes	Yes	No	No	No	No
Germany	83·9	1·10	Yes	Yes	Yes	Yes	Yes	Yes
Ghana	28·0	0·37	No	No	No	No	No	No
Greece	10·4	0·14	Yes	Yes	Yes	Yes	No	No
Grenada	0·1	<0·01	No	No	No	No	No	No
Guatemala	18·2	0·24	Yes	No	No	No	No	No
Guinea	12·0	0·16	No	No	No	No	No	No
Guinea-Bissau	1·7	0·02	No	No	No	No	No	No
Guyana	0·8	0·01	Yes	No	No	No	No	No
Haiti	10·5	0·14	No	No	No	No	No	No
Honduras	10·1	0·13	Yes	No	No	No	No	No
Hungary	9·6	0·13	Yes	Yes	Yes	Yes	Yes	No
Iceland	0·3	<0·01	Yes	No	No	No	No	No
India	1393·4	18·21	Yes	Yes	Yes	Yes	Yes	Yes
Indonesia	252·8	3·30	No	No	No	No	No	No
Iran (Islamic Republic of)	85·0	1·11	Yes	Yes	Yes	Yes	Yes	Yes
Iraq	34·8	0·45	No	No	No	No	No	No
Ireland	5·0	0·07	Yes	Yes	Yes	Yes	Yes	No
Israel	8·8	0·12	Yes	Yes	Yes	Yes	Yes	No
Italy	60·4	0·79	Yes	Yes	Yes	Yes	Yes	Yes
Jamaica	3·0	0·04	Yes	Yes	No	No	No	No
Japan	126·1	1·65	Yes	Yes	Yes	Yes	Yes	Yes
Jordan	10·3	0·13	Yes	Yes	No	No	No	No
Kazakhstan	19·0	0·25	Yes	Yes	Yes	Yes	No	No
Kenya	55·0	0·72	Yes	No	No	No	No	No
Kiribati	0·1	<0·01	No	No	No	No	No	No
Kuwait	4·3	0·06	Yes	No	Yes	No	Yes	No
Kyrgyzstan	5·9	0·08	Yes	Yes	No	No	No	No
Lao People's Democratic Republic	7·4	0·10	No	No	No	No	No	No
Latvia	1·9	0·02	Yes	Yes	Yes	No	No	No
Lebanon	6·1	0·08	Yes	Yes	Yes	Yes	No	No
Lesotho	2·1	0·03	No	No	No	No	No	No

Liberia	4·4	0·06	No	No	No	No	No	No
Libyan Arab Jamahiriya	7·0	0·09	Yes	No	No	No	No	No
Lithuania	2·7	0·04	Yes	Yes	Yes	Yes	Yes	No
Luxembourg	0·6	<0·01	No	No	No	No	No	No
Madagascar	23·6	0·31	No	No	No	No	No	No
Malawi	16·8	0·22	No	No	No	No	No	No
Malaysia	32·8	0·43	Yes	Yes	No	No	No	No
Maldives	0·4	<0·01	No	No	No	No	No	No
Mali	17·6	0·23	No	No	No	No	No	No
Malta	0·4	<0·01	Yes	No	No	No	No	No
Marshall Islands	0·6	<0·01	No	No	No	No	No	No
Mauritania	4·0	0·05	No	No	No	No	No	No
Mauritius	1·3	0·02	No	No	No	No	No	No
Mexico	130·3	1·70	Yes	Yes	Yes	Yes	Yes	No
Micronesia (Federated States of)	0·1	<0·01	No	No	No	No	No	No
Monaco	<0·1	<0·01	No	No	No	No	No	No
Mongolia	3·3	0·04	Yes	Yes	No	No	No	No
Montenegro	0·6	<0·01	Yes	No	No	No	No	No
Morocco	37·3	0·49	Yes	No	No	No	No	No
Mozambique	26·5	0·35	No	No	No	No	No	No
Myanmar	53·7	0·70	No	No	No	No	No	No
Namibia	2·3	0·03	No	No	No	No	No	No
Nauru	<0·1	<0·01	No	No	No	No	No	No
Nepal	28·5	0·37	No	No	No	No	No	No
Netherlands	17·2	0·22	Yes	Yes	Yes	Yes	Yes	Yes
New Zealand	4·9	0·06	Yes	Yes	Yes	Yes	Yes	No
Nicaragua	6·7	0·09	Yes	No	No	No	No	No
Niger	18·5	0·24	No	No	No	No	No	No
Nigeria	211·4	2·76	Yes	No	No	No	No	No
Niue	0·0	<0·01	No	No	No	No	No	No
Norway	5·5	0·07	Yes	Yes	Yes	Yes	Yes	No
Oman	5·2	0·07	Yes	Yes	No	No	No	No

Pakistan	225·2	2·94	Yes	Yes	No	No	No	No
Palau	<0·1	<0·01	No	No	No	No	No	No
Panama	4·4	0·06	Yes	Yes	Yes	No	No	No
Papua New Guinea	7·5	0·10	No	No	No	No	No	No
Paraguay	7·2	0·09	Yes	Yes	Yes	No	No	No
Peru	33·4	0·44	Yes	Yes	Yes	Yes	Yes	No
Philippines	111·0	1·45	Yes	Yes	No	No	No	No
Poland	37·8	0·49	Yes	Yes	Yes	Yes	Yes	No
Portugal	10·2	0·13	Yes	Yes	Yes	Yes	Yes	No
Qatar	2·9	0·04	Yes	Yes	No	Yes	No	No
Republic of Korea	51·3	0·67	Yes	Yes	Yes	Yes	Yes	Yes
Republic of Moldova	4·0	0·05	Yes	Yes	No	No	No	No
Republic of North Macedonia	2·1	0·03	Yes	Yes	Yes	No	No	No
Romania	19·1	0·25	Yes	Yes	Yes	Yes	No	No
Russian Federation	145·9	1·91	Yes	Yes	Yes	Yes	Yes	Yes
Rwanda	12·1	0·16	No	No	No	No	No	No
Saint Kitts and Nevis	0·1	<0·01	No	No	No	No	No	No
Saint Lucia	0·2	<0·01	No	No	No	No	No	No
Saint Vincent and the Grenadines	0·1	<0·01	No	No	No	No	No	No
Samoa	0·2	<0·01	No	No	No	No	No	No
San Marino	<0·1	<0·01	No	No	No	No	No	No
Sao Tome and Principe	0·2	<0·01	No	No	No	No	No	No
Saudi Arabia	35·3	0·46	Yes	Yes	Yes	Yes	Yes	Yes
Senegal	16·3	0·21	No	No	No	No	No	No
Serbia	8·7	0·11	Yes	Yes	Yes	No	No	No
Seychelles	0·1	<0·01	No	No	No	No	No	No
Sierra Leone	6·2	0·08	No	No	No	No	No	No
Singapore	5·9	0·08	Yes	Yes	Yes	Yes	Yes	No
Slovakia	5·5	0·07	Yes	Yes	Yes	No	No	No
Slovenia	2·1	0·03	Yes	Yes	Yes	Yes	Yes	No
Solomon Islands	0·6	<0·01	No	No	No	No	No	No
Somalia	10·8	0·14	No	No	No	No	No	No

South Africa	57·4	0·75	Yes	Yes	Yes	Yes	Yes	No
South Sudan	11·7	0·15	No	No	No	No	No	No
Spain	46·7	0·61	Yes	Yes	Yes	Yes	Yes	Yes
Sri Lanka	21·5	0·28	Yes	Yes	Yes	Yes	No	No
Sudan	44·9	0·59	Yes	No	No	No	No	No
Suriname	0·5	<0·01	No	No	No	No	No	No
Sweden	10·2	0·13	Yes	Yes	Yes	Yes	Yes	Yes
Switzerland	8·7	0·11	Yes	Yes	Yes	Yes	Yes	Yes
Syrian Arab Republic	18·3	0·24	Yes	No	No	No	No	No
Tajikistan	8·5	0·11	Yes	No	No	No	No	No
Thailand	70·0	0·91	Yes	Yes	Yes	Yes	Yes	Yes
Timor-Leste	1·2	0·02	No	No	No	No	No	No
Togo	7·0	0·09	No	No	No	No	No	No
Tonga	0·1	<0·01	No	No	No	No	No	No
Trinidad and Tobago	1·4	0·02	Yes	No	No	No	No	No
Tunisia	11·9	0·16	Yes	Yes	Yes	No	No	No
Turkmenistan	5·3	0·07	No	No	No	No	No	No
Tuvalu	<0·1	<0·01	No	No	No	No	No	No
Türkiye	85·0	1·11	Yes	Yes	Yes	Yes	Yes	Yes
Uganda	38·8	0·51	No	No	No	No	No	No
Ukraine	41·4	0·54	Yes	Yes	Yes	Yes	Yes	No
United Arab Emirates	10·0	0·13	Yes	Yes	Yes	Yes	Yes	No
United Kingdom	68·2	0·89	Yes	Yes	Yes	Yes	Yes	Yes
United Republic of Tanzania	59·7	0·78	Yes	No	No	No	No	No
United States of America	332·9	4·35	Yes	Yes	Yes	Yes	Yes	Yes
Uruguay	3·5	0·05	Yes	Yes	Yes	Yes	No	No
Uzbekistan	29·9	0·39	No	No	No	No	No	No
Vanuatu	0·3	<0·01	No	No	No	No	No	No
Venezuela (Bolivarian Republic of)	28·9	0·38	Yes	No	No	No	No	No
Viet Nam	93·4	1·22	Yes	Yes	Yes	No	Yes	No
Yemen	25·0	0·33	No	No	No	No	No	No
Zambia	15·0	0·20	No	No	No	No	No	No

Zimbabwe	14·6	0·19	No	No	No	No	No	No

Note: A country is classified as "Yes" for a specific organ if it reported at least one transplant procedure between 2021 and 2023. "Population (millions)" is rounded to one decimal places; values below 0.1 million are shown as "<0.1". "Global Pop. Share (%)" represents the proportion of each country's population relative to the total population of all countries included; values below 0.01% are shown as "<0.01".

Appendix 1 Table S16: Change in national transplant capacity for solid organs between 2008–2010 and 2021–2023, by organ type

Country	Kidney Status	Liver Status	Heart Status	Lung Status	Pancreas Status	SB Status
Afghanistan	↑	–	–	–	–	–
Albania	–	–	–	–	–	–
Algeria	–	–	–	–	–	–
Andorra	–	–	–	–	–	–
Angola	–	–	–	–	–	–
Antigua and Barbuda	–	–	–	–	–	–
Argentina	–	–	–	–	–	–
Armenia	–	↑	–	–	–	–
Australia	–	–	–	–	–	–
Austria	–	–	–	–	–	↑
Azerbaijan	–	–	–	–	–	–
Bahamas	–	–	–	–	–	–
Bahrain	–	–	–	–	–	–
Bangladesh	–	–	–	–	–	–
Barbados	–	–	–	–	–	–
Belarus	–	–	–	↑	–	–
Belgium	–	–	–	–	–	↑
Belize	–	–	–	–	–	–
Benin	–	–	–	–	–	–
Bhutan	–	–	–	–	–	–
Bolivia (Plurinational State of)	–	↑	–	–	–	–
Bosnia and Herzegovina	–	↑	–	–	–	–
Botswana	–	–	–	–	–	–
Brazil	–	–	–	–	–	↑
Brunei Darussalam	–	–	–	–	–	–

Bulgaria	–	–	–	–	–	–
Burkina Faso	–	–	–	–	–	–
Burundi	–	–	–	–	–	–
Cambodia	–	–	–	–	–	–
Cameroon	–	–	–	–	–	–
Canada	–	–	–	–	–	–
Cape Verde	–	–	–	–	–	–
Central African Republic	–	–	–	–	–	–
Chad	–	–	–	–	–	–
Chile	–	–	–	–	↑	–
China	–	–	–	–	↑	↑
Colombia	–	–	–	–	–	–
Comoros	–	–	–	–	–	–
Congo	–	–	–	–	–	–
Cook Islands	–	–	–	–	–	–
Costa Rica	–	–	–	↑	–	–
Croatia	–	–	–	↑	–	↑
Cuba	–	–	↓	–	↓	–
Cyprus	–	–	–	–	–	–
Czech Republic	–	–	–	–	–	↑
Côte d’Ivoire	–	–	–	–	–	–
Democratic People's Republic of Korea	–	–	–	–	–	–
Democratic Republic of The Congo	–	–	–	–	–	–
Denmark	–	–	–	–	↑	–
Djibouti	–	–	–	–	–	–
Dominica	–	–	–	–	–	–
Dominican Republic	–	–	–	–	–	–
Ecuador	–	–	–	–	–	–
Egypt	↓	↓	–	–	–	–
El Salvador	–	–	–	–	–	–
Equatorial Guinea	–	–	–	–	–	–
Eritrea	–	–	–	–	–	–

Estonia	–	–	–	–	↑	–
Eswatini	–	–	–	–	–	–
Ethiopia	↑	–	–	–	–	–
Fiji	–	–	–	–	–	–
Finland	–	–	–	–	↑	–
France	–	–	–	–	–	–
Gabon	–	–	–	–	–	–
Gambia	–	–	–	–	–	–
Georgia	–	↑	–	–	–	–
Germany	–	–	–	–	–	–
Ghana	↓	–	–	–	–	–
Greece	–	–	–	–	↓	–
Grenada	–	–	–	–	–	–
Guatemala	–	–	–	–	–	–
Guinea	–	–	–	–	–	–
Guinea-Bissau	–	–	–	–	–	–
Guyana	–	–	–	–	–	–
Haiti	–	–	–	–	–	–
Honduras	–	–	–	–	–	–
Hungary	–	–	–	↑	–	–
Iceland	–	–	–	–	–	–
India	–	–	–	↑	↑	–
Indonesia	↓	↓	–	–	–	–
Iran (Islamic Republic of)	–	–	–	–	–	–
Iraq	–	–	–	–	–	–
Ireland	–	–	–	–	–	–
Israel	–	–	–	–	–	↓
Italy	–	–	–	–	–	–
Jamaica	↑	–	–	–	–	–
Japan	–	–	–	–	–	–
Jordan	–	–	–	–	–	–
Kazakhstan	–	–	–	↑	↓	–

Kenya	–	–	–	–	–	–
Kiribati	–	–	–	–	–	–
Kuwait	–	↓	↑	–	–	–
Kyrgyzstan	–	↑	–	–	–	–
Lao People's Democratic Republic	–	–	–	–	–	–
Latvia	–	↑	–	–	↓	–
Lebanon	–	–	↑	↑	–	–
Lesotho	–	–	–	–	–	–
Liberia	–	–	–	–	–	–
Libyan Arab Jamahiriya	–	↓	–	–	–	–
Lithuania	–	–	–	–	–	–
Luxembourg	↓	↓	↓	↓	–	↓
Madagascar	–	–	–	–	–	–
Malawi	–	–	–	–	–	–
Malaysia	–	–	↓	↓	–	–
Maldives	–	–	–	–	–	–
Mali	–	–	–	–	–	–
Malta	–	↓	↓	–	–	–
Marshall Islands	–	–	–	–	–	–
Mauritania	–	–	–	–	–	–
Mauritius	↓	–	–	–	–	–
Mexico	–	–	–	–	–	–
Micronesia (Federated States of)	–	–	–	–	–	–
Monaco	–	–	–	–	–	–
Mongolia	–	↑	–	–	–	–
Montenegro	–	–	–	–	–	–
Morocco	–	–	–	–	–	–
Mozambique	–	–	–	–	–	–
Myanmar	↓	↓	–	–	–	–
Namibia	–	–	–	–	–	–
Nauru	–	–	–	–	–	–
Nepal	↓	–	–	–	–	–

Netherlands	–	–	–	–	–	–
New Zealand	–	–	–	–	–	–
Nicaragua	–	–	–	–	–	–
Niger	–	–	–	–	–	–
Nigeria	–	–	–	–	–	–
Niue	–	–	–	–	–	–
Norway	–	–	–	–	–	–
Oman	–	↑	–	–	–	–
Pakistan	–	↑	–	–	–	–
Palau	–	–	–	–	–	–
Panama	–	↑	↑	–	–	–
Papua New Guinea	–	–	–	–	–	–
Paraguay	–	↑	–	–	–	↓
Peru	–	–	–	–	↑	–
Philippines	–	↑	–	–	–	–
Poland	–	–	–	–	–	–
Portugal	–	–	–	–	–	–
Qatar	–	↑	–	↑	–	–
Republic of Korea	–	–	–	–	–	–
Republic of Moldova	↑	↑	–	–	–	–
Republic of North Macedonia	–	↑	↑	–	–	–
Romania	–	–	–	↑	–	–
Russian Federation	–	–	–	–	↑	↑
Rwanda	–	–	–	–	–	–
Saint Kitts and Nevis	–	–	–	–	–	–
Saint Lucia	–	–	–	–	–	–
Saint Vincent and the Grenadines	–	–	–	–	–	–
Samoa	–	–	–	–	–	–
San Marino	–	–	–	–	–	–
Sao Tome and Principe	–	–	–	–	–	–
Saudi Arabia	–	–	–	–	–	↑
Senegal	–	–	–	–	–	–

Serbia	–	–	–	–	–	–
Seychelles	–	–	–	–	–	–
Sierra Leone	–	–	–	–	–	–
Singapore	–	–	–	↑	↑	–
Slovakia	–	–	–	–	–	–
Slovenia	–	–	–	↑	↑	–
Solomon Islands	–	–	–	–	–	–
Somalia	–	–	–	–	–	–
South Africa	–	–	–	–	–	–
South Sudan	–	–	–	–	–	–
Spain	–	–	–	–	–	–
Sri Lanka	–	↑	↑	↑	–	–
Sudan	–	–	–	–	–	–
Suriname	–	–	–	–	–	–
Sweden	–	–	–	–	↑	↑
Switzerland	–	–	–	–	–	–
Syrian Arab Republic	–	–	–	–	–	–
Tajikistan	–	–	–	–	–	–
Thailand	–	–	–	↑	↑	↑
Timor-Leste	–	–	–	–	–	–
Togo	–	–	–	–	–	–
Tonga	–	–	–	–	–	–
Trinidad and Tobago	–	–	–	–	–	–
Tunisia	–	–	↑	–	–	–
Turkmenistan	–	–	–	–	–	–
Tuvalu	–	–	–	–	–	–
Türkiye	–	–	–	–	–	–
Uganda	–	–	–	–	–	–
Ukraine	–	–	↑	↑	↑	–
United Arab Emirates	–	↑	↑	↑	↑	–
United Kingdom	–	–	–	–	–	–
United Republic of Tanzania	–	–	–	–	–	–

United States of America	–	–	–	–	–	–
Uruguay	–	–	–	–	↓	–
Uzbekistan	–	–	–	–	–	–
Vanuatu	–	–	–	–	–	–
Venezuela (Bolivarian Republic of)	–	↓	–	–	–	–
Viet Nam	–	–	↑	–	↑	↓
Yemen	–	–	–	–	–	–
Zambia	–	–	–	–	–	–
Zimbabwe	–	–	–	–	–	–

↑ indicates a gain of transplant capacity for the respective organ, defined as any transplant activity reported in 2021–2023 but not in 2008–2010. ↓ indicates a loss of capacity, defined as activity reported in 2008–2010 but not in 2021–2023. – indicates no change (either capacity was present or absent during both time windows). Capacity was defined as ≥1 transplant/year in any year within each 3-year period. Each organ type was assessed independently. SB = small bowel (ie, intestinal) transplantation.

Appendix 1 Table S17: Trends in kidney transplant number and rate by country, 2008–2023, and percent change (PC) and estimated annual percent change (EAPC, 95% CI)

	2008		2023		2008-2023	
**Country**	**Kidney transplants**	**PMP**	**Kidney transplants**	**PMP**	**PC(%)**	**EAPC (%, 95% CI)**
Afghanistan	0	0·0	459	10·9	–	–
Albania	1	0·3	26	9·3	2500·00	16·69 (10·64 to 23·07)
Algeria	112	3·3	91	2·1	-18·75	-1·56 (-5·67 to 2·73)
Argentina	960	24·1	1585	34·6	65·10	1·02 (-0·64 to 2·70)
Armenia	8	2·7	21	7·5	162·50	7·30 (4·25 to 10·43)
Australia	776	37·0	1086	41·1	39·95	0·31 (-0·75 to 1·38)
Austria	361	43·0	327	36·3	-9·42	-2·13 (-3·21 to -1·05)
Azerbaijan	52	6·1	31	3·0	-40·38	-3·61 (-6·88 to -0·24)
Bangladesh	28	0·2	319	1·8	1039·29	20·54 (17·20 to 23·97)
Barbados	1	3·3	1	3·3	0·00	0·00 (0·00 to 0·00)
Belarus	175	18·2	355	37·4	102·86	5·87 (3·87 to 7·91)
Belgium	487	46·4	529	45·2	8·62	-1·28 (-2·43 to -0·12)
Bolivia (Plurinational State of)	79	8·1	62	5·0	-21·52	-6·33 (-11·43 to -0·94)
Bosnia and Herzegovina	15	3·9	14	4·4	-6·67	-3·96 (-8·27 to 0·55)
Brazil	3780	19·5	5811	26·9	53·73	0·66 (-0·61 to 1·95)

Bulgaria	19	2·5	27	4·0	42·11	-1·14 (-6·92 to 5·00)
Canada	1204	36·3	2078	53·6	72·59	2·27 (1·46 to 3·08)
Chile	206	12·3	378	19·3	83·50	2·12 (-0·01 to 4·29)
China	6100	4·6	14968	10·4	145·38	6·95 (5·08 to 8·85)
Colombia	705	15·1	965	18·5	36·88	-0·79 (-2·59 to 1·05)
Costa Rica	130	28·9	68	13·1	-47·69	-8·27 (-10·92 to -5·54)
Croatia	158	34·4	141	35·3	-10·76	-3·25 (-5·35 to -1·10)
Cuba	144	12·7	13	1·2	-90·97	-11·17 (-18·46 to -3·24)
Cyprus	58	64·4	35	26·9	-39·66	-7·71 (-10·61 to -4·71)
Czech Republic	334	32·8	488	46·5	46·11	2·17 (1·28 to 3·06)
Denmark	196	35·6	298	50·5	52·04	1·26 (0·44 to 2·09)
Dominican Republic	102	10·3	79	7·0	-22·55	-1·37 (-4·83 to 2·22)
Ecuador	58	4·3	182	10·0	213·79	3·51 (-0·75 to 7·94)
Egypt	1200	15·6	0	0·0	-100·00	–
El Salvador	29	4·1	47	7·3	62·07	-1·85 (-5·79 to 2·26)
Estonia	57	43·9	51	39·2	-10·53	-0·85 (-2·90 to 1·25)
Ethiopia	0	0·0	8	<0·1	–	-12·47 (-27·55 to 5·75)
Finland	150	28·3	321	58·4	114·00	3·84 (2·87 to 4·82)
France	2885	46·6	3525	54·4	22·18	0·80 (-0·22 to 1·83)
Georgia	7	1·6	31	8·4	342·86	8·67 (3·75 to 13·38)
Germany	2753	33·4	2122	25·5	-22·92	-2·72 (-3·53 to -1·89)
Ghana	1	<0·1	0	0·0	-100·00	–
Greece	237	21·2	241	23·4	1·69	1·58 (-0·98 to 4·21)
Guatemala	85	6·2	111	6·1	30·59	-8·59 (-15·46 to -1·16)
Guyana	0	0·0	8	10·0	–	–
Honduras	4	0·6	4	0·4	0·00	-3·72 (-9·53 to 2·46)
Hungary	259	25·9	265	26·0	2·32	-1·04 (-2·91 to 0·88)
Iceland	5	16·7	10	25·0	100·00	2·57 (0·11 to 5·09)
India	5600	4·7	13426	9·4	139·75	5·08 (2·86 to 7·34)
Indonesia	494	2·1	0	0·0	-100·00	–
Iran (Islamic Republic of)	1926	26·7	2431	28·3	26·22	-1·85 (-3·78 to 0·12)
Ireland	146	33·2	189	37·1	29·45	-1·36 (-2·67 to -0·03)

Israel	142	20·3	451	49·0	217·61	7·17 (5·59 to 8·77)
Italy	1656	28·1	2244	38·1	35·51	1·85 (1·13 to 2·57)
Jamaica	0	0·0	0	0·0	–	–
Japan	1201	9·4	1792	14·5	49·21	2·48 (1·59 to 3·37)
Jordan	190	31·2	223	19·7	17·37	-4·76 (-6·84 to -2·64)
Kazakhstan	150	9·7	180	9·2	20·00	-2·01 (-4·65 to 0·70)
Kenya	120	3·1	53	1·0	-55·83	3·00 (-5·29 to 12·02)
Kuwait	76	26·2	140	32·6	84·21	-0·85 (-2·98 to 1·33)
Kyrgyzstan	3	0·6	20	3·4	566·67	18·39 (12·33 to 24·77)
Latvia	54	23·5	46	25·6	-14·81	-2·86 (-4·99 to -0·68)
Lebanon	108	26·3	111	18·2	2·78	-1·24 (-3·19 to 0·75)
Libyan Arab Jamahiriya	55	8·7	45	6·5	-18·18	-8·62 (-20·50 to 5·05)
Lithuania	51	15.0	103	38·2	101·96	4·12 (2·13 to 6·14)
Luxembourg	3	6·0	0	0·0	-100·00	–
Malaysia	47	1·7	279	8·1	493·62	6·85 (3·24 to 10·58)
Malta	12	30·0	5	10·0	-58·33	-5·43 (-11·13 to 0·63)
Mauritius	0	0·0	0	0·0	–	–
Mexico	2260	21·0	3082	24·0	36·37	-1·58 (-4·66 to 1·60)
Mongolia	4	1·5	40	11·8	900·00	11·35 (3·75 to 19·51)
Montenegro	10	16·7	2	3·3	-80·00	-13·95 (-17·29 to -10·48)
Morocco	10	0·3	12	0·3	20·00	-0·16 (-7·54 to 7·82)
Myanmar	38	0·8	0	0·0	-100·00	–
Nepal	21	0·7	0	0·0	-100·00	–
Netherlands	763	46·2	1020	58·0	33·68	0·74 (-0·09 to 1·58)
New Zealand	121	28·8	175	33·7	44·63	3·07 (1·62 to 4·55)
Nicaragua	0	0·0	3	0·5	–	-9·94 (-14·88 to -4·73)
Nigeria	14	<0·1	253	1·1	1707·14	17·87 (13·50 to 22·42)
Norway	278	59·2	237	43·1	-14·75	-2·62 (-3·21 to -2·03)
Oman	15	5·6	19	4·1	26·67	-5·62 (-10·64 to -0·32)
Pakistan	775	4·6	1324	5·5	70·84	-0·58 (-7·25 to 6·57)
Panama	24	7·1	35	7·8	45·83	-6·65 (-11·63 to -1·40)
Paraguay	27	4·4	39	5·7	44·44	-2·00 (-8·65 to 5·14)

Peru	0	0·0	171	5·0	–	-6·38 (-11·28 to -1·20)
Philippines	1147	12·8	610	5·2	-46·82	-4·28 (-8·98 to 0·67)
Poland	810	21·3	1073	26·2	32·47	-0·50 (-2·08 to 1·10)
Portugal	524	49·0	547	53·6	4·39	-0·34 (-1·52 to 0·85)
Qatar	4	4·4	74	27·4	1750·00	13·72 (8·32 to 19·39)
Republic of Korea	949	19·6	2070	40·0	118·12	4·46 (2·96 to 5·98)
Republic of Moldova	0	0.0	1	0·3	–	-4·60 (-18·27 to 11·35)
Republic of North Macedonia	12	6.0	22	10·5	83·33	2·06 (-3·77 to 8·24)
Romania	227	10·7	219	11·0	-3·52	-0·16 (-2·28 to 2·00)
Russian Federation	527	3·7	1124	7·7	113·28	4·10 (1·90 to 6·34)
Saudi Arabia	394	15·6	1435	38·9	264·21	4·87 (2·78 to 7·01)
Serbia	104	10·5	51	7·2	-50·96	-13·67 (-20·24 to -6·56)
Singapore	129	28·7	88	14·7	-31·78	-1·75 (-4·63 to 1·22)
Slovakia	166	30·7	130	22·4	-21·69	-0·94 (-2·70 to 0·85)
Slovenia	52	26.0	53	25·2	1·92	-0·89 (-2·39 to 0·64)
South Africa	250	5·1	230	4·0	-8·00	-2·41 (-3·43 to -1·38)
Spain	2229	50·0	3690	77·7	65·55	2·77 (1·92 to 3·64)
Sri Lanka	300	15·5	252	11·5	-16·00	-1·58 (-2·30 to -0·85)
Sudan	68	1·7	205	4·6	201·47	5·59 (2·08 to 9·22)
Sweden	419	45·5	523	49·3	24·82	0·49 (-0·06 to 1·04)
Switzerland	286	38·1	400	45·5	39·86	0·83 (-0·02 to 1·69)
Syrian Arab Republic	259	12·7	348	15·0	34·36	0·95 (-1·87 to 3·85)
Tajikistan	3	0·4	25	2·9	733·33	9·70 (1·18 to 18·93)
Thailand	338	5·3	985	13·7	191·42	5·49 (3·37 to 7·65)
Trinidad and Tobago	12	9·2	4	2·7	-66·67	-6·33 (-9·88 to -2·66)
Tunisia	127	12·2	71	5·7	-44·09	-5·12 (-6·10 to -4·14)
Türkiye	1662	21·9	3452	40·2	107·70	2·25 (0·42 to 4·10)
Ukraine	119	2·6	396	10·8	232·77	5·80 (1·76 to 10·00)
United Arab Emirates	19	4·2	274	28·8	1342·11	12·60 (7·62 to 17·82)
United Kingdom	1836	30·1	3370	49·8	83·55	1·29 (-0·26 to 2·87)
United Republic of Tanzania	8	0·2	8	0·1	0·00	-2·51 (-2·76 to -2·26)
United States of America	16517	53·5	28144	82·8	70·39	3·18 (2·66 to 3·70)

Uruguay	121	35·6	138	40·6	14·05	0·98 (-0·90 to 2·90)
Venezuela (Bolivarian Republic of)	278	9·9	89	3·1	-67·99	-21·12 (-29·25 to -12·07)
Viet Nam	2	<0·1	270	2·9	13400·00	27·33 (12·00 to 44·75)

Abbreviations: PMP = per million population; PC = percent change; EAPC = estimated annual percent change; CI = confidence interval.
Note:1.PC was calculated as: ((2023 total transplants − 2008 total transplants) / 2008 total transplants) × 100%. 2.EAPC and 95% CIs were derived from a linear regression model fitted to the natural logarithm of PMP across years (log[PMP] ~ year). 3.A positive EAPC with a 95% CI that does not include 0 indicates a significant increasing trend; a negative EAPC with CI not crossing 0 indicates a significant decreasing trend. 4."–" indicates values not calculable due to: a) PC: zero transplants in 2008 (denominator = 0); b) EAPC: insufficient non-zero data points or persistent zero values.

Appendix 1 Table S18: Trends in the number and rate of liver transplants by country, 2008–2023, including percent change (PC) and estimated annual percent change (EAPC) with 95% confidence intervals

	2008		2023		2008-2023	
Country	Liver transplants	PMP	Liver transplants	PMP	PC(%)	EAPC (%, 95% CI)
Albania	0	0·0	0	0·0	–	–
Algeria	3	<0·1	2	<0·1	-33·33	-3·49 (-11·18 to 4·88)
Argentina	280	7·0	442	9·7	57·86	1·85 (0·47 to 3·25)
Armenia	0	0·0	5	1·8	–	–
Australia	195	9·3	287	10·9	47·18	1·23 (0·12 to 2·35)
Austria	116	13·8	123	13·7	6·03	0·69 (-0·49 to 1·88)
Azerbaijan	31	3·7	10	1·0	-67·74	-9·00 (-10·39 to -7·59)
Belarus	43	4·5	83	8·7	93·02	6·22 (4·94 to 7·52)
Belgium	230	21·9	341	29·2	48·26	0·46 (-0·57 to 1·49)
Bolivia (Plurinational State of)	0	0·0	0	0·0	–	–
Bosnia and Herzegovina	0	0·0	0	0·0	–	-18·13 (-31·65 to -1·94)
Brazil	1053	5·4	2284	10·6	116·90	3·58 (2·48 to 4·70)
Bulgaria	9	1·2	12	1·8	33·33	1·44 (-3·30 to 6·41)
Canada	545	16·4	669	17·2	22·75	0·81 (0·16 to 1·47)
Chile	74	4·4	188	9·6	154·05	5·17 (3·24 to 7·13)
China	1990	1·5	6896	4·8	246·53	10·00 (7·76 to 12·28)
Colombia	198	4·2	307	5·9	55·05	0·79 (-0·57 to 2·17)
Costa Rica	16	3·6	21	4·0	31·25	3·74 (0·05 to 7·56)
Croatia	65	14·1	108	27·0	66·15	2·24 (-0·32 to 4·87)

Cuba	28	2·5	0	0·0	-100·00	-16·39 (-23·28 to -8·88)
Czech Republic	97	9·5	183	17·4	88·66	5·37 (3·41 to 7·36)
Denmark	44	8·0	63	10·7	43·18	1·45 (0·00 to 2·93)
Dominican Republic	1	0·1	2	0·2	100·00	-6·03 (-12·65 to 1·10)
Ecuador	1	<0·1	35	1·9	3400·00	14·18 (3·99 to 25·38)
Egypt	80	1·0	0	0·0	-100·00	–
Estonia	2	1·5	17	13·1	750·00	7·80 (2·74 to 13·11)
Finland	47	8·9	78	14·2	65·96	2·78 (1·72 to 3·86)
France	1011	16·3	1342	20·7	32·74	1·05 (0·30 to 1·81)
Georgia	0	0·0	28	7·6	–	21·67 (11·30 to 33·00)
Germany	1122	13·6	868	10·4	-22·64	-3·12 (-3·88 to -2·36)
Greece	58	5·2	40	3·9	-31·03	-1·05 (-4·06 to 2·05)
Hungary	36	3·6	70	7·0	94·44	4·60 (1·91 to 7·37)
India	250	0·2	4491	3·1	1696·40	17·00 (13·66 to 20·43)
Indonesia	2	<0·1	0	0·0	-100·00	–
Iran (Islamic Republic of)	185	2·6	893	10·4	382·70	9·06 (5·99 to 12·22)
Ireland	58	13·2	46	9·0	-20·69	-2·13 (-4·28 to 0·06)
Israel	56	8·0	103	11·2	83·93	3·56 (1·97 to 5·18)
Italy	1015	17·2	1701	28·9	67·59	3·09 (2·22 to 3·96)
Jamaica	2	0·7	2	0·7	0·00	0·15 (-0·87 to 1·17)
Japan	476	3·7	470	3·8	-1·26	-0·77 (-1·51 to -0·03)
Jordan	20	3·3	1	<0·1	-95·00	-17·47 (-21·84 to -12·86)
Kazakhstan	15	1·0	50	2·6	233·33	7·49 (3·26 to 11·90)
Kuwait	1	0·3	0	0.0	-100·00	–
Kyrgyzstan	0	0·0	2	0·3	–	0·00 (0·00 to 0·00)
Latvia	0	0·0	1	0·6	–	3·37 (-11·21 to 20·34)
Lebanon	0	0·0	9	1·5	–	17·21 (10·09 to 24·80)
Libyan Arab Jamahiriya	2	0·3	0	0.0	-100·00	–
Lithuania	6	1·8	30	11·1	400·00	10·42 (7·64 to 13·27)
Luxembourg	0	0·0	0	0.0	–	–
Malaysia	5	0·2	29	0·9	480·00	9·81 (0·06 to 20·51)
Malta	9	22·5	0	0.0	-100·00	–

Mexico	96	0·9	298	2·3	210·42	5·07 (1·50 to 8·77)
Mongolia	0	0·0	101	29·7	–	28·29 (21·36 to 35·61)
Montenegro	0	0·0	0	0·0	–	–
Morocco	0	0·0	0	0·0	–	–
Myanmar	1	<0·1	0	0·0	-100·00	–
Netherlands	131	7·9	207	11·8	58·02	3·02 (2·41 to 3·63)
New Zealand	24	5·7	55	10·6	129·17	3·62 (1·90 to 5·36)
Norway	79	16·8	92	16·7	16·46	-0·52 (-1·38 to 0·35)
Oman	0	0·0	11	2·4	–	–
Pakistan	0	0·0	526	2·2	–	8·93 (-5·59 to 25·69)
Panama	0	0·0	6	1·3	–	-1·13 (-7·59 to 5·77)
Paraguay	0	0·0	2	0·3	–	-0·84 (-19·10 to 21·53)
Peru	13	0·5	43	1·3	230·77	3·64 (-0·20 to 7·63)
Philippines	0	0·0	3	<0·1	–	-1·17 (-20·83 to 23·37)
Poland	245	6·5	550	13·4	124·49	2·90 (1·35 to 4·47)
Portugal	274	25·6	249	24·4	-9·12	-0·06 (-1·93 to 1·85)
Qatar	0	0·0	11	4·1	–	12·71 (-0·14 to 27·21)
Republic of Korea	777	16·1	1497	28·9	92·66	3·16 (2·11 to 4·23)
Republic of Moldova	0	0·0	3	0·9	–	-4·63 (-14·85 to 6·81)
Republic of North Macedonia	0	0·0	1	0·5	–	2·08 (-5·92 to 10·75)
Romania	43	2·0	94	4·7	118·60	3·60 (-0·09 to 7·43)
Russian Federation	0	0·0	559	3·8	–	9·19 (7·59 to 10·82)
Saudi Arabia	97	3·8	499	13·5	414·43	8·06 (6·63 to 9·52)
Serbia	17	1·7	4	0·6	-76·47	-9·08 (-13·70 to -4·21)
Singapore	50	11·1	30	5·0	-40·00	-1·89 (-5·03 to 1·35)
Slovakia	12	2·2	40	6·9	233·33	3·79 (0·61 to 7·06)
Slovenia	22	11·0	23	11·0	4·55	-0·20 (-1·77 to 1·40)
South Africa	20	0·4	88	1·5	340·00	8·24 (6·13 to 10·40)
Spain	1108	24·8	1262	26·6	13·90	0·47 (-0·26 to 1·20)
Sri Lanka	0	0·0	21	1·0	–	30·66 (25·38 to 36·17)
Sweden	146	15·9	198	18·7	35·62	0·74 (-0·08 to 1·57)
Switzerland	83	11·1	146	16·6	75·90	2·56 (1·45 to 3·67)

Syrian Arab Republic	0	0·0	0	0·0	–	–
Thailand	50	0·8	156	2·2	212·00	6·09 (4·29 to 7·92)
Tunisia	4	0·4	10	0·8	150·00	8·80 (0·58 to 17·69)
Türkiye	602	8·0	1768	20·6	193·69	5·99 (4·11 to 7·92)
Ukraine	13	0·3	126	3·4	869·23	14·15 (7·71 to 20·99)
United Arab Emirates	0	0·0	100	10·5	–	50·78 (34·33 to 69·24)
United Kingdom	719	11·8	877	13·0	21·97	0·86 (-0·27 to 2·02)
United States of America	6318	20·5	10660	31·4	68·72	3·15 (2·63 to 3·68)
Uruguay	3	0·9	26	7·7	766·67	7·96 (2·98 to 13·17)
Venezuela (Bolivarian Republic of)	10	0·4	0	0·0	-100·00	–
Viet Nam	6	<0·1	8	<0·1	33·33	0·50 (-1·02 to 2·05)

Abbreviations: PMP = per million population; PC = percent change; EAPC = estimated annual percent change; CI = confidence interval.
Note:1.PC was calculated as: ((2023 total transplants − 2008 total transplants) / 2008 total transplants) × 100%. 2.EAPC and 95% CIs were derived from a linear regression model fitted to the natural logarithm of PMP across years (log[PMP] ~ year). 3.A positive EAPC with a 95% CI that does not include 0 indicates a significant increasing trend; a negative EAPC with CI not crossing 0 indicates a significant decreasing trend. 4.“–” indicates values not calculable due to: 1) PC: zero transplants in 2008 (denominator = 0); 2) EAPC: insufficient non-zero data points or persistent zero values.

Appendix 1 Table S19: Trends in the number and rate of heart transplants by country, 2008–2023, including percent change (PC) and estimated annual percent change (EAPC) with 95% confidence intervals

	2008		2023		2008-2023	
**Country**	**Heart transplants**	**PMP**	**Heart transplants**	**PMP**	**PC(%)**	**EAPC (%, 95% CI)**
Argentina	100	2·5	110	2·4	10·00	0·45 (-0·85 to 1·75)
Australia	85	4·1	129	4·9	51·76	3·47 (1·90 to 5·06)
Austria	62	7·4	64	7·1	3·23	-0·85 (-1·78 to 0·08)
Belarus	21	2·2	45	4·7	114·29	6·58 (4·14 to 9·09)
Belgium	75	7·1	49	4·2	-34·67	-2·12 (-3·87 to -0·34)
Brazil	200	1·0	429	2·0	114·50	4·95 (3·10 to 6·84)
Bulgaria	3	0·4	3	0·5	0·00	-4·15 (-9·74 to 1·79)
Canada	168	5·1	184	4·7	9·52	-0·77 (-2·13 to 0·60)
Chile	19	1·1	35	1·8	84·21	2·51 (-0·28 to 5·38)
China	130	<0·1	994	0·7	664·62	15·60 (12·92 to 18·35)
Colombia	83	1·8	99	1·9	19·28	-0·47 (-2·38 to 1·48)

Costa Rica	3	0·7	4	0·8	33·33	-1·61 (-9·74 to 7·26)
Croatia	20	4·4	48	12·0	140·00	3·23 (0·79 to 5·72)
Cuba	3	0·3	0	0·0	-100·00	–
Czech Republic	59	5·8	84	8·0	42·37	0·80 (-0·06 to 1·68)
Denmark	20	3·6	33	5·6	65·00	1·33 (-0·42 to 3·11)
Dominican Republic	0	0·0	0	0·0	–	–
Ecuador	1	<0·1	22	1·2	2100·00	16·42 (8·70 to 24·68)
Finland	21	4·0	19	3·5	-9·52	1·34 (-1·62 to 4·38)
France	379	6·1	393	6·1	3·69	0·11 (-0·77 to 0·98)
Germany	382	4·6	330	4·0	-13·61	-0·93 (-2·00 to 0·16)
Greece	16	1·4	14	1·4	-12·50	2·76 (-1·53 to 7·23)
Hungary	22	2·2	52	5·1	136·36	6·70 (2·53 to 11·03)
India	5	<0·1	221	0·2	4320·00	29·90 (21·07 to 39·38)
Iran (Islamic Republic of)	52	0·7	130	1·5	150·00	4·59 (1·97 to 7·27)
Ireland	4	0·9	5	1·0	25·00	2·69 (-3·09 to 8·81)
Israel	20	2·9	33	3·6	65·00	2·92 (0·11 to 5·80)
Italy	326	5·5	370	6·3	13·50	-0·47 (-2·23 to 1·33)
Japan	11	<0·1	115	0·9	945·45	16·92 (11·92 to 22·14)
Jordan	0	0·0	0	0·0	–	–
Kazakhstan	2	0·1	5	0·3	150·00	4·76 (-4·37 to 14·77)
Kuwait	0	0·0	1	0·2	–	–
Latvia	0	0·0	3	1·7	–	4·39 (-1·65 to 10·79)
Lebanon	0	0·0	2	0·3	–	-9·21 (-14·25 to -3·86)
Lithuania	5	1·5	12	4·4	140·00	3·59 (-0·31 to 7·63)
Luxembourg	0	0·0	0	0·0	–	–
Malaysia	0	0·0	0	0·0	–	–
Malta	1	2·5	0	0·0	-100·00	–
Mexico	14	0·1	40	0·3	185·71	1·73 (-3·37 to 7·11)
Morocco	0	0·0	0	0·0	–	–
Netherlands	32	1·9	74	4·2	131·25	1·89 (-0·11 to 3·93)
New Zealand	10	2·4	16	3·1	60·00	2·07 (-0·32 to 4·52)
Norway	39	8·3	32	5·8	-17·95	-1·82 (-3·77 to 0·18)

Panama	0	0·0	0	0·0	–	-1·50 (-1·91 to -1·09)
Paraguay	1	0·2	5	0·7	400·00	11·53 (7·97 to 15·21)
Peru	0	0·0	8	0·2	–	-0·76 (-7·68 to 6·67)
Poland	61	1·6	182	4·4	198·36	7·70 (6·31 to 9·11)
Portugal	42	3·9	52	5·1	23·81	-0·43 (-2·59 to 1·77)
Republic of Korea	50	1·0	245	4·7	390·00	8·78 (6·67 to 10·93)
Republic of North Macedonia	0	0·0	2	1·0	–	–
Romania	6	0·3	11	0·6	83·33	2·04 (-5·08 to 9·69)
Russian Federation	0	0·0	251	1·7	–	7·89 (5·20 to 10·65)
Saudi Arabia	19	0·8	55	1·5	189·47	4·83 (2·95 to 6·75)
Serbia	4	0·4	5	0·7	25·00	-0·55 (-7·87 to 7·34)
Singapore	3	0·7	5	0·8	66·67	-1·21 (-5·75 to 3·55)
Slovakia	26	4·8	18	3·1	-30·77	-0·94 (-2·89 to 1·06)
Slovenia	6	3·0	21	10·0	250·00	3·32 (-0·77 to 7·58)
South Africa	25	0·5	42	0·7	68·00	3·37 (0·77 to 6·05)
Spain	292	6·6	325	6·8	11·30	1·24 (0·32 to 2·16)
Sri Lanka	0	0·0	1	<0·1	–	–
Sweden	45	4·9	69	6·5	53·33	0·73 (-0·40 to 1·87)
Switzerland	29	3·9	58	6·6	100·00	0·78 (-1·20 to 2·79)
Thailand	5	<0·1	31	0·4	520·00	12·76 (9·31 to 16·32)
Tunisia	0	0·0	7	0·6	–	22·11 (14·61 to 30·10)
Türkiye	51	0·7	40	0·5	-21·57	-6·35 (-10·68 to -1·81)
Ukraine	0	0·0	62	1·7	–	67·00 (56·42 to 78·29)
United Arab Emirates	0	0·0	6	0·6	–	15·95 (-2·73 to 38·23)
United Kingdom	127	2·1	238	3·5	87·40	2·37 (1·04 to 3·73)
United States of America	2163	7·0	4599	13·5	112·62	4·60 (4·24 to 4·95)
Uruguay	11	3·2	9	2·7	-18·18	1·49 (-1·97 to 5·09)
Viet Nam	0	0·0	2	<0·1	–	1·97 (-0·53 to 4·53)

Abbreviations: PMP = per million population; PC = percent change; EAPC = estimated annual percent change; CI = confidence interval.
Note:1.PC was calculated as: ((2023 total transplants − 2008 total transplants) / 2008 total transplants) × 100%. 2.EAPC and 95% CIs were derived from a linear regression model fitted to the natural logarithm of PMP across years (log[PMP] ~ year). 3.A positive EAPC with a 95% CI that does not include 0 indicates a significant increasing trend; a negative EAPC with CI not crossing 0 indicates a significant decreasing trend. 4."–" indicates values not calculable due to: 1) PC: zero transplants in 2008 (denominator = 0); 2) EAPC: insufficient non-zero data points or persistent zero values.

Appendix 1 Table S20: Trends in the number and rate of lung transplants by country, 2008–2023, including percent change (PC) and estimated annual percent change (EAPC) with 95% confidence intervals

	2008		2023		2008-2023	
**Country**	**Lung transplants**	**PMP**	**Lung transplants**	**PMP**	**PC(%)**	**EAPC (%, 95% CI)**
Argentina	32	0·8	28	0·6	-12·50	-1·42 (-3·64 to 0·85)
Australia	115	5·5	164	6·2	42·61	0·75 (-1·03 to 2·56)
Austria	119	14·2	124	13·8	4·20	-1·12 (-2·12 to -0·10)
Belarus	0	0·0	7	0·7	–	22·56 (11·23 to 35·04)
Belgium	82	7·8	116	9·9	41·46	-0·41 (-1·89 to 1·10)
Brazil	53	0·3	81	0·4	52·83	4·15 (0·52 to 7·92)
Bulgaria	0	0·0	0	0·0	–	–
Canada	135	4·1	450	11·6	233·33	5·91 (4·28 to 7·57)
Chile	9	0·5	28	1·4	211·11	2·91 (-1·51 to 7·52)
China	35	<0·1	959	0·7	2640·00	31·11 (24·82 to 37·72)
Colombia	14	0·3	36	0·7	157·14	9·80 (5·18 to 14·62)
Costa Rica	0	0·0	6	1·2	–	17·23 (10·63 to 24·23)
Croatia	0	0·0	6	1·5	–	–
Czech Republic	20	2·0	67	6·4	235·00	8·87 (6·54 to 11·25)
Denmark	18	3·3	40	6·8	122·22	0·16 (-1·92 to 2·28)
Ecuador	0	0·0	0	0·0	–	–
Estonia	0	0·0	2	1·5	–	-1·61 (-8·79 to 6·13)
Finland	12	2·3	28	5·1	133·33	3·15 (0·91 to 5·45)
France	215	3·5	307	4·7	42·79	1·57 (0·06 to 3·10)
Germany	270	3·3	266	3·2	-1·48	-0·37 (-1·83 to 1·10)
Greece	3	0·3	12	1·2	300·00	9·04 (3·04 to 15·40)
Hungary	0	0·0	11	1·1	–	13·12 (-11·43 to 44·47)

India	0	0·0	197	0·1	–	21·26 (10·35 to 33·25)
Iran (Islamic Republic of)	0	0·0	9	0·1	–	-5·86 (-10·88 to -0·56)
Ireland	0	0·0	19	3·7	–	7·96 (0·06 to 16·49)
Israel	51	7·3	60	6·5	17·65	-0·14 (-2·12 to 1·88)
Italy	94	1·6	189	3·2	101·06	2·54 (1·08 to 4·02)
Japan	14	0·1	128	1·0	814·29	15·69 (10·62 to 21·01)
Kazakhstan	0	0·0	1	<0·1	–	-9·47 (-20·86 to 3·56)
Lebanon	0	0·0	1	0·2	–	-0·12 (-0·25 to 0·02)
Lithuania	2	0·6	0	0·0	-100·00	-4·35 (-9·23 to 0·80)
Malaysia	0	0·0	0	0·0	–	–
Malta	0	0·0	0	0·0	–	–
Mexico	1	<0·1	6	<0·1	500·00	16·49 (9·84 to 23·53)
Netherlands	56	3·4	109	6·2	94·64	3·36 (2·13 to 4·61)
New Zealand	14	3·3	25	4·8	78·57	3·20 (0·90 to 5·56)
Norway	30	6·4	37	6·7	23·33	-0·47 (-1·79 to 0·88)
Peru	0	0·0	1	<0·1	–	-6·27 (-13·59 to 1·66)
Poland	11	0·3	99	2·4	800·00	17·10 (15·64 to 18·58)
Portugal	4	0·4	44	4·3	1000·00	13·54 (10·28 to 16·89)
Qatar	0	0·0	3	1·1	–	–
Republic of Korea	8	0·2	202	3·9	2425·00	23·40 (19·32 to 27·61)
Romania	0	0·0	1	<0·1	–	–
Russian Federation	0	0·0	11	<0·1	–	9·87 (-0·42 to 21·23)
Saudi Arabia	1	<0·1	41	1·1	4000·00	14·52 (6·84 to 22·76)
Singapore	0	0·0	0	0·0	–	2·41 (-7·03 to 12·80)
Slovenia	0	0·0	11	5·2	–	–
South Africa	5	0·1	23	0·4	360·00	12·09 (8·67 to 15·62)
Spain	192	4·3	479	10·1	149·48	5·25 (4·34 to 6·16)
Sri Lanka	0	0·0	1	<0·1	–	–
Sweden	52	5·7	86	8·1	65·38	0·14 (-1·49 to 1·79)
Switzerland	40	5·3	71	8·1	77·50	-0·53 (-2·58 to 1·58)
Thailand	0	0·0	3	<0·1	–	1·55 (-10·00 to 14·57)
Tunisia	0	0·0	0	0·0	–	–

Türkiye	1	<0·1	15	0·2	1400·00	11·03 (0·40 to 22·79)
Ukraine	0	0·0	5	0·1	–	–
United Arab Emirates	0	0·0	16	1·7	–	41·15 (14·69 to 73·72)
United Kingdom	139	2·3	143	2·1	2·88	-3·33 (-5·75 to -0·84)
United States of America	1478	4·8	3080	9·1	108·39	3·94 (3·38 to 4·51)
Uruguay	4	1·2	1	0·3	-75·00	-0·50 (-7·92 to 7·52)

Abbreviations: PMP = per million population; PC = percent change; EAPC = estimated annual percent change; CI = confidence interval.
Note:1·PC was calculated as: ((2023 total transplants − 2008 total transplants) / 2008 total transplants) × 100%. 2.EAPC and 95% CIs were derived from a linear regression model fitted to the natural logarithm of PMP across years (log[PMP] ~ year). 3.A positive EAPC with a 95% CI that does not include 0 indicates a significant increasing trend; a negative EAPC with CI not crossing 0 indicates a significant decreasing trend. 4.“–” indicates values not calculable due to: 1) PC: zero transplants in 2008 (denominator = 0); 2) EAPC: insufficient non-zero data points or persistent zero values.

Appendix 1 Table S21: Trends in the number and rate of Pancreas transplants by country, 2008–2023, including percent change (PC) and estimated annual percent change (EAPC) with 95% confidence intervals

	2008		2023		2008-2023	
**Country**	**Pancreas transplants**	**PMP**	**Pancreas transplants**	**PMP**	**PC(%)**	**EAPC (%, 95% CI)**
Argentina	85	2·1	29	0·6	-65·88	-6·77 (-9·36 to -4·10)
Australia	32	1·5	47	1·8	46·88	1·42 (-0·59 to 3·47)
Austria	34	4·1	10	1·1	-70·59	-4·32 (-7·43 to -1·10)
Belarus	2	0·2	0	0·0	-100·00	-1·26 (-7·88 to 5·84)
Belgium	18	1·7	9	0·8	-50·00	-2·29 (-5·89 to 1·46)
Brazil	32	0·2	119	0·6	271·88	3·28 (-1·73 to 8·55)
Canada	85	2·6	66	1·7	-22·35	-4·23 (-5·90 to -2·52)
Chile	0	0·0	6	0·3	–	12·92 (2·30 to 24·64)
China	0	0·0	76	<0·1	–	–
Colombia	5	0·1	15	0·3	200·00	7·14 (1·83 to 12·73)
Croatia	14	3·0	5	1·3	-64·29	-7·15 (-12·94 to -0·98)
Cuba	1	<0·1	0	0·0	-100·00	–
Cyprus	0	0·0	0	0·0	–	–
Czech Republic	26	2·6	30	2·9	15·38	1·26 (-0·67 to 3·23)
Denmark	0	0·0	3	0·5	–	-3·97 (-15·79 to 9·51)
Dominican Republic	0	0·0	0	0·0	–	–

Ecuador	0	0·0	0	0·0	–	–
Estonia	0	0·0	4	3·1	–	11·49 (-3·66 to 29·02)
Finland	0	0·0	28	5·1	–	19·09 (8·01 to 31·30)
France	81	1·3	74	1·1	-8·64	-1·99 (-4·84 to 0·94)
Germany	134	1·6	59	0·7	-55·97	-6·66 (-8·81 to -4·46)
Greece	2	0·2	0	0·0	-100·00	–
Hungary	5	0·5	11	1·1	120·00	-2·90 (-8·03 to 2·51)
India	0	0·0	27	<0·1	–	19·77 (8·58 to 32·12)
Iran (Islamic Republic of)	13	0·2	38	0·4	192·31	4·04 (0·31 to 7·91)
Ireland	12	2·7	6	1·2	-50·00	-4·22 (-12·82 to 5·22)
Israel	11	1·6	6	0·7	-45·45	-8·13 (-12·37 to -3·69)
Italy	59	1·0	38	0·7	-35·59	-3·16 (-5·01 to -1·27)
Japan	10	<0·1	37	0·3	270·00	3·87 (-0·56 to 8·49)
Kazakhstan	1	<0·1	0	0·0	-100·00	–
Kuwait	1	0·3	2	0·5	100·00	-1·03 (-10·48 to 9·42)
Latvia	1	0·4	0	0·0	-100·00	–
Lithuania	4	1·2	2	0·7	-50·00	-4·25 (-9·80 to 1·63)
Mexico	1	<0·1	0	0·0	-100·00	3·39 (-3·73 to 11·04)
Montenegro	0	0·0	0	0·0	–	–
Netherlands	14	0·9	16	0·9	14·29	-1·00 (-4·58 to 2·72)
New Zealand	4	1·0	3	0·6	-25·00	1·27 (-2·64 to 5·34)
Norway	10	2·1	4	0·7	-60·00	-12·39 (-18·68 to -5·61)
Peru	0	0·0	0	0·0	–	5·65 (-7·09 to 20·14)
Poland	20	0·5	28	0·7	40·00	-5·22 (-11·78 to 1·83)
Portugal	14	1·3	28	2·8	100·00	3·03 (0·81 to 5·30)
Republic of Korea	18	0·4	24	0·5	33·33	0·99 (-4·37 to 6·66)
Romania	0	0·0	0	0·0	–	–
Russian Federation	0	0·0	16	0·1	–	-0·69 (-6·66 to 5·67)
Saudi Arabia	1	<0·1	18	0·5	1700·00	12·64 (1·21 to 25·35)
Singapore	0	0·0	1	0·2	–	-1·09 (-1·35 to -0·83)
Slovenia	0	0·0	0	0·0	–	-5·97 (-18·85 to 8·96)
South Africa	5	0·1	8	0·1	60·00	-0·38 (-5·83 to 5·38)

Spain	104	2·3	100	2·1	-3·85	-1·33 (-3·00 to 0·37)
Sweden	0	0·0	23	2·2	–	-7·71 (-10·80 to -4·51)
Switzerland	17	2·3	11	1·3	-35·29	-6·42 (-11·68 to -0·84)
Thailand	0	0·0	3	<0·1	–	2·14 (-5·94 to 10·90)
Türkiye	10	0·1	1	<0·1	-90·00	-17·16 (-22·43 to -11·53)
Ukraine	0	0·0	2	<0·1	–	–
United Arab Emirates	0	0·0	3	0·3	–	–
United Kingdom	216	3·5	166	2·5	-23·15	-4·99 (-6·99 to -2·95)
United States of America	1273	4·1	914	2·7	-28·20	-2·05 (-2·71 to -1·39)
Uruguay	6	1·8	0	0·0	-100·00	–
Viet Nam	0	0·0	1	<0·1	–	-0·05 (-0·11 to 0·01)

Abbreviations: PMP = per million population; PC = percent change; EAPC = estimated annual percent change; CI = confidence interval.
Note:1.PC was calculated as: ((2023 total transplants − 2008 total transplants) / 2008 total transplants) × 100%. 2.EAPC and 95% CIs were derived from a linear regression model fitted to the natural logarithm of PMP across years (log[PMP] ~ year). 3.A positive EAPC with a 95% CI that does not include 0 indicates a significant increasing trend; a negative EAPC with CI not crossing 0 indicates a significant decreasing trend. 4.“–” indicates values not calculable due to: 1) PC: zero transplants in 2008 (denominator = 0); 2) EAPC: insufficient non-zero data points or persistent zero values.

Appendix 1 Table S22: Trends in the number and rate of small bowel transplants by country, 2008–2023, including percent change (PC) and estimated annual percent change (EAPC) with 95% confidence intervals

	**2008**		**2023**		**2008-2023**	
**Country**	**Small bowel transplants**	**PMP**	**Small bowel transplants**	**PMP**	**PC(%)**	**EAPC (%, 95% CI)**
Argentina	8	0·2	2	<0·1	-75·00	-11·78 (-16·59 to -6·69)
Australia	0	0·0	1	<0·1	–	2·08 (-1·71 to 6·01)
Austria	0	0·0	0	0·0	–	–
Belgium	0	0·0	2	0·2	–	-8·46 (-11·34 to -5·49)
Brazil	0	0·0	1	<0·1	–	2·09 (-12·51 to 19·13)
Canada	4	0·1	7	0·2	75·00	9·73 (4·42 to 15·30)
China	0	0·0	12	<0·1	–	–
Colombia	0	0·0	1	<0·1	–	-13·70 (-18·36 to -8·76)
Costa Rica	0	0·0	0	0·0	–	–
Croatia	0	0·0	0	0·0	–	–
Czech Republic	0	0·0	0	0·0	–	-2·90 (-14·94 to 10·83)

Finland	0	0·0	1	0·2	–	-3·47 (-12·48 to 6·47)
France	13	0·2	1	<0·1	-92·31	-15·15 (-18·11 to -12·08)
Germany	0	0·0	1	<0·1	–	-10·15 (-15·39 to -4·58)
India	0	0·0	16	<0·1	–	17·32 (6·01 to 29·83)
Iran (Islamic Republic of)	0	0·0	9	0·1	–	0·89 (-6·38 to 8·72)
Israel	1	0·1	0	0·0	-100·00	–
Italy	3	<0·1	1	<0·1	-66·67	-10·48 (-14·68 to -6·09)
Japan	1	<0·1	3	<0·1	200·00	7·82 (2·17 to 13·78)
Luxembourg	0	0·0	0	0·0	–	–
Mexico	0	0·0	0	0·0	–	–
Netherlands	0	0·0	1	<0·1	–	1·02 (-4·40 to 6·75)
Paraguay	1	0·2	0	0·0	-100·00	–
Republic of Korea	0	0·0	0	0·0	–	-1·19 (-9·49 to 7·87)
Russian Federation	0	0·0	1	<0·1	–	-0·26 (-0·33 to -0·19)
Saudi Arabia	0	0·0	3	<0·1	–	16·25 (-5·50 to 43·00)
Spain	14	0·3	7	0·2	-50·00	-5·07 (-8·48 to -1·53)
Sweden	0	0·0	2	0·2	–	-3·12 (-6·20 to 0·05)
Switzerland	1	0·1	0	0·0	-100·00	-1·23 (-1·44 to -1·02)
Thailand	0	0·0	0	0·0	–	–
Türkiye	3	<0·1	1	<0·1	-66·67	-2·43 (-8·86 to 4·44)
United Kingdom	14	0·2	19	0·3	35·71	0·26 (-1·91 to 2·48)
United States of America	185	0·6	95	0·3	-48·65	-4·99 (-6·48 to -3·49)
Uruguay	0	0·0	0	0·0	–	–
Viet Nam	12	0·1	0	0·0	-100·00	–

Abbreviations: PMP = per million population; PC = percent change; EAPC = estimated annual percent change; CI = confidence interval.
Note:1.PC was calculated as: ((2023 total transplants − 2008 total transplants) / 2008 total transplants) × 100%. 2.EAPC and 95% CIs were derived from a linear regression model fitted to the natural logarithm of PMP across years (log[PMP] ~ year). 3.A positive EAPC with a 95% CI that does not include 0 indicates a significant increasing trend; a negative EAPC with CI not crossing 0 indicates a significant decreasing trend. 4."–" indicates values not calculable due to: 1) PC: zero transplants in 2008 (denominator = 0); 2) EAPC: insufficient non-zero data points or persistent zero values.

Appendix 1 Table S23: Trends in Missing Kidney Transplants and Coverage Rates by Country, 2008–2023

	2008		2023			

Country	Kidney Missing	Kidney Coverage	Kidney Missing	Kidney Coverage
**Global**	167899	0·29	207810	0·36
Afghanistan	995	<0·01	1305	0·26
Albania	112	0·01	91	0·22
Algeria	1102	0·09	1744	0·05
Andorra	4	<0·01	4	<0·01
Angola	618	<0·01	924	<0·01
Antigua and Barbuda	4	<0·01	4	<0·01
Argentina	448	0·68	329	0·83
Armenia	98	0·08	96	0·18
Australia	0	1·00	18	0·98
Austria	0	1·00	49	0·87
Azerbaijan	248	0·17	400	0·07
Bahamas	11	<0·01	17	<0·01
Bahrain	28	<0·01	54	<0·01
Bangladesh	5666	<0·01	6912	0·04
Barbados	10	0·09	12	0·08
Belarus	164	0·52	42	0·89
Belgium	0	1·00	0	1·00
Belize	11	<0·01	13	<0·01
Benin	328	<0·01	443	<0·01
Bhutan	25	<0·01	33	<0·01
Bolivia (Plurinational State of)	263	0·23	456	0·12
Bosnia and Herzegovina	123	0·11	120	0·10
Botswana	67	<0·01	84	<0·01
Brazil	3075	0·55	3235	0·64
Brunei Darussalam	14	<0·01	17	<0·01
Bulgaria	249	0·07	253	0·10
Burkina Faso	537	<0·01	727	<0·01
Burundi	314	<0·01	439	<0·01
Cambodia	519	<0·01	644	<0·01
Cameroon	667	<0·01	974	<0·01
Canada	0	1·00	0	1·00
Cape Verde	18	<0·01	21	<0·01
Central African Republic	155	<0·01	196	<0·01

Chad	392	<0·01	552	<0·01
Chile	387	0·35	441	0·46
China	41071	0·13	44969	0·25
Colombia	944	0·43	1213	0·44
Comoros	32	<0·01	33	<0·01
Congo	134	<0·01	192	<0·01
Cook Islands	0	<0·01	1	<0·01
Costa Rica	29	0·82	149	0·31
Croatia	4	0·97	26	0·84
Cuba	255	0·36	455	0·03
Cyprus	0	1·00	19	0·64
Czech Republic	26	0·93	0	1·00
Côte d'Ivoire	692	<0·01	869	<0·01
Democratic People's Republic of Korea	844	<0·01	1045	<0·01
Democratic Republic of The Congo	2284	<0·01	2901	<0·01
Denmark	0	1·00	0	1·00
Djibouti	28	<0·01	38	<0·01
Dominica	4	<0·01	4	<0·01
Dominican Republic	247	0·29	393	0·17
Ecuador	419	0·12	579	0·24
Egypt	1511	0·44	3825	<0·01
El Salvador	218	0·12	221	0·18
Equatorial Guinea	18	<0·01	33	<0·01
Eritrea	176	<0·01	272	<0·01
Estonia	0	1·00	3	0·94
Eswatini	39	<0·01	54	<0·01
Ethiopia	3008	<0·01	5280	<0·01
Fiji	28	<0·01	38	<0·01
Finland	37	0·80	0	1·00
France	0	1·00	0	1·00
Gabon	49	<0·01	71	<0·01
Gambia	64	<0·01	79	<0·01
Georgia	148	0·05	124	0·20
Germany	159	0·95	1360	0·61
Ghana	843	<0·01	1170	<0·01
Greece	158	0·60	190	0·56

Grenada	4	<0·01	4	<0·01
Guatemala	399	0·18	646	0·15
Guinea	339	<0·01	502	<0·01
Guinea-Bissau	60	<0·01	71	<0·01
Guyana	25	<0·01	25	0·24
Haiti	346	<0·01	439	<0·01
Honduras	250	0·02	422	0·01
Hungary	94	0·73	161	0·62
Iceland	6	0·47	7	0·60
India	36273	0·13	46289	0·22
Indonesia	7777	0·06	10567	<0·01
Iran (Islamic Republic of)	623	0·76	1164	0·68
Iraq	1041	<0·01	1455	<0·01
Ireland	9	0·94	24	0·89
Israel	105	0·57	0	1·00
Italy	423	0·80	218	0·91
Jamaica	95	<0·01	117	<0·01
Japan	3314	0·27	3362	0·35
Jordan	25	0·88	249	0·47
Kazakhstan	397	0·27	639	0·22
Kenya	1243	0·09	2250	0·02
Kiribati	4	<0·01	4	<0·01
Kuwait	26	0·74	40	0·78
Kyrgyzstan	188	0·02	227	0·08
Lao People's Democratic Republic	215	<0·01	318	<0·01
Latvia	27	0·67	29	0·61
Lebanon	37	0·75	144	0·44
Lesotho	71	<0·01	88	<0·01
Liberia	138	<0·01	184	<0·01
Libyan Arab Jamahiriya	167	0·25	243	0·16
Lithuania	69	0·42	10	0·91
Luxembourg	15	0·17	29	<0·01
Madagascar	713	<0·01	986	<0·01
Malawi	505	<0·01	702	<0·01
Malaysia	906	0·05	1155	0·19
Maldives	11	<0·01	17	<0·01

Mali	448	<0·01	736	<0·01
Malta	2	0·85	16	0·24
Marshall Islands	21	<0·01	25	<0·01
Mauritania	113	<0·01	167	<0·01
Mauritius	46	<0·01	54	<0·01
Mexico	1545	0·59	2289	0·57
Micronesia (Federated States of)	21	<0·01	4	<0·01
Monaco	1	<0·01	1	<0·01
Mongolia	91	0·04	102	0·28
Montenegro	11	0·47	23	0·08
Morocco	1105	0·01	1547	0·01
Mozambique	770	<0·01	1108	<0·01
Myanmar	1699	0·02	2245	<0·01
Namibia	74	<0·01	96	<0·01
Nauru	0	<0·01	0	<0·01
Nepal	996	0·02	1191	<0·01
Netherlands	0	1·00	0	1·00
New Zealand	27	0·82	42	0·81
Nicaragua	201	<0·01	277	0·01
Niger	519	<0·01	773	<0·01
Nigeria	5334	<0·01	9102	0·03
Niue	0	–	0	–
Norway	0	1·00	0	1·00
Oman	80	0·16	173	0·10
Pakistan	5120	0·13	8729	0·13
Palau	1	<0·01	1	<0·01
Panama	96	0·20	153	0·19
Papua New Guinea	229	<0·01	314	<0·01
Paraguay	192	0·12	249	0·14
Peru	995	<0·01	1267	0·12
Philippines	2019	0·36	4293	0·12
Poland	531	0·60	641	0·63
Portugal	0	1·00	0	1·00
Qatar	28	0·13	39	0·66
Republic of Korea	760	0·56	95	0·96
Republic of Moldova	134	<0·01	141	0·01

Republic of North Macedonia	59	0·17	66	0·25
Romania	525	0·30	613	0·26
Russian Federation	4479	0·11	4975	0·18
Rwanda	353	<0·01	506	<0·01
Saint Kitts and Nevis	0	<0·01	4	<0·01
Saint Lucia	7	<0·01	8	<0·01
Saint Vincent and the Grenadines	4	<0·01	4	<0·01
Samoa	7	<0·01	8	<0·01
San Marino	1	<0·01	1	<0·01
Sao Tome and Principe	7	<0·01	8	<0·01
Saudi Arabia	499	0·44	107	0·93
Senegal	448	<0·01	681	<0·01
Serbia	245	0·30	246	0·17
Seychelles	4	<0·01	4	<0·01
Sierra Leone	212	<0·01	259	<0·01
Singapore	30	0·81	163	0·35
Slovakia	25	0·87	112	0·54
Slovenia	19	0·74	35	0·60
Solomon Islands	18	<0·01	25	<0·01
Somalia	318	<0·01	451	<0·01
South Africa	1473	0·15	2169	0·10
South Sudan	378	<0·01	489	<0·01
Spain	0	1·00	0	1·00
Sri Lanka	385	0·44	663	0·28
Sudan	1323	0·05	1672	0·11
Suriname	18	<0·01	21	<0·01
Sweden	0	1·00	0	1·00
Switzerland	0	1·00	0	1·00
Syrian Arab Republic	461	0·36	622	0·36
Tajikistan	237	0·01	330	0·07
Thailand	1932	0·15	2016	0·33
Timor-Leste	42	<0·01	50	<0·01
Togo	240	<0·01	293	<0·01
Tonga	4	<0·01	4	<0·01
Trinidad and Tobago	34	0·26	59	0·06
Tunisia	240	0·35	452	0·14

Turkmenistan	176	<0·01	222	<0·01
Tuvalu	0	<0·01	0	<0·01
Türkiye	1014	0·62	134	0·96
Uganda	1126	<0·01	1622	<0·01
Ukraine	1501	0·07	1138	0·26
United Arab Emirates	140	0·12	123	0·69
United Kingdom	317	0·85	0	1·00
United Republic of Tanzania	1457	0·01	2487	<0·01
United States of America	0	1·00	0	1·00
Uruguay	0	1·00	4	0·97
Uzbekistan	981	<0·01	1250	<0·01
Vanuatu	7	<0·01	13	<0·01
Venezuela (Bolivarian Republic of)	714	0·28	1115	0·07
Viet Nam	3122	<0·01	3634	0·07
Yemen	815	<0·01	1045	<0·01
Zambia	431	<0·01	627	<0·01
Zimbabwe	477	<0·01	610	<0·01

Note: Transplant coverage was calculated as the ratio of actual to expected transplant numbers. The number of missing kidney transplants was estimated as the difference between actual and expected transplant numbers. Countries with coverage ≥1 were capped at 1. Countries with coverage less than 0·01 were presented as "<0·01" for clarity. Missing data are denoted as "–". Data are sorted alphabetically by country. "Global" values represent the aggregate across all included countries and territories.

Appendix 1 Table S24: Trends in Missing Liver Transplants and Coverage Rates by Country, 2008–2023

	**2008**		**2023**	
**Country**	**Liver Missing**	**Liver Coverage**	**Liver Missing**	**Liver Coverage**
**Global**	72652	0·22	81924	0·34
Afghanistan	389	<0·01	679	<0·01
Albania	44	<0·01	45	<0·01
Algeria	472	0·01	705	<0·01
Andorra	1	<0·01	2	<0·01
Angola	242	<0·01	356	<0·01
Antigua and Barbuda	1	<0·01	2	<0·01
Argentina	271	0·51	295	0·60
Armenia	41	<0·01	40	0·11

Australia	95	0·67	138	0·68
Austria	0	1·00	22	0·85
Azerbaijan	86	0·26	156	0·06
Bahamas	4	<0·01	6	<0·01
Bahrain	11	<0·01	21	<0·01
Bangladesh	2226	<0·01	2785	<0·01
Barbados	4	<0·01	5	<0·01
Belarus	89	0·32	70	0·54
Belgium	0	1·00	0	1·00
Belize	4	<0·01	5	<0·01
Benin	128	<0·01	171	<0·01
Bhutan	10	<0·01	13	<0·01
Bolivia (Plurinational State of)	134	<0·01	200	<0·01
Bosnia and Herzegovina	54	<0·01	52	<0·01
Botswana	26	<0·01	32	<0·01
Brazil	1627	0·39	1200	0·66
Brunei Darussalam	6	<0·01	6	<0·01
Bulgaria	96	0·09	96	0·11
Burkina Faso	210	<0·01	280	<0·01
Burundi	123	<0·01	169	<0·01
Cambodia	203	<0·01	248	<0·01
Cameroon	261	<0·01	375	<0·01
Canada	0	1·00	0	1·00
Cape Verde	7	<0·01	8	<0·01
Central African Republic	61	<0·01	76	<0·01
Chad	153	<0·01	213	<0·01
Chile	158	0·32	128	0·60
China	16451	0·11	16190	0·30
Colombia	446	0·31	532	0·37
Comoros	12	<0·01	13	<0·01
Congo	52	<0·01	74	<0·01
Cook Islands	0	<0·01	0	<0·01
Costa Rica	46	0·26	63	0·25
Croatia	0	1·00	0	1·00
Cuba	128	0·18	180	<0·01
Cyprus	12	<0·01	21	<0·01

Czech Republic	44	0·69	0	1·00
Côte d'Ivoire	270	<0·01	335	<0·01
Democratic People's Republic of Korea	330	<0·01	403	<0·01
Democratic Republic of The Congo	893	<0·01	1117	<0·01
Denmark	32	0·58	32	0·66
Djibouti	11	<0·01	14	<0·01
Dominica	1	<0·01	2	<0·01
Dominican Republic	136	0·01	180	0·01
Ecuador	185	0·01	258	0·12
Egypt	980	0·08	1473	<0·01
El Salvador	97	<0·01	103	<0·01
Equatorial Guinea	7	<0·01	13	<0·01
Eritrea	69	<0·01	105	<0·01
Estonia	16	0·11	4	0·81
Eswatini	15	<0·01	21	<0·01
Ethiopia	1176	<0·01	2037	<0·01
Fiji	11	<0·01	14	<0·01
Finland	26	0·64	11	0·88
France	0	1·00	0	1·00
Gabon	19	<0·01	27	<0·01
Gambia	25	<0·01	31	<0·01
Georgia	61	<0·01	32	0·47
Germany	16	0·99	473	0·65
Ghana	330	<0·01	451	<0·01
Greece	97	0·38	126	0·24
Grenada	1	<0·01	2	<0·01
Guatemala	189	<0·01	291	<0·01
Guinea	132	<0·01	193	<0·01
Guinea-Bissau	23	<0·01	27	<0·01
Guyana	10	<0·01	13	<0·01
Haiti	135	<0·01	169	<0·01
Honduras	99	<0·01	164	<0·01
Hungary	102	0·26	94	0·43
Iceland	4	<0·01	6	<0·01
India	16120	0·02	18509	0·20
Indonesia	3231	<0·01	4070	<0·01

Iran (Islamic Republic of)	811	0·19	492	0·64
Iraq	407	<0·01	560	<0·01
Ireland	3	0·96	36	0·56
Israel	41	0·58	45	0·70
Italy	0	1·00	0	1·00
Jamaica	35	0·05	43	0·04
Japan	1289	0·27	1515	0·24
Jordan	64	0·24	181	0·01
Kazakhstan	199	0·07	266	0·16
Kenya	533	<0·01	887	<0·01
Kiribati	1	<0·01	2	<0·01
Kuwait	39	0·02	69	<0·01
Kyrgyzstan	75	<0·01	93	0·02
Lao People's Democratic Republic	84	<0·01	122	<0·01
Latvia	32	<0·01	28	0·03
Lebanon	57	<0·01	89	0·09
Lesotho	28	<0·01	34	<0·01
Liberia	54	<0·01	71	<0·01
Libyan Arab Jamahiriya	85	0·02	111	<0·01
Lithuania	41	0·13	13	0·69
Luxembourg	7	<0·01	11	<0·01
Madagascar	279	<0·01	380	<0·01
Malawi	197	<0·01	270	<0·01
Malaysia	368	0·01	523	0·05
Maldives	4	<0·01	6	<0·01
Mali	175	<0·01	283	<0·01
Malta	0	1·00	8	<0·01
Marshall Islands	8	<0·01	10	<0·01
Mauritania	44	<0·01	64	<0·01
Mauritius	18	<0·01	21	<0·01
Mexico	1392	0·06	1771	0·14
Micronesia (Federated States of)	8	<0·01	2	<0·01
Monaco	0	<0·01	0	<0·01
Mongolia	37	<0·01	0	1·00
Montenegro	8	<0·01	10	<0·01
Morocco	436	<0·01	601	<0·01

Mozambique	301	<0·01	427	<0·01
Myanmar	678	<0·01	865	<0·01
Namibia	29	<0·01	37	<0·01
Nauru	0	<0·01	0	<0·01
Nepal	397	<0·01	459	<0·01
Netherlands	97	0·58	76	0·73
New Zealand	34	0·41	29	0·66
Nicaragua	79	<0·01	108	<0·01
Niger	203	<0·01	298	<0·01
Nigeria	2091	<0·01	3603	<0·01
Niue	0	–	0	–
Norway	0	1·00	0	1·00
Oman	37	<0·01	63	0·15
Pakistan	2305	<0·01	3346	0·14
Palau	0	<0·01	0	<0·01
Panama	47	<0·01	66	0·08
Papua New Guinea	90	<0·01	121	<0·01
Paraguay	86	<0·01	109	0·02
Peru	376	0·03	511	0·08
Philippines	1238	<0·01	1886	<0·01
Poland	279	0·47	110	0·83
Portugal	0	1·00	0	1·00
Qatar	12	<0·01	32	0·25
Republic of Korea	0	1·00	0	1·00
Republic of Moldova	52	<0·01	52	0·05
Republic of North Macedonia	28	<0·01	33	0·03
Romania	251	0·15	226	0·29
Russian Federation	1957	<0·01	1790	0·24
Rwanda	138	<0·01	195	<0·01
Saint Kitts and Nevis	0	<0·01	2	<0·01
Saint Lucia	3	<0·01	3	<0·01
Saint Vincent and the Grenadines	1	<0·01	2	<0·01
Samoa	3	<0·01	3	<0·01
San Marino	0	<0·01	0	<0·01
Sao Tome and Principe	3	<0·01	3	<0·01
Saudi Arabia	252	0·28	95	0·84

Senegal	175	<0·01	262	<0·01
Serbia	120	0·12	110	0·03
Seychelles	1	<0·01	2	<0·01
Sierra Leone	83	<0·01	100	<0·01
Singapore	12	0·81	67	0·31
Slovakia	63	0·16	53	0·43
Slovenia	6	0·80	11	0·68
Solomon Islands	7	<0·01	10	<0·01
Somalia	124	<0·01	174	<0·01
South Africa	653	0·03	836	0·10
South Sudan	148	<0·01	188	<0·01
Spain	0	1·00	0	1·00
Sri Lanka	268	<0·01	332	0·06
Sudan	544	<0·01	723	<0·01
Suriname	7	<0·01	8	<0·01
Sweden	0	1·00	0	1·00
Switzerland	20	0·80	0	1·00
Syrian Arab Republic	282	<0·01	374	<0·01
Tajikistan	94	<0·01	137	<0·01
Thailand	837	0·06	1000	0·13
Timor-Leste	17	<0·01	19	<0·01
Togo	94	<0·01	113	<0·01
Tonga	1	<0·01	2	<0·01
Trinidad and Tobago	18	<0·01	24	<0·01
Tunisia	140	0·03	191	0·05
Turkmenistan	69	<0·01	85	<0·01
Tuvalu	0	<0·01	0	<0·01
Türkiye	444	0·58	0	1·00
Uganda	440	<0·01	625	<0·01
Ukraine	620	0·02	465	0·21
United Arab Emirates	62	<0·01	53	0·65
United Kingdom	123	0·85	213	0·80
United Republic of Tanzania	573	<0·01	961	<0·01
United States of America	0	1·00	0	1·00
Uruguay	44	0·06	29	0·47
Uzbekistan	384	<0·01	481	<0·01

Vanuatu	3	<0·01	5	<0·01
Venezuela (Bolivarian Republic of)	378	0·03	464	<0·01
Viet Nam	1215	<0·01	1496	0·01
Yemen	319	<0·01	403	<0·01
Zambia	168	<0·01	242	<0·01
Zimbabwe	186	<0·01	235	<0·01

Note: Transplant coverage was calculated as the ratio of actual to expected transplant numbers. The number of missing kidney transplants was estimated as the difference between actual and expected transplant numbers. Countries with coverage ≥1 were capped at 1. Countries with coverage less than 0·01 were presented as "<0·01" for clarity. Missing data are denoted as "–". Data are sorted alphabetically by country. "Global" values represent the aggregate across all included countries and territories.

Appendix 1 Table S25: Trends in Missing Heart Transplants and Coverage Rates by Country, 2008–2023

	**2008**		**2023**	
**Country**	**Heart_Missing**	**Heart_Coverage**	**Heart_Missing**	**Heart_Coverage**
**Global**	22973	0·19	29698	0·26
Afghanistan	118	<0·01	219	<0·01
Albania	13	<0·01	15	<0·01
Algeria	144	<0·01	228	<0·01
Andorra	0	<0·01	1	<0·01
Angola	74	<0·01	115	<0·01
Antigua and Barbuda	0	<0·01	1	<0·01
Argentina	68	0·60	128	0·46
Armenia	13	<0·01	15	<0·01
Australia	3	0·96	8	0·94
Austria	0	1·00	0	1·00
Azerbaijan	36	<0·01	54	<0·01
Bahamas	1	<0·01	2	<0·01
Bahrain	3	<0·01	7	<0·01
Bangladesh	677	<0·01	900	<0·01
Barbados	1	<0·01	2	<0·01
Belarus	19	0·52	4	0·91
Belgium	0	1·00	12	0·81
Belize	1	<0·01	2	<0·01
Benin	39	<0·01	55	<0·01

Bhutan	3	<0·01	4	<0·01
Bolivia (Plurinational State of)	41	<0·01	64	<0·01
Bosnia and Herzegovina	16	<0·01	17	<0·01
Botswana	8	<0·01	10	<0·01
Brazil	616	0·25	696	0·38
Brunei Darussalam	2	<0·01	2	<0·01
Bulgaria	29	0·09	32	0·09
Burkina Faso	64	<0·01	90	<0·01
Burundi	37	<0·01	55	<0·01
Cambodia	62	<0·01	80	<0·01
Cameroon	79	<0·01	121	<0·01
Canada	0	1·00	18	0·91
Cape Verde	2	<0·01	3	<0·01
Central African Republic	18	<0·01	24	<0·01
Chad	47	<0·01	69	<0·01
Chile	52	0·27	67	0·34
China	5482	0·02	6462	0·13
Colombia	113	0·42	172	0·37
Comoros	4	<0·01	4	<0·01
Congo	16	<0·01	24	<0·01
Cook Islands	0	<0·01	0	<0·01
Costa Rica	16	0·16	23	0·15
Croatia	0	1·00	0	1·00
Cuba	44	0·06	58	<0·01
Cyprus	4	<0·01	7	<0·01
Czech Republic	0	1·00	0	1·00
Côte d’Ivoire	82	<0·01	108	<0·01
Democratic People's Republic of Korea	100	<0·01	130	<0·01
Democratic Republic of The Congo	272	<0·01	361	<0·01
Denmark	3	0·87	0	1·00
Djibouti	3	<0·01	5	<0·01
Dominica	0	<0·01	1	<0·01
Dominican Republic	42	<0·01	59	<0·01
Ecuador	56	0·02	73	0·23
Egypt	323	<0·01	476	<0·01
El Salvador	29	<0·01	33	<0·01

Equatorial Guinea	2	<0·01	4	<0·01
Eritrea	21	<0·01	34	<0·01
Estonia	5	<0·01	7	<0·01
Eswatini	5	<0·01	7	<0·01
Ethiopia	358	<0·01	658	<0·01
Fiji	3	<0·01	5	<0·01
Finland	1	0·94	10	0·66
France	0	1·00	0	1·00
Gabon	6	<0·01	9	<0·01
Gambia	8	<0·01	10	<0·01
Georgia	18	<0·01	19	<0·01
Germany	0	1·00	103	0·76
Ghana	100	<0·01	146	<0·01
Greece	31	0·34	40	0·26
Grenada	0	<0·01	1	<0·01
Guatemala	58	<0·01	94	<0·01
Guinea	40	<0·01	62	<0·01
Guinea-Bissau	7	<0·01	9	<0·01
Guyana	3	<0·01	4	<0·01
Haiti	41	<0·01	55	<0·01
Honduras	30	<0·01	53	<0·01
Hungary	20	0·52	1	0·98
Iceland	1	<0·01	2	<0·01
India	4977	<0·01	7208	0·03
Indonesia	984	<0·01	1315	<0·01
Iran (Islamic Republic of)	251	0·17	317	0·29
Iraq	124	<0·01	181	<0·01
Ireland	14	0·22	22	0·19
Israel	9	0·68	15	0·69
Italy	0	1·00	0	1·00
Jamaica	11	<0·01	15	<0·01
Japan	526	0·02	526	0·18
Jordan	26	<0·01	59	<0·01
Kazakhstan	63	0·03	97	0·05
Kenya	162	<0·01	287	<0·01
Kiribati	0	<0·01	1	<0·01

Kuwait	12	<0·01	21	0·04
Kyrgyzstan	23	<0·01	31	<0·01
Lao People's Democratic Republic	26	<0·01	40	<0·01
Latvia	10	<0·01	6	0·32
Lebanon	17	<0·01	30	0·06
Lesotho	8	<0·01	11	<0·01
Liberia	16	<0·01	23	<0·01
Libyan Arab Jamahiriya	26	<0·01	36	<0·01
Lithuania	9	0·35	2	0·85
Luxembourg	2	<0·01	4	<0·01
Madagascar	85	<0·01	123	<0·01
Malawi	60	<0·01	87	<0·01
Malaysia	113	<0·01	178	<0·01
Maldives	1	<0·01	2	<0·01
Mali	53	<0·01	92	<0·01
Malta	1	0·60	3	<0·01
Marshall Islands	3	<0·01	3	<0·01
Mauritania	13	<0·01	21	<0·01
Mauritius	5	<0·01	7	<0·01
Mexico	439	0·03	628	0·06
Micronesia (Federated States of)	3	<0·01	1	<0·01
Monaco	0	<0·01	0	<0·01
Mongolia	11	<0·01	18	<0·01
Montenegro	3	<0·01	3	<0·01
Morocco	133	<0·01	194	<0·01
Mozambique	92	<0·01	138	<0·01
Myanmar	207	<0·01	279	<0·01
Namibia	9	<0·01	12	<0·01
Nauru	0	<0·01	0	<0·01
Nepal	121	<0·01	148	<0·01
Netherlands	37	0·46	18	0·81
New Zealand	8	0·57	11	0·59
Nicaragua	24	<0·01	35	<0·01
Niger	62	<0·01	96	<0·01
Nigeria	636	<0·01	1164	<0·01
Niue	0	–	0	–

Norway	0	1·00	0	1·00
Oman	11	<0·01	24	<0·01
Pakistan	701	<0·01	1251	<0·01
Palau	0	<0·01	0	<0·01
Panama	14	<0·01	23	<0·01
Papua New Guinea	27	<0·01	39	<0·01
Paraguay	25	0·04	31	0·14
Peru	118	<0·01	171	0·04
Philippines	377	<0·01	610	<0·01
Poland	99	0·38	31	0·85
Portugal	3	0·93	1	0·98
Qatar	4	<0·01	14	<0·01
Republic of Korea	153	0·25	24	0·91
Republic of Moldova	16	<0·01	18	<0·01
Republic of North Macedonia	8	<0·01	9	0·18
Romania	83	0·07	92	0·11
Russian Federation	596	<0·01	508	0·33
Rwanda	42	<0·01	63	<0·01
Saint Kitts and Nevis	0	<0·01	1	<0·01
Saint Lucia	1	<0·01	1	<0·01
Saint Vincent and the Grenadines	0	<0·01	1	<0·01
Samoa	1	<0·01	1	<0·01
San Marino	0	<0·01	0	<0·01
Sao Tome and Principe	1	<0·01	1	<0·01
Saudi Arabia	87	0·18	137	0·29
Senegal	53	<0·01	85	<0·01
Serbia	38	0·10	32	0·14
Seychelles	0	<0·01	1	<0·01
Sierra Leone	25	<0·01	32	<0·01
Singapore	16	0·16	26	0·16
Slovakia	0	1·00	12	0·60
Slovenia	2	0·71	0	1·00
Solomon Islands	2	<0·01	3	<0·01
Somalia	38	<0·01	56	<0·01
South Africa	180	0·12	256	0·14
South Sudan	45	<0·01	61	<0·01

Spain	0	1·00	0	1·00
Sri Lanka	81	<0·01	113	0·01
Sudan	165	<0·01	233	<0·01
Suriname	2	<0·01	3	<0·01
Sweden	0	1·00	0	1·00
Switzerland	2	0·92	0	1·00
Syrian Arab Republic	86	<0·01	121	<0·01
Tajikistan	29	<0·01	44	<0·01
Thailand	265	0·02	342	0·08
Timor-Leste	5	<0·01	6	<0·01
Togo	29	<0·01	36	<0·01
Tonga	0	<0·01	1	<0·01
Trinidad and Tobago	5	<0·01	8	<0·01
Tunisia	44	<0·01	58	0·11
Turkmenistan	21	<0·01	28	<0·01
Tuvalu	0	<0·01	0	<0·01
Türkiye	267	0·16	406	0·09
Uganda	134	<0·01	202	<0·01
Ukraine	193	<0·01	129	0·32
United Arab Emirates	19	<0·01	43	0·12
United Kingdom	129	0·50	114	0·68
United Republic of Tanzania	174	<0·01	310	<0·01
United States of America	0	1·00	0	1·00
Uruguay	3	0·77	9	0·51
Uzbekistan	117	<0·01	155	<0·01
Vanuatu	1	<0·01	2	<0·01
Venezuela (Bolivarian Republic of)	118	<0·01	150	<0·01
Viet Nam	372	<0·01	484	<0·01
Yemen	97	<0·01	130	<0·01
Zambia	51	<0·01	78	<0·01
Zimbabwe	57	<0·01	76	<0·01

Note: Transplant coverage was calculated as the ratio of actual to expected transplant numbers. The number of missing kidney transplants was estimated as the difference between actual and expected transplant numbers. Countries with coverage ≥1 were capped at 1. Countries with coverage less than 0·01 were presented as “<0·01” for clarity. Missing data are denoted as “–”. Data are sorted alphabetically by country. “Global” values represent the aggregate across all included countries and territories.

Appendix 1 Table S26: Trends in Missing Lung Transplants and Coverage Rates by Country, 2008–2023

	2008		2023	
**Country**	**Lung_Missing**	**Lung_Coverage**	**Lung_Missing**	**Lung_Coverage**
**Global**	16234	0·17	23103	0·25
Afghanistan	82	<0·01	169	<0·01
Albania	9	<0·01	11	<0·01
Algeria	100	<0·01	176	<0·01
Andorra	0	<0·01	0	<0·01
Angola	51	<0·01	88	<0·01
Antigua and Barbuda	0	<0·01	0	<0·01
Argentina	84	0·28	155	0·15
Armenia	9	<0·01	11	<0·01
Australia	0	1·00	0	1·00
Austria	0	1·00	0	1·00
Azerbaijan	25	<0·01	41	<0·01
Bahamas	1	<0·01	2	<0·01
Bahrain	2	<0·01	5	<0·01
Bangladesh	468	<0·01	692	<0·01
Barbados	1	<0·01	1	<0·01
Belarus	28	<0·01	31	0·18
Belgium	0	1·00	0	1·00
Belize	1	<0·01	1	<0·01
Benin	27	<0·01	42	<0·01
Bhutan	2	<0·01	3	<0·01
Bolivia (Plurinational State of)	28	<0·01	50	<0·01
Bosnia and Herzegovina	11	<0·01	13	<0·01
Botswana	6	<0·01	8	<0·01
Brazil	510	0·09	785	0·09
Brunei Darussalam	1	<0·01	2	<0·01
Bulgaria	22	<0·01	27	<0·01
Burkina Faso	44	<0·01	70	<0·01
Burundi	26	<0·01	42	<0·01
Cambodia	43	<0·01	62	<0·01
Cameroon	55	<0·01	93	<0·01
Canada	0	1·00	0	1·00

Cape Verde	1	<0·01	2	<0·01
Central African Republic	13	<0·01	19	<0·01
Chad	32	<0·01	53	<0·01
Chile	40	0·18	50	0·36
China	3840	0·01	4777	0·17
Colombia	121	0·10	172	0·17
Comoros	3	<0·01	3	<0·01
Congo	11	<0·01	18	<0·01
Cook Islands	0	<0·01	0	<0·01
Costa Rica	13	<0·01	15	0·29
Croatia	13	<0·01	10	0·38
Cuba	33	<0·01	45	<0·01
Cyprus	3	<0·01	5	<0·01
Czech Republic	10	0·68	0	1·00
Côte d'Ivoire	57	<0·01	83	<0·01
Democratic People's Republic of Korea	69	<0·01	100	<0·01
Democratic Republic of The Congo	188	<0·01	278	<0·01
Denmark	0	1·00	0	1·00
Djibouti	2	<0·01	4	<0·01
Dominica	0	<0·01	0	<0·01
Dominican Republic	29	<0·01	45	<0·01
Ecuador	39	<0·01	73	<0·01
Egypt	223	<0·01	366	<0·01
El Salvador	20	<0·01	26	<0·01
Equatorial Guinea	1	<0·01	3	<0·01
Eritrea	14	<0·01	26	<0·01
Estonia	4	<0·01	3	0·38
Eswatini	3	<0·01	5	<0·01
Ethiopia	247	<0·01	506	<0·01
Fiji	2	<0·01	4	<0·01
Finland	3	0·78	0	1·00
France	0	1·00	0	1·00
Gabon	4	<0·01	7	<0·01
Gambia	5	<0·01	8	<0·01
Georgia	13	<0·01	15	<0·01
Germany	0	1·00	67	0·80

Ghana	69	<0·01	112	<0·01
Greece	29	0·09	29	0·29
Grenada	0	<0·01	0	<0·01
Guatemala	40	<0·01	72	<0·01
Guinea	28	<0·01	48	<0·01
Guinea-Bissau	5	<0·01	7	<0·01
Guyana	2	<0·01	3	<0·01
Haiti	28	<0·01	42	<0·01
Honduras	21	<0·01	41	<0·01
Hungary	29	<0·01	30	0·27
Iceland	1	<0·01	2	<0·01
India	3440	<0·01	5517	0·03
Indonesia	679	<0·01	1011	<0·01
Iran (Islamic Republic of)	209	<0·01	335	0·03
Iraq	86	<0·01	139	<0·01
Ireland	13	<0·01	1	0·93
Israel	0	1·00	0	1·00
Italy	77	0·55	47	0·80
Jamaica	8	<0·01	11	<0·01
Japan	357	0·04	365	0·26
Jordan	18	<0·01	45	<0·01
Kazakhstan	45	<0·01	77	0·01
Kenya	112	<0·01	220	<0·01
Kiribati	0	<0·01	0	<0·01
Kuwait	8	<0·01	17	<0·01
Kyrgyzstan	16	<0·01	24	<0·01
Lao People's Democratic Republic	18	<0·01	30	<0·01
Latvia	7	<0·01	7	<0·01
Lebanon	12	<0·01	23	0·04
Lesotho	6	<0·01	8	<0·01
Liberia	11	<0·01	18	<0·01
Libyan Arab Jamahiriya	18	<0·01	28	<0·01
Lithuania	8	0·20	11	<0·01
Luxembourg	1	<0·01	3	<0·01
Madagascar	59	<0·01	94	<0·01
Malawi	41	<0·01	67	<0·01

Malaysia	78	<0·01	137	<0·01
Maldives	1	<0·01	2	<0·01
Mali	37	<0·01	70	<0·01
Malta	1	<0·01	2	<0·01
Marshall Islands	2	<0·01	2	<0·01
Mauritania	9	<0·01	16	<0·01
Mauritius	4	<0·01	5	<0·01
Mexico	312	<0·01	508	0·01
Micronesia (Federated States of)	2	<0·01	0	<0·01
Monaco	0	<0·01	0	<0·01
Mongolia	8	<0·01	14	<0·01
Montenegro	2	<0·01	2	<0·01
Morocco	92	<0·01	149	<0·01
Mozambique	63	<0·01	106	<0·01
Myanmar	143	<0·01	215	<0·01
Namibia	6	<0·01	9	<0·01
Nauru	0	<0·01	0	<0·01
Nepal	84	<0·01	114	<0·01
Netherlands	0	1·00	0	1·00
New Zealand	0	1·00	0	1·00
Nicaragua	17	<0·01	27	<0·01
Niger	43	<0·01	74	<0·01
Nigeria	439	<0·01	895	<0·01
Niue	0	–	0	–
Norway	0	1·00	0	1·00
Oman	8	<0·01	18	<0·01
Pakistan	484	<0·01	962	<0·01
Palau	0	<0·01	0	<0·01
Panama	10	<0·01	18	<0·01
Papua New Guinea	19	<0·01	30	<0·01
Paraguay	18	<0·01	28	<0·01
Peru	82	<0·01	137	0·01
Philippines	260	<0·01	469	<0·01
Poland	99	0·10	65	0·60
Portugal	27	0·13	0	1·00
Qatar	3	<0·01	8	0·28

Republic of Korea	132	0·06	5	0·97
Republic of Moldova	11	<0·01	14	<0·01
Republic of North Macedonia	6	<0·01	8	<0·01
Romania	62	<0·01	79	0·01
Russian Federation	411	<0·01	573	0·02
Rwanda	29	<0·01	48	<0·01
Saint Kitts and Nevis	0	<0·01	0	<0·01
Saint Lucia	1	<0·01	1	<0·01
Saint Vincent and the Grenadines	0	<0·01	0	<0·01
Samoa	1	<0·01	1	<0·01
San Marino	0	<0·01	0	<0·01
Sao Tome and Principe	1	<0·01	1	<0·01
Saudi Arabia	72	0·01	107	0·28
Senegal	37	<0·01	65	<0·01
Serbia	29	<0·01	28	<0·01
Seychelles	0	<0·01	0	<0·01
Sierra Leone	17	<0·01	25	<0·01
Singapore	13	<0·01	24	<0·01
Slovakia	16	<0·01	23	<0·01
Slovenia	6	<0·01	0	1·00
Solomon Islands	1	<0·01	2	<0·01
Somalia	26	<0·01	43	<0·01
South Africa	137	0·04	207	0·10
South Sudan	31	<0·01	47	<0·01
Spain	0	1·00	0	1·00
Sri Lanka	56	<0·01	87	0·01
Sudan	114	<0·01	180	<0·01
Suriname	1	<0·01	2	<0·01
Sweden	0	1·00	0	1·00
Switzerland	0	1·00	0	1·00
Syrian Arab Republic	59	<0·01	93	<0·01
Tajikistan	20	<0·01	34	<0·01
Thailand	186	<0·01	284	0·01
Timor-Leste	3	<0·01	5	<0·01
Togo	20	<0·01	28	<0·01
Tonga	0	<0·01	0	<0·01

Trinidad and Tobago	4	<0·01	6	<0·01
Tunisia	30	<0·01	50	<0·01
Turkmenistan	14	<0·01	21	<0·01
Tuvalu	0	<0·01	0	<0·01
Türkiye	219	<0·01	328	0·04
Uganda	93	<0·01	155	<0·01
Ukraine	133	<0·01	142	0·03
United Arab Emirates	13	<0·01	22	0·42
United Kingdom	38	0·79	128	0·53
United Republic of Tanzania	120	<0·01	239	<0·01
United States of America	0	1·00	0	1·00
Uruguay	6	0·41	13	0·07
Uzbekistan	81	<0·01	120	<0·01
Vanuatu	1	<0·01	1	<0·01
Venezuela (Bolivarian Republic of)	81	<0·01	115	<0·01
Viet Nam	257	<0·01	374	<0·01
Yemen	67	<0·01	100	<0·01
Zambia	35	<0·01	60	<0·01
Zimbabwe	39	<0·01	58	<0·01

Note: Transplant coverage was calculated as the ratio of actual to expected transplant numbers. The number of missing kidney transplants was estimated as the difference between actual and expected transplant numbers. Countries with coverage ≥1 were capped at 1. Countries with coverage less than 0·01 were presented as "<0·01" for clarity. Missing data are denoted as "–". Data are sorted alphabetically by country. "Global" values represent the aggregate across all included countries and territories.

Appendix 1 Table S27: Trends in Missing Pancreas Transplants and Coverage Rates by Country, 2008–2023

	**2008**		**2023**	
**Country**	**Pancreas Missing**	**Pancreas Coverage**	**Pancreas Missing**	**Pancreas Coverage**
**Global**	11785	0·17	6396	0·25
Afghanistan	59	<0·01	46	<0·01
Albania	7	<0·01	3	<0·01
Algeria	72	<0·01	48	<0·01
Andorra	0	<0·01	0	<0·01
Angola	37	<0·01	24	<0·01
Antigua and Barbuda	0	<0·01	0	<0·01
Argentina	0	1·00	21	0·58

Armenia	6	<0·01	3	<0·01
Australia	12	0·73	0	1·00
Austria	0	1·00	0	1·00
Azerbaijan	18	<0·01	11	<0·01
Bahamas	1	<0·01	0	<0·01
Bahrain	2	<0·01	1	<0·01
Bangladesh	339	<0·01	190	<0·01
Barbados	1	<0·01	0	<0·01
Belarus	18	0·10	10	<0·01
Belgium	4	0·82	4	0·70
Belize	1	<0·01	0	<0·01
Benin	20	<0·01	12	<0·01
Bhutan	1	<0·01	1	<0·01
Bolivia (Plurinational State of)	20	<0·01	14	<0·01
Bosnia and Herzegovina	8	<0·01	4	<0·01
Botswana	4	<0·01	2	<0·01
Brazil	376	0·08	119	0·50
Brunei Darussalam	1	<0·01	0	<0·01
Bulgaria	16	<0·01	7	<0·01
Burkina Faso	32	<0·01	19	<0·01
Burundi	19	<0·01	12	<0·01
Cambodia	31	<0·01	17	<0·01
Cameroon	40	<0·01	26	<0·01
Canada	0	1·00	0	1·00
Cape Verde	1	<0·01	1	<0·01
Central African Republic	9	<0·01	5	<0·01
Chad	23	<0·01	15	<0·01
Chile	35	<0·01	16	0·28
China	2806	<0·01	1501	0·05
Colombia	93	0·05	42	0·26
Comoros	2	<0·01	1	<0·01
Congo	8	<0·01	5	<0·01
Cook Islands	0	<0·01	0	<0·01
Costa Rica	9	<0·01	6	<0·01
Croatia	0	1·00	0	1·00
Cuba	23	0·04	12	<0·01

Cyprus	2	<0·01	1	<0·01
Czech Republic	0	1·00	0	1·00
Côte d'Ivoire	41	<0·01	23	<0·01
Democratic People's Republic of Korea	50	<0·01	28	<0·01
Democratic Republic of The Congo	136	<0·01	76	<0·01
Denmark	12	<0·01	3	0·46
Djibouti	2	<0·01	1	<0·01
Dominica	0	<0·01	0	<0·01
Dominican Republic	21	<0·01	12	<0·01
Ecuador	28	<0·01	20	<0·01
Egypt	161	<0·01	101	<0·01
El Salvador	15	<0·01	7	<0·01
Equatorial Guinea	1	<0·01	1	<0·01
Eritrea	10	<0·01	7	<0·01
Estonia	3	<0·01	0	1·00
Eswatini	2	<0·01	1	<0·01
Ethiopia	179	<0·01	139	<0·01
Fiji	2	<0·01	1	<0·01
Finland	11	<0·01	0	1·00
France	49	0·62	0	1·00
Gabon	3	<0·01	2	<0·01
Gambia	4	<0·01	2	<0·01
Georgia	9	<0·01	4	<0·01
Germany	39	0·77	33	0·64
Ghana	50	<0·01	31	<0·01
Greece	22	0·09	11	<0·01
Grenada	0	<0·01	0	<0·01
Guatemala	29	<0·01	20	<0·01
Guinea	20	<0·01	13	<0·01
Guinea-Bissau	4	<0·01	2	<0·01
Guyana	1	<0·01	1	<0·01
Haiti	21	<0·01	12	<0·01
Honduras	15	<0·01	11	<0·01
Hungary	16	0·24	0	0·98
Iceland	1	<0·01	0	<0·01
India	2491	<0·01	1544	0·02

Indonesia	492	<0·01	278	<0·01
Iran (Islamic Republic of)	139	0·09	57	0·40
Iraq	62	<0·01	38	<0·01
Ireland	0	1·00	0	1·00
Israel	4	0·75	4	0·59
Italy	65	0·48	27	0·59
Jamaica	6	<0·01	3	<0·01
Japan	259	0·04	99	0·27
Jordan	13	<0·01	12	<0·01
Kazakhstan	32	0·03	22	<0·01
Kenya	81	<0·01	61	<0·01
Kiribati	0	<0·01	0	<0·01
Kuwait	5	0·16	3	0·42
Kyrgyzstan	11	<0·01	6	<0·01
Lao People's Democratic Republic	13	<0·01	8	<0·01
Latvia	4	0·21	2	<0·01
Lebanon	9	<0·01	7	<0·01
Lesotho	4	<0·01	2	<0·01
Liberia	8	<0·01	5	<0·01
Libyan Arab Jamahiriya	13	<0·01	8	<0·01
Lithuania	3	0·56	1	0·67
Luxembourg	1	<0·01	1	<0·01
Madagascar	42	<0·01	26	<0·01
Malawi	30	<0·01	18	<0·01
Malaysia	57	<0·01	38	<0·01
Maldives	1	<0·01	0	<0·01
Mali	27	<0·01	19	<0·01
Malta	1	<0·01	1	<0·01
Marshall Islands	1	<0·01	1	<0·01
Mauritania	7	<0·01	4	<0·01
Mauritius	3	<0·01	1	<0·01
Mexico	225	<0·01	141	<0·01
Micronesia (Federated States of)	1	<0·01	0	<0·01
Monaco	0	<0·01	0	<0·01
Mongolia	6	<0·01	4	<0·01
Montenegro	1	<0·01	1	<0·01

Morocco	66	<0·01	41	<0·01
Mozambique	46	<0·01	29	<0·01
Myanmar	103	<0·01	59	<0·01
Namibia	4	<0·01	3	<0·01
Nauru	0	<0·01	0	<0·01
Nepal	60	<0·01	31	<0·01
Netherlands	21	0·40	3	0·83
New Zealand	5	0·45	3	0·52
Nicaragua	12	<0·01	7	<0·01
Niger	31	<0·01	20	<0·01
Nigeria	318	<0·01	246	<0·01
Niue	0	–	0	–
Norway	0	1·00	2	0·66
Oman	6	<0·01	5	<0·01
Pakistan	351	<0·01	265	<0·01
Palau	0	<0·01	0	<0·01
Panama	7	<0·01	5	<0·01
Papua New Guinea	14	<0·01	8	<0·01
Paraguay	13	<0·01	8	<0·01
Peru	59	<0·01	38	<0·01
Philippines	188	<0·01	129	<0·01
Poland	60	0·25	17	0·62
Portugal	8	0·62	0	1·00
Qatar	2	<0·01	3	<0·01
Republic of Korea	84	0·18	33	0·42
Republic of Moldova	8	<0·01	4	<0·01
Republic of North Macedonia	4	<0·01	2	<0·01
Romania	45	<0·01	22	<0·01
Russian Federation	298	<0·01	144	0·10
Rwanda	21	<0·01	13	<0·01
Saint Kitts and Nevis	0	<0·01	0	<0·01
Saint Lucia	0	<0·01	0	<0·01
Saint Vincent and the Grenadines	0	<0·01	0	<0·01
Samoa	0	<0·01	0	<0·01
San Marino	0	<0·01	0	<0·01
Sao Tome and Principe	0	<0·01	0	<0·01

Saudi Arabia	52	0·02	23	0·44
Senegal	27	<0·01	18	<0·01
Serbia	21	<0·01	8	<0·01
Seychelles	0	<0·01	0	<0·01
Sierra Leone	13	<0·01	7	<0·01
Singapore	9	<0·01	6	0·15
Slovakia	11	<0·01	6	<0·01
Slovenia	4	<0·01	2	<0·01
Solomon Islands	1	<0·01	1	<0·01
Somalia	19	<0·01	12	<0·01
South Africa	97	0·05	55	0·13
South Sudan	22	<0·01	13	<0·01
Spain	0	1·00	0	1·00
Sri Lanka	41	<0·01	24	<0·01
Sudan	83	<0·01	49	<0·01
Suriname	1	<0·01	1	<0·01
Sweden	19	<0·01	0	1·00
Switzerland	0	1·00	0	1·00
Syrian Arab Republic	43	<0·01	26	<0·01
Tajikistan	14	<0·01	9	<0·01
Thailand	135	<0·01	76	0·04
Timor-Leste	3	<0·01	1	<0·01
Togo	14	<0·01	8	<0·01
Tonga	0	<0·01	0	<0·01
Trinidad and Tobago	3	<0·01	2	<0·01
Tunisia	22	<0·01	14	<0·01
Turkmenistan	10	<0·01	6	<0·01
Tuvalu	0	<0·01	0	<0·01
Türkiye	149	0·06	93	0·01
Uganda	67	<0·01	43	<0·01
Ukraine	96	<0·01	38	0·05
United Arab Emirates	9	<0·01	7	0·29
United Kingdom	0	1·00	0	1·00
United Republic of Tanzania	87	<0·01	66	<0·01
United States of America	0	1·00	0	1·00
Uruguay	1	0·84	4	<0·01

Uzbekistan	58	<0·01	33	<0·01
Vanuatu	0	<0·01	0	<0·01
Venezuela (Bolivarian Republic of)	59	<0·01	32	<0·01
Viet Nam	186	<0·01	102	0·01
Yemen	49	<0·01	28	<0·01
Zambia	26	<0·01	16	<0·01
Zimbabwe	28	<0·01	16	<0·01

Note: Transplant coverage was calculated as the ratio of actual to expected transplant numbers. The number of missing kidney transplants was estimated as the difference between actual and expected transplant numbers. Countries with coverage ≥1 were capped at 1. Countries with coverage less than 0·01 were presented as "<0·01" for clarity. Missing data are denoted as "–". Data are sorted alphabetically by country. "Global" values represent the aggregate across all included countries and territories.

Appendix 1 Table S28: Trends in Missing Small Bowel Transplants and Coverage Rates by Country, 2008–2023

	**2008**		**2023**	
**Country**	**Small Bowel Missing**	**Small Bowel Coverage**	**Small Bowel Missing**	**Small Bowel Coverage**
**Global**	1089	0·19	587	0·24
Afghanistan	6	<0·01	4	<0·01
Albania	1	<0·01	0	<0·01
Algeria	7	<0·01	4	<0·01
Andorra	0	<0·01	0	<0·01
Angola	4	<0·01	2	<0·01
Antigua and Barbuda	0	<0·01	0	<0·01
Argentina	0	1·00	3	0·44
Armenia	1	<0·01	0	<0·01
Australia	4	<0·01	2	0·38
Austria	2	<0·01	1	<0·01
Azerbaijan	2	<0·01	1	<0·01
Bahamas	0	<0·01	0	<0·01
Bahrain	0	<0·01	0	<0·01
Bangladesh	32	<0·01	17	<0·01
Barbados	0	<0·01	0	<0·01
Belarus	2	<0·01	1	<0·01
Belgium	2	<0·01	0	1·00
Belize	0	<0·01	0	<0·01
Benin	2	<0·01	1	<0·01

Bhutan	0	<0·01	0	<0·01
Bolivia (Plurinational State of)	2	<0·01	1	<0·01
Bosnia and Herzegovina	1	<0·01	0	<0·01
Botswana	0	<0·01	0	<0·01
Brazil	39	<0·01	21	0·05
Brunei Darussalam	0	<0·01	0	<0·01
Bulgaria	2	<0·01	1	<0·01
Burkina Faso	3	<0·01	2	<0·01
Burundi	2	<0·01	1	<0·01
Cambodia	3	<0·01	2	<0·01
Cameroon	4	<0·01	2	<0·01
Canada	3	0·60	0	1·00
Cape Verde	0	<0·01	0	<0·01
Central African Republic	1	<0·01	0	<0·01
Chad	2	<0·01	1	<0·01
Chile	3	<0·01	2	<0·01
China	267	<0·01	131	0·08
Colombia	9	<0·01	4	0·19
Comoros	0	<0·01	0	<0·01
Congo	1	<0·01	0	<0·01
Cook Islands	0	<0·01	0	<0·01
Costa Rica	1	<0·01	1	<0·01
Croatia	1	<0·01	0	<0·01
Cuba	2	<0·01	1	<0·01
Cyprus	0	<0·01	0	<0·01
Czech Republic	2	<0·01	1	<0·01
Côte d’Ivoire	4	<0·01	2	<0·01
Democratic People's Republic of Korea	5	<0·01	2	<0·01
Democratic Republic of The Congo	13	<0·01	7	<0·01
Denmark	1	<0·01	1	<0·01
Djibouti	0	<0·01	0	<0·01
Dominica	0	<0·01	0	<0·01
Dominican Republic	2	<0·01	1	<0·01
Ecuador	3	<0·01	2	<0·01
Egypt	15	<0·01	9	<0·01
El Salvador	1	<0·01	1	<0·01

Equatorial Guinea	0	<0·01	0	<0·01
Eritrea	1	<0·01	1	<0·01
Estonia	0	<0·01	0	<0·01
Eswatini	0	<0·01	0	<0·01
Ethiopia	17	<0·01	13	<0·01
Fiji	0	<0·01	0	<0·01
Finland	1	<0·01	0	1·00
France	0	1·00	5	0·15
Gabon	0	<0·01	0	<0·01
Gambia	0	<0·01	0	<0·01
Georgia	1	<0·01	0	<0·01
Germany	16	<0·01	7	0·12
Ghana	5	<0·01	3	<0·01
Greece	2	<0·01	1	<0·01
Grenada	0	<0·01	0	<0·01
Guatemala	3	<0·01	2	<0·01
Guinea	2	<0·01	1	<0·01
Guinea-Bissau	0	<0·01	0	<0·01
Guyana	0	<0·01	0	<0·01
Haiti	2	<0·01	1	<0·01
Honduras	1	<0·01	1	<0·01
Hungary	2	<0·01	1	<0·01
Iceland	0	<0·01	0	<0·01
India	237	<0·01	127	0·11
Indonesia	47	<0·01	25	<0·01
Iran (Islamic Republic of)	14	<0·01	0	1·00
Iraq	6	<0·01	3	<0·01
Ireland	1	<0·01	1	<0·01
Israel	0	0·71	1	<0·01
Italy	9	0·25	5	0·17
Jamaica	1	<0·01	0	<0·01
Japan	25	0·04	9	0·24
Jordan	1	<0·01	1	<0·01
Kazakhstan	3	<0·01	2	<0·01
Kenya	8	<0·01	6	<0·01
Kiribati	0	<0·01	0	<0·01

Kuwait	1	<0·01	0	<0·01
Kyrgyzstan	1	<0·01	1	<0·01
Lao People's Democratic Republic	1	<0·01	1	<0·01
Latvia	0	<0·01	0	<0·01
Lebanon	1	<0·01	1	<0·01
Lesotho	0	<0·01	0	<0·01
Liberia	1	<0·01	0	<0·01
Libyan Arab Jamahiriya	1	<0·01	1	<0·01
Lithuania	1	<0·01	0	<0·01
Luxembourg	0	<0·01	0	<0·01
Madagascar	4	<0·01	2	<0·01
Malawi	3	<0·01	2	<0·01
Malaysia	5	<0·01	3	<0·01
Maldives	0	<0·01	0	<0·01
Mali	3	<0·01	2	<0·01
Malta	0	<0·01	0	<0·01
Marshall Islands	0	<0·01	0	<0·01
Mauritania	1	<0·01	0	<0·01
Mauritius	0	<0·01	0	<0·01
Mexico	22	<0·01	13	<0·01
Micronesia (Federated States of)	0	<0·01	0	<0·01
Monaco	0	<0·01	0	<0·01
Mongolia	1	<0·01	0	<0·01
Montenegro	0	<0·01	0	<0·01
Morocco	6	<0·01	4	<0·01
Mozambique	4	<0·01	3	<0·01
Myanmar	10	<0·01	5	<0·01
Namibia	0	<0·01	0	<0·01
Nauru	0	<0·01	0	<0·01
Nepal	6	<0·01	3	<0·01
Netherlands	3	<0·01	1	0·57
New Zealand	1	<0·01	1	<0·01
Nicaragua	1	<0·01	1	<0·01
Niger	3	<0·01	2	<0·01
Nigeria	30	<0·01	22	<0·01
Niue	0	–	0	–

Norway	1	<0·01	1	<0·01
Oman	1	<0·01	0	<0·01
Pakistan	33	<0·01	24	<0·01
Palau	0	<0·01	0	<0·01
Panama	1	<0·01	0	<0·01
Papua New Guinea	1	<0·01	1	<0·01
Paraguay	0	0·81	1	<0·01
Peru	6	<0·01	3	<0·01
Philippines	18	<0·01	12	<0·01
Poland	8	<0·01	4	<0·01
Portugal	2	<0·01	1	<0·01
Qatar	0	<0·01	0	<0·01
Republic of Korea	10	<0·01	5	<0·01
Republic of Moldova	1	<0·01	0	<0·01
Republic of North Macedonia	0	<0·01	0	<0·01
Romania	4	<0·01	2	<0·01
Russian Federation	28	<0·01	14	0·07
Rwanda	2	<0·01	1	<0·01
Saint Kitts and Nevis	0	<0·01	0	<0·01
Saint Lucia	0	<0·01	0	<0·01
Saint Vincent and the Grenadines	0	<0·01	0	<0·01
Samoa	0	<0·01	0	<0·01
San Marino	0	<0·01	0	<0·01
Sao Tome and Principe	0	<0·01	0	<0·01
Saudi Arabia	5	<0·01	1	0·81
Senegal	3	<0·01	2	<0·01
Serbia	2	<0·01	1	<0·01
Seychelles	0	<0·01	0	<0·01
Sierra Leone	1	<0·01	1	<0·01
Singapore	1	<0·01	1	<0·01
Slovakia	1	<0·01	1	<0·01
Slovenia	0	<0·01	0	<0·01
Solomon Islands	0	<0·01	0	<0·01
Somalia	2	<0·01	1	<0·01
South Africa	10	<0·01	6	<0·01
South Sudan	2	<0·01	1	<0·01

Spain	0	1·00	0	1·00
Sri Lanka	4	<0·01	2	<0·01
Sudan	8	<0·01	4	<0·01
Suriname	0	<0·01	0	<0·01
Sweden	2	<0·01	0	1·00
Switzerland	0	0·67	1	<0·01
Syrian Arab Republic	4	<0·01	2	<0·01
Tajikistan	1	<0·01	1	<0·01
Thailand	13	<0·01	7	<0·01
Timor-Leste	0	<0·01	0	<0·01
Togo	1	<0·01	1	<0·01
Tonga	0	<0·01	0	<0·01
Trinidad and Tobago	0	<0·01	0	<0·01
Tunisia	2	<0·01	1	<0·01
Turkmenistan	1	<0·01	1	<0·01
Tuvalu	0	<0·01	0	<0·01
Türkiye	12	0·20	8	0·12
Uganda	6	<0·01	4	<0·01
Ukraine	9	<0·01	4	<0·01
United Arab Emirates	1	<0·01	1	<0·01
United Kingdom	0	1·00	0	1·00
United Republic of Tanzania	8	<0·01	6	<0·01
United States of America	0	1·00	0	1·00
Uruguay	1	<0·01	0	<0·01
Uzbekistan	6	<0·01	3	<0·01
Vanuatu	0	<0·01	0	<0·01
Venezuela (Bolivarian Republic of)	6	<0·01	3	<0·01
Viet Nam	6	0·68	9	<0·01
Yemen	5	<0·01	2	<0·01
Zambia	2	<0·01	2	<0·01
Zimbabwe	3	<0·01	1	<0·01

Note: Transplant coverage was calculated as the ratio of actual to expected transplant numbers. The number of missing kidney transplants was estimated as the difference between actual and expected transplant numbers. Countries with coverage ≥1 were capped at 1. Countries with coverage less than 0·01 were presented as “<0·01” for clarity. Missing data are denoted as “–”. Data are sorted alphabetically by country. “Global” values represent the aggregate across all included countries and territories.

Section 4. Figures

Appendix 1 Figure S1: Missingness of key transplant variables in the GODT dataset, 2008–2023

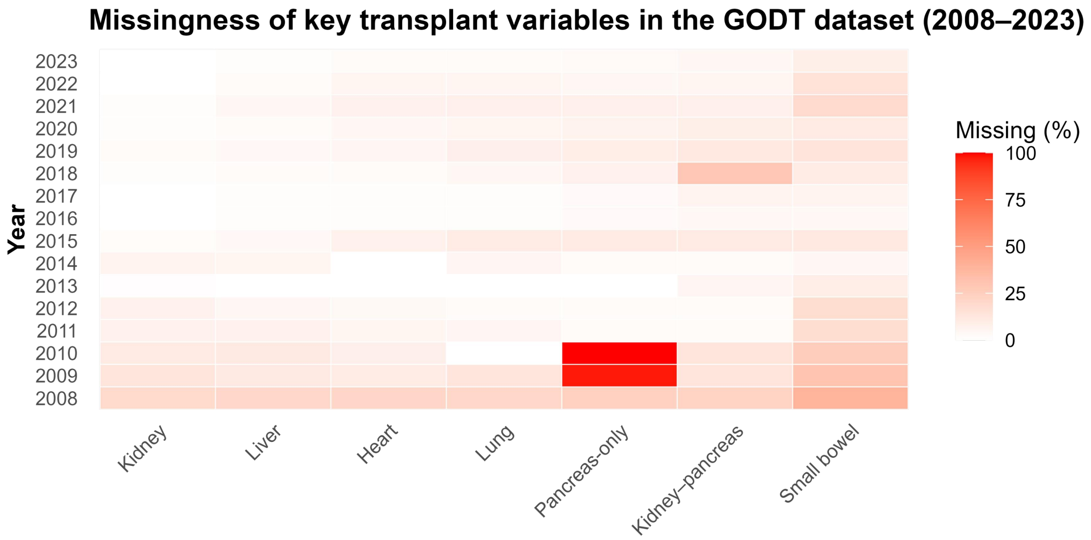

Missingness was calculated only among countries with documented transplant activity; structural zeroes (countries without capacity) were excluded. Values represent the proportion of expected reports that were not submitted for each organ and year.

Appendix 1 Figure S2: Joinpoint regression analysis of global transplant rates, 2008–2023

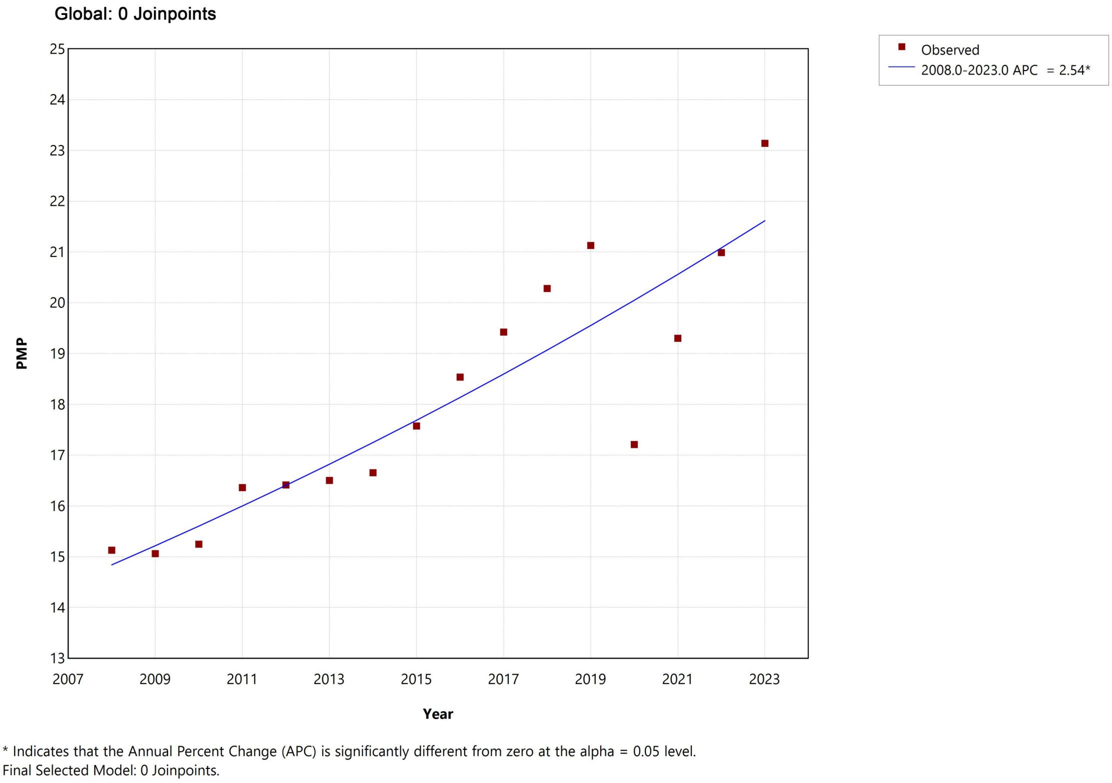

Annual global transplant rates, expressed as transplants per million population (PMP), were analysed using the National Cancer Institute's Joinpoint Regression Program. The analysis identified no joinpoints over the study period, indicating a single linear trend. The solid line represents the fitted joinpoint regression line, and squares denote observed annual values.

Appendix 1 Figure S3: Joinpoint regression analysis of global transplant rates by HDI group, 2008–2023

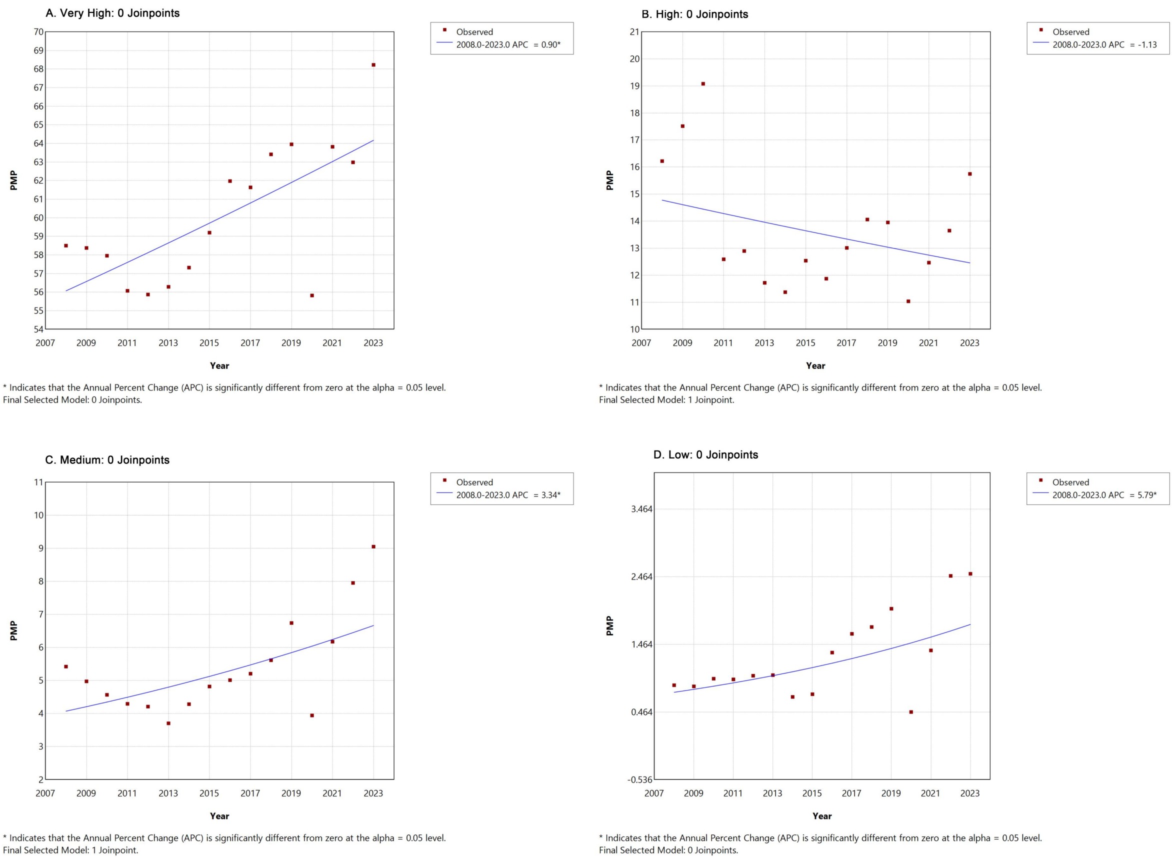

Annual transplant rates, expressed as transplants per million population (PMP), were analysed by Human Development Index (HDI) group using Joinpoint regression with a 0-joinpoint log-linear model. Panels A–D correspond to very high, high, medium, and low HDI groups, respectively. Solid lines represent fitted Joinpoint regression lines, and squares denote observed annual values.

Appendix 1 Figure S4: Joinpoint regression analysis of global transplant rates by WHO region, 2008–2023

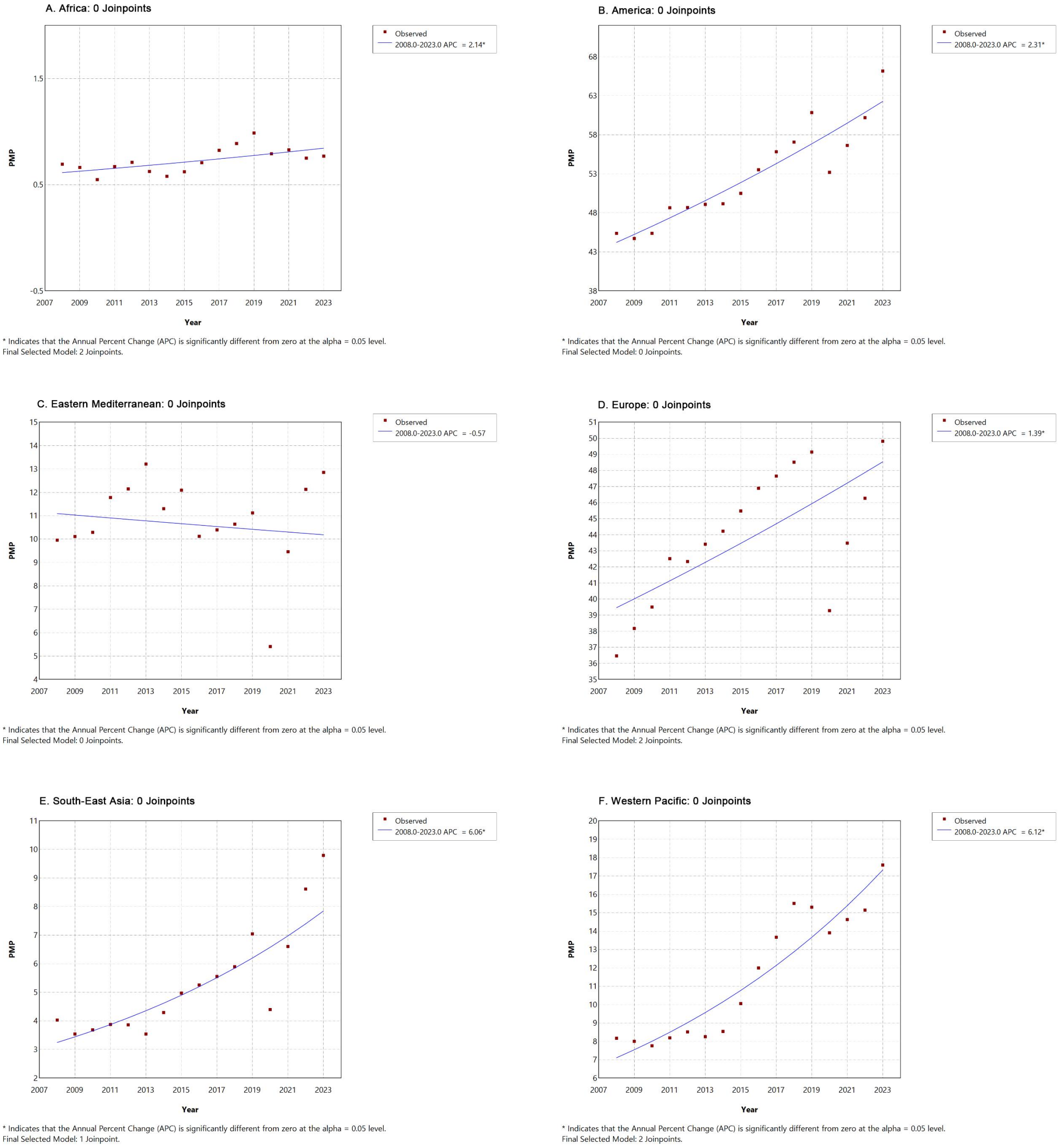

Annual transplant rates, expressed as transplants per million population (PMP), were analysed by WHO region using Joinpoint regression with a 0-joinpoint log-linear model. Panels A–F correspond to Africa, the Americas, Eastern Mediterranean, Europe, South-East Asia, and Western Pacific regions, respectively. Solid lines represent fitted Joinpoint regression lines, and squares denote observed annual values.

Appendix 1 Figure S5: Kidney transplantation in Iran, 2008–2023—counts and donor-type composition

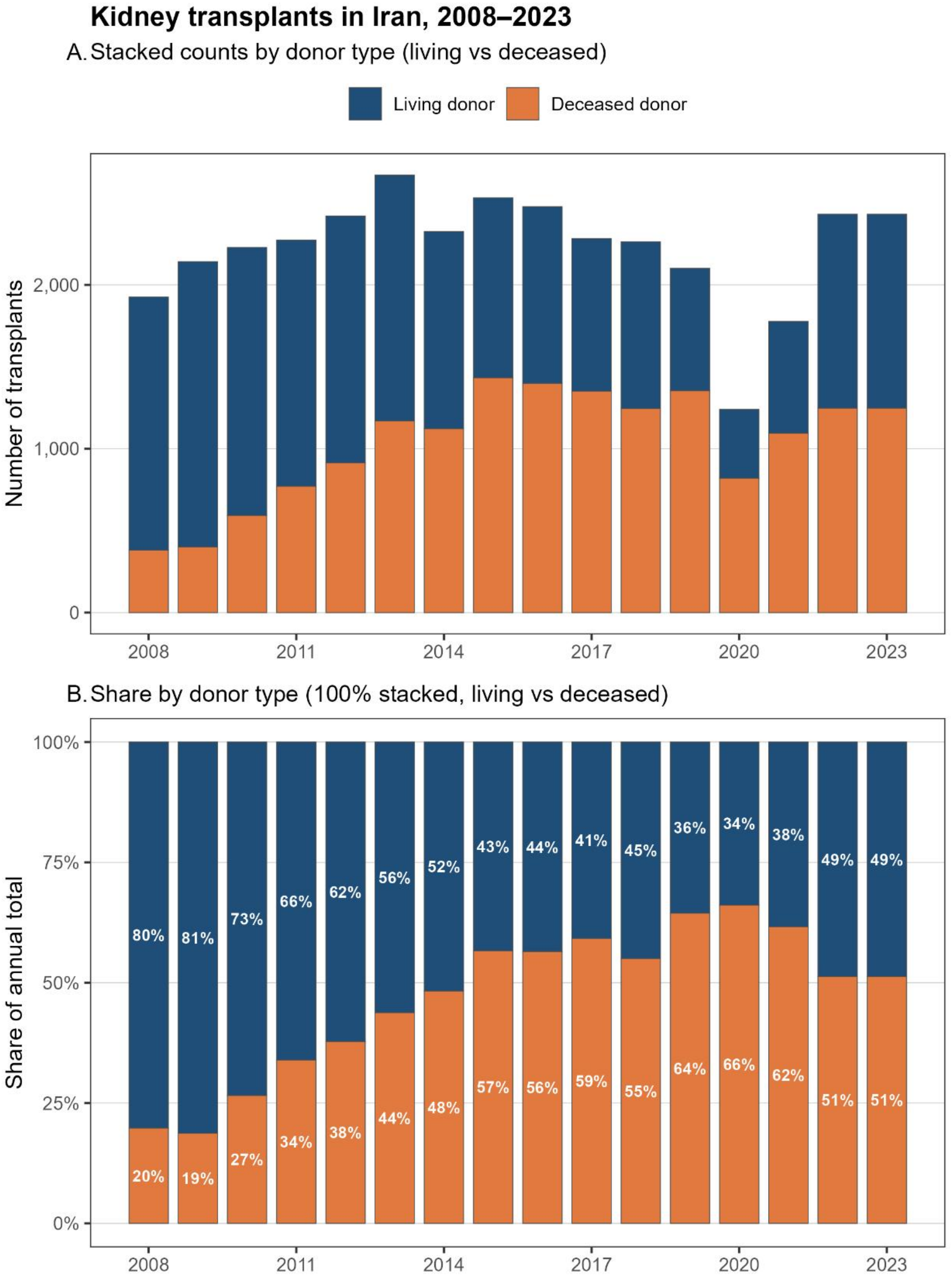

(A) Annual counts by donor type (living vs deceased). (B) Donor-type share of the annual total (100% stacked). Percent labels denote the proportion of yearly kidney transplants attributed to each donor type. 2023 values were estimated because 2023 data were not reported.

Appendix 1 Figure S6: Trends in organ transplant rate per million population (PMP) by HDI group and WHO region, 2008–2023

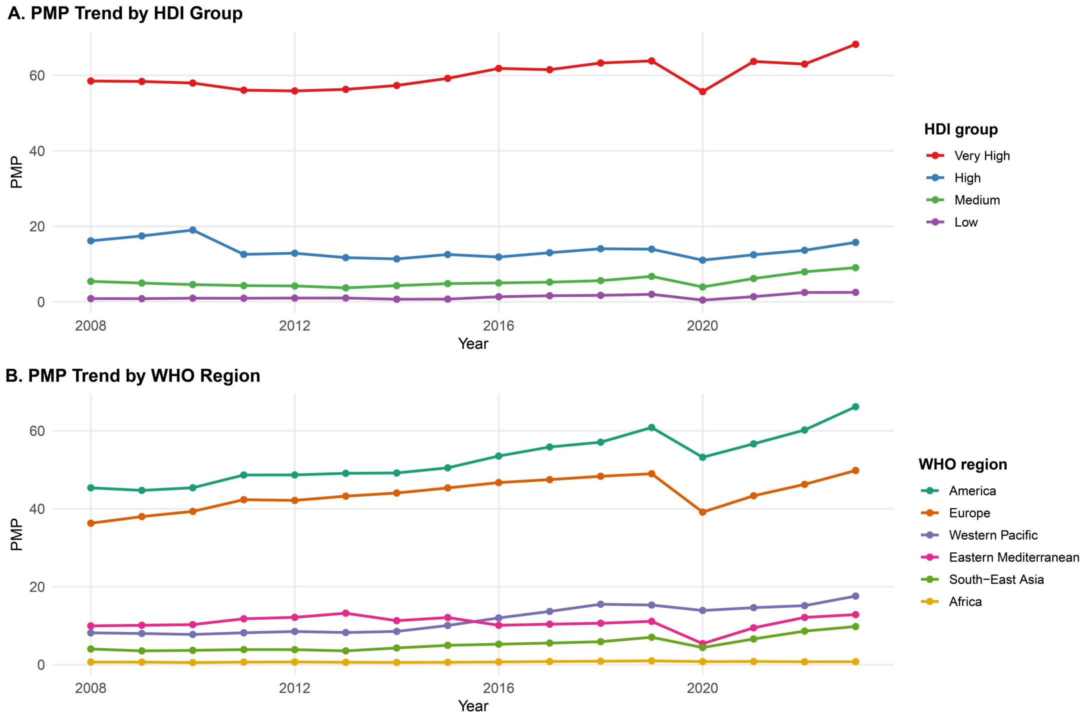

(A) Human Development Index (HDI) groups (very high, high, medium, low). (B) WHO regions (Region of the Americas, Europe, Western Pacific, Eastern Mediterranean, South-East Asia, Africa). Lines show annual group-level PMP. PMP=procedures per million population; HDI=Human Development Index; WHO=World Health Organization.

Appendix 1 Figure S7: Trends in solid organ transplantation PMP: Japan vs global and selected high-HDI countries, 2008–2023

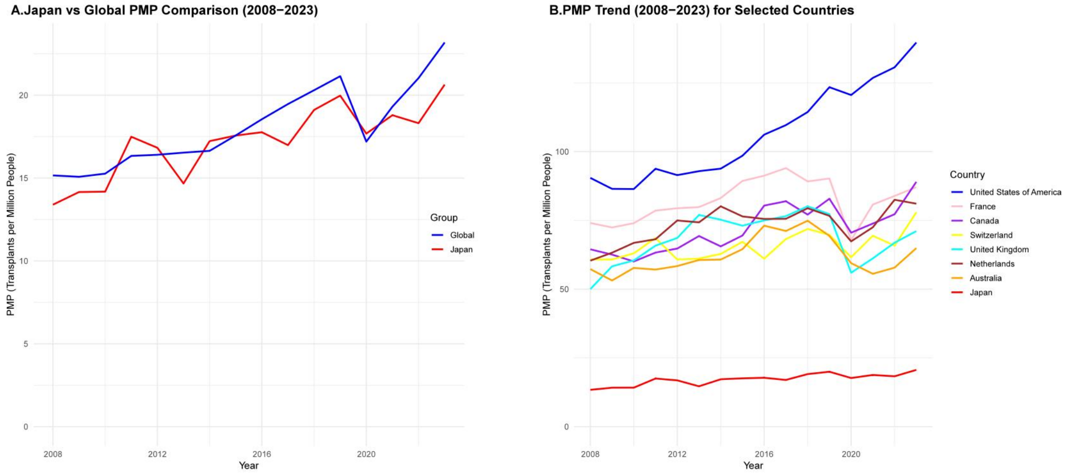

(A) Japan versus global average. Transplantation activity in Japan (PMP) rose modestly over time and was below the global mean for most years of 2008–2023, with occasional convergence in several years. (B) Japan compared with countries of similar HDI (United States, France, Canada, Switzerland, United Kingdom, Netherlands, Australia). These peer countries showed persistently higher PMP and steadier growth, whereas Japan remained substantially lower across the period, underscoring a persistent gap relative to HDI-comparable settings. Abbreviation: PMP = transplants per million population.

Appendix 1 Figure S8: Organ-specific trends in transplantation in China, 2008–2023

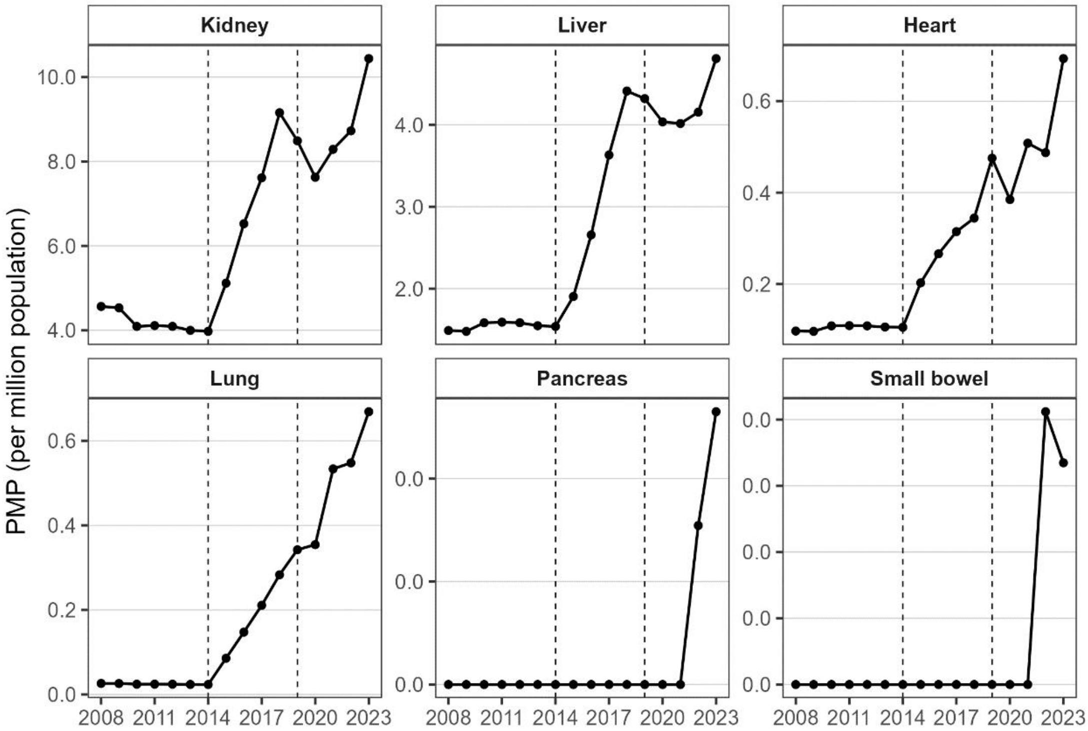

Panels show annual PMP by organ. Points and lines denote yearly values. Dashed vertical lines mark policy years—2015 (policy reform) and 2020 (COVID-19 pandemic)—and define three segments (2008–2014, 2015–2019, 2020–2023) used to estimate segment APCs and an overall AAPC. PMP = per million population; APC = annual percent change; AAPC = average annual percent change.

Appendix 1 Figure S9: Contribution of HDI regions to global organ transplants, 2023

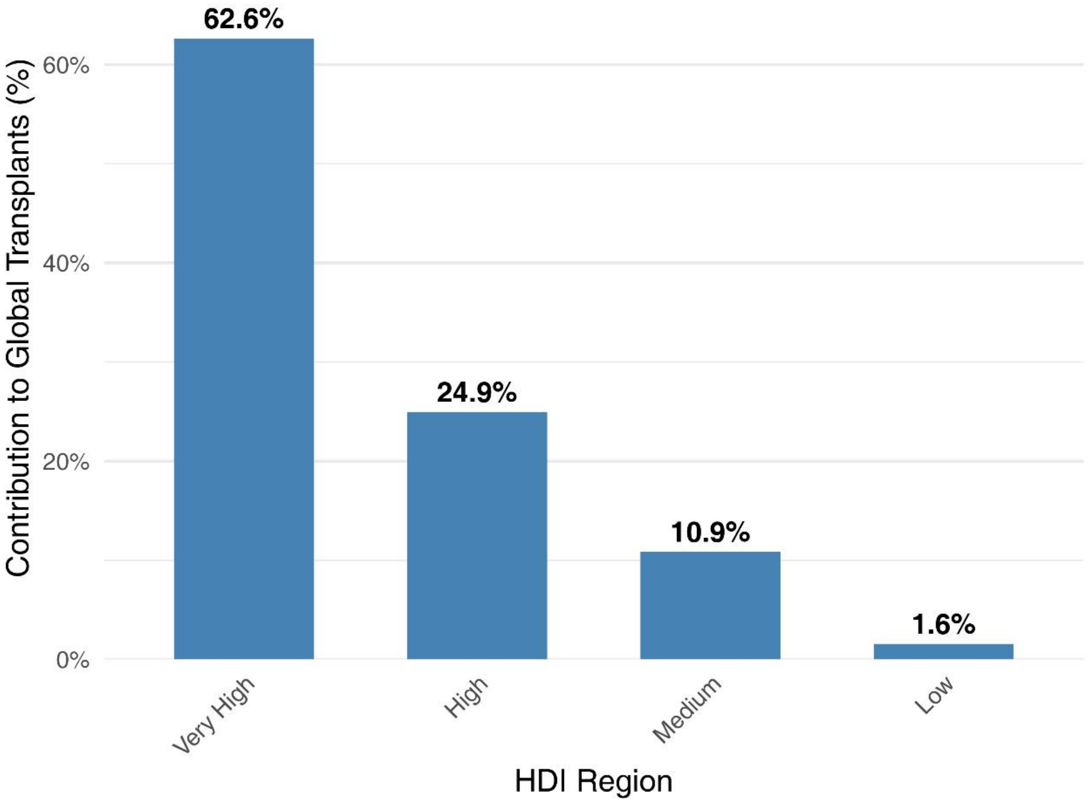

Percentage contribution of very high, high, medium, and low Human Development Index (HDI) regions to the global number of organ transplants in 2023.

Appendix 1 Figure S10: Organ transplant PMP across GBD21 regions in 2008 and 2023

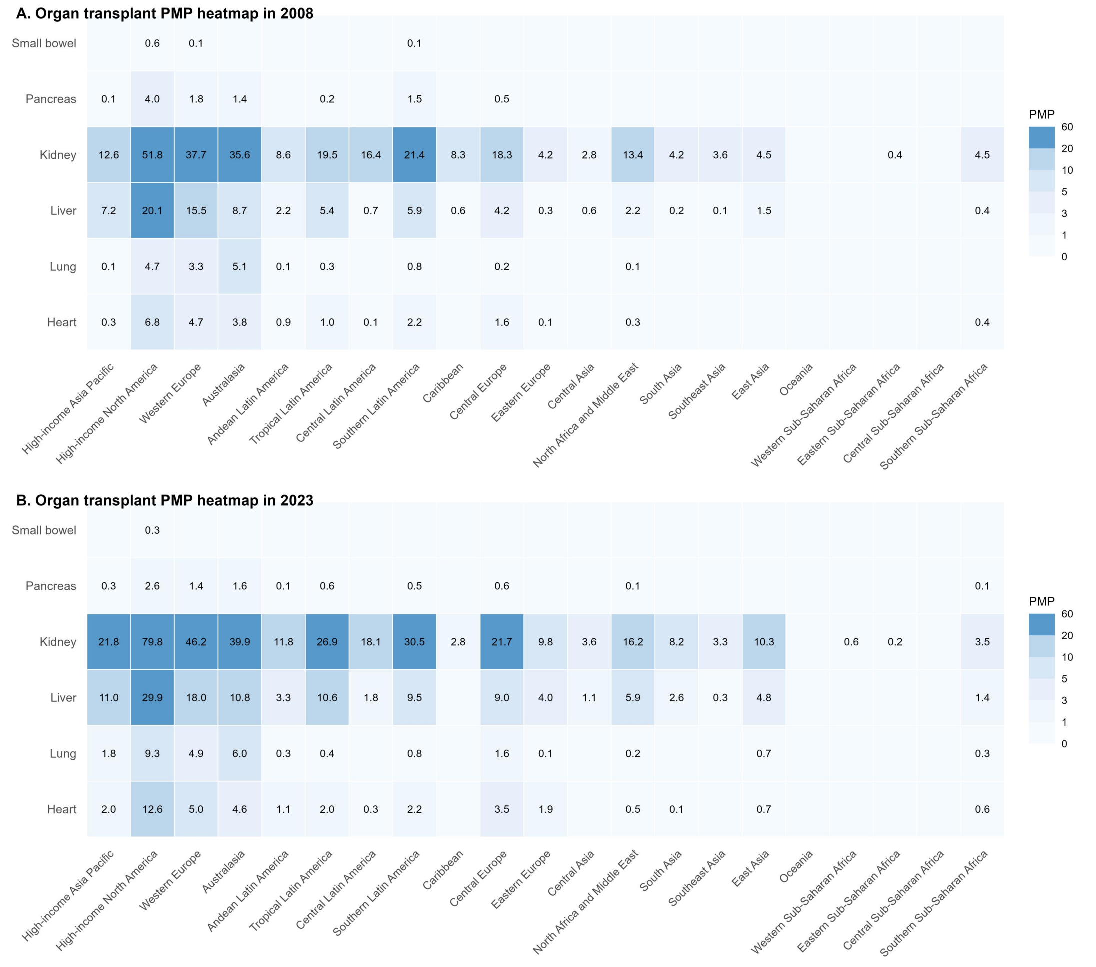

(A) Organ transplant PMP heatmap in 2008. (B) Organ transplant PMP heatmap in 2023. The heatmaps display the number of transplants per million population (PMP) for kidney, liver, heart, lung, pancreas, and small bowel across the 21 GBD regions. Darker shades indicate higher PMP values.

Appendix 1 Figure S11: National transplant trajectories and trends in relation to HDI, 2008–2023

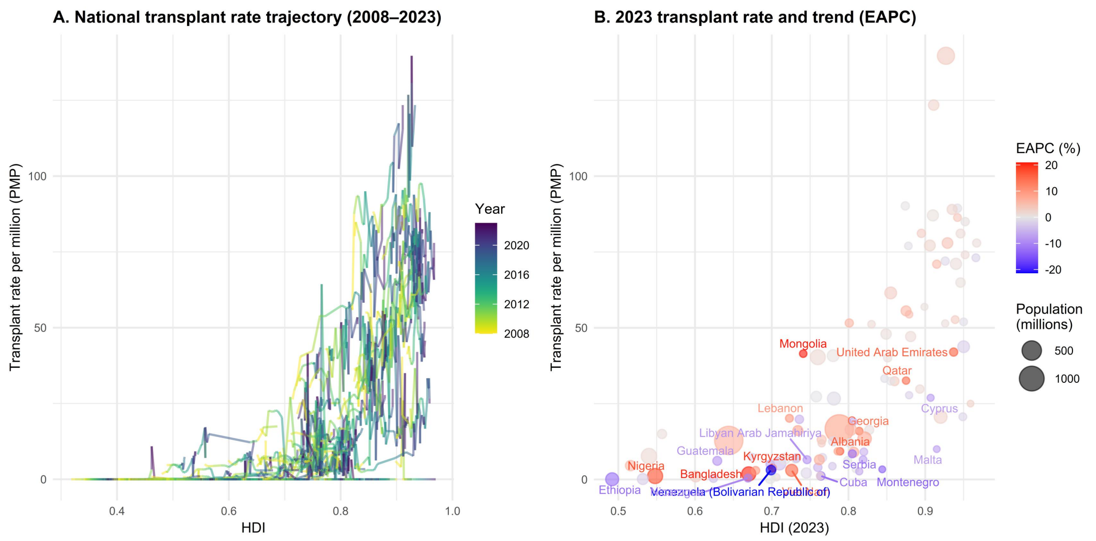

(A) National transplant rate trajectories between 2008 and 2023, expressed as transplants per million population (PMP) across different levels of Human Development Index (HDI). Each line represents one country, colored by calendar year. (B) National transplant rate in 2023 plotted against HDI, with estimated annual percentage change (EAPC) indicated by color (red = increase, blue = decrease). Bubble size reflects national population in 2023. Selected countries are labeled for reference.

Appendix 1 Figure S12: Distribution of solid organ transplants by HDI group, 2023

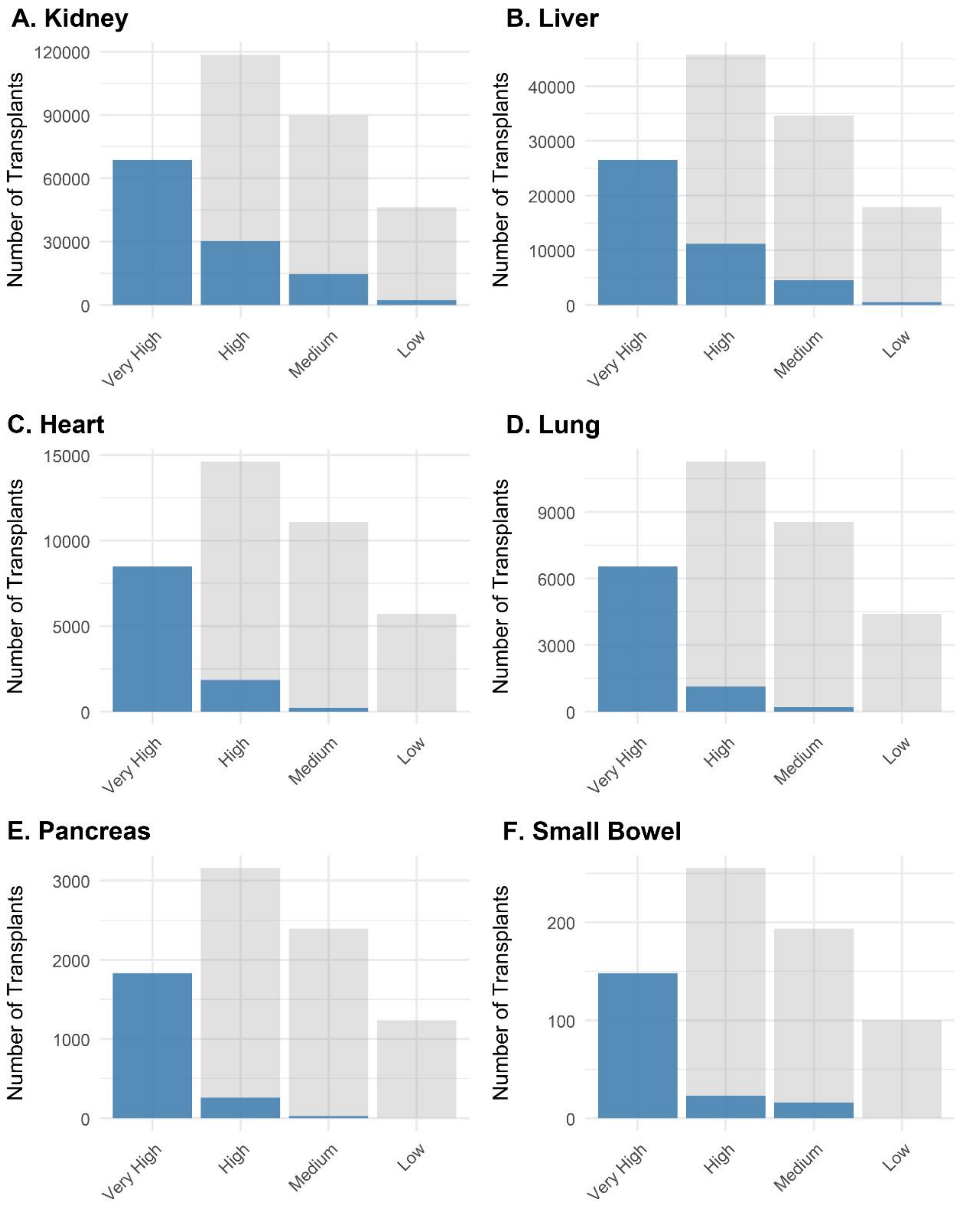

Number of transplants performed in 2023 across Human Development Index (HDI) categories for six solid organs: (A) kidney, (B) liver, (C) heart, (D) lung, (E) pancreas, and (F) small bowel. Bars in blue represent observed transplant activity, and grey bars indicate the estimated need based on reference PMP values. HDI groups are classified as very high, high, medium, and low.

Section 5. Supplementary References

1 Global Observatory on Donation and Transplantation (GODT). Global Observatory on Donation and Transplantation [Internet]. Madrid (ES): Spanish National Transplant Organization (ONT) in collaboration with WHO; Available from: https://www.transplant-observatory.org/.

2 Mahillo B, Carmona M, Álvarez M, Noel L, Matesanz R. Global database on donation and transplantation: goals, methods and critical issues (www.transplant-observatory.org). Transplant Rev 2013; 27: 57–60.

3 Global Observatory on Donation and Transplantation (GODT). Methodology [Internet]. Madrid (ES): Spanish National Transplant Organization (ONT) in collaboration with WHO; Available from: https://www.transplant-observatory.org/methodology/.

4 Ni X, Li Z, Li X, et al. Socioeconomic inequalities in cancer incidence and access to health services among children and adolescents in China: a cross-sectional study. Lancet 2022; 400: 1020–32.

5 Brannigan MC. A chronicle of organ transplant progress in Japan. Transplant Int 1992; 5: 180–6.

6 Eguchi S, Hibi T, Egawa H. Liver transplantation: japanese contributions. J Gastroenterol 2018; 53: 1107–8.

7 Aita K. New organ transplant policies in Japan, including the family-oriented priority donation clause. Transplantation 2011; 91: 489–91.

8 Malek-Hosseini SA, Habibzadeh F, Nikeghbalian S. Shiraz organ transplant center: the largest liver transplant center in the world. Transplantation 2019; 103: 1523–5.

9 Haghighi AN, Ghobadi O, Najafizadeh K, Noohi F, Broumand B, Fazel I. 20 years iranian experience of organ donation: shifting from compensated living unrelated kidney transplantation. Kidney Int Rep 2025; 10: 979–82.

10 Chen Z, Zeng F, Ming C, Ma J, Jiang J. Current situation of organ donation in China—from stigma to stigmata. Am J Transplant. 2006;6(suppl):437. (Abstract; World Transplant Congress, Boston, July 22–27, 2006.)

11 Huang J. The “chinese mode” of organ donation and transplantation. Hepatobiliary Surg Nutr 2017; 6: 246–57.

12 Guo Y. The “chinese mode” of organ donation and transplantation: moving towards the center stage of the world. Hepatobiliary Surg Nutr 2018; 7: 61–2.

13 Guo Y. Erratum to the “chinese mode” of organ donation and transplantation: moving towards the center stage of the world. Hepatobiliary Surg Nutr 2019; 8: 87–87.

14 Naghavi M, Ong KL, Aali A, et al. Global burden of 288 causes of death and life expectancy

decomposition in 204 countries and territories and 811 subnational locations, 1990–2021: a systematic analysis for the global burden of disease study 2021. Lancet 2024; 403: 2100–32.

15 Ferrari AJ, Santomauro DF, Aali A, et al. Global incidence, prevalence, years lived with disability (YLDs), disability-adjusted life-years (DALYs), and healthy life expectancy (HALE) for 371 diseases and injuries in 204 countries and territories and 811 subnational locations, 1990–2021: a systematic analysis for the global burden of disease study 2021. Lancet 2024; 403: 2133–61.

16 Stevens GA, Alkema L, Black RE, et al. Guidelines for accurate and transparent health estimates reporting: the GATHER statement. Lancet 2016; 388: e19–23.
